# Spacemake: processing and analysis of large-scale spatial transcriptomics data

**DOI:** 10.1093/gigascience/giac064

**Published:** 2022-07-19

**Authors:** Tamas Ryszard Sztanka-Toth, Marvin Jens, Nikos Karaiskos, Nikolaus Rajewsky

**Affiliations:** Systems Biology of Gene Regulatory Elements, Max-Delbrück-Center for Molecular Medicine in the Helmholtz Association (MDC), Berlin Institute for Medical Systems Biology (BIMSB), 10115 Berlin, Germany; Humboldt-Universität zu Berlin, Institut für Biologie, 10099 Berlin, Germany; Systems Biology of Gene Regulatory Elements, Max-Delbrück-Center for Molecular Medicine in the Helmholtz Association (MDC), Berlin Institute for Medical Systems Biology (BIMSB), 10115 Berlin, Germany; Systems Biology of Gene Regulatory Elements, Max-Delbrück-Center for Molecular Medicine in the Helmholtz Association (MDC), Berlin Institute for Medical Systems Biology (BIMSB), 10115 Berlin, Germany; Systems Biology of Gene Regulatory Elements, Max-Delbrück-Center for Molecular Medicine in the Helmholtz Association (MDC), Berlin Institute for Medical Systems Biology (BIMSB), 10115 Berlin, Germany; Humboldt-Universität zu Berlin, Institut für Biologie, 10099 Berlin, Germany; DZHK (German Center for Cardiovascular Research), Partner Site Berlin, 10117 Berlin, Germany; Department of Pediatric Oncology, Universitätsmedizin Charité, 13353 Berlin, Germany

**Keywords:** bioinformatics, computational biology, computational pipeline, sequence analysis, spatial transcriptomics, single-cell transcriptomics, reproducibility, modularity, scalability, workflow

## Abstract

**Background:**

Spatial sequencing methods increasingly gain popularity within RNA biology studies. State-of-the-art techniques quantify messenger RNA expression levels from tissue sections and at the same time register information about the original locations of the molecules in the tissue. The resulting data sets are processed and analyzed by accompanying software that, however, is incompatible across inputs from different technologies.

**Findings:**

Here, we present spacemake, a modular, robust, and scalable spatial transcriptomics pipeline built in Snakemake and Python. Spacemake is designed to handle all major spatial transcriptomics data sets and can be readily configured for other technologies. It can process and analyze several samples in parallel, even if they stem from different experimental methods. Spacemake's unified framework enables reproducible data processing from raw sequencing data to automatically generated downstream analysis reports. Spacemake is built with a modular design and offers additional functionality such as sample merging, saturation analysis, and analysis of long reads as separate modules. Moreover, spacemake employs novoSpaRc to integrate spatial and single-cell transcriptomics data, resulting in increased gene counts for the spatial data set. Spacemake is open source and extendable, and it can be seamlessly integrated with existing computational workflows.

## Background

Tremendous advances during the past decade have led to high-throughput single-cell RNA sequencing (scRNA-seq) technologies that became the state of the art for dissecting cellular heterogeneity within tissues. Spatial transcriptomics sequencing (STS) technologies present a further vital development that allows the assignment of single molecules to spatial positions, thus obtaining coordinates of gene expression. When spatial resolution is high enough to discern individual cells, this enables the identification of cell types and their interactions in spatial context. Spatial information is crucial in studying cell–cell communication mechanisms within the native tissue context and can yield new insights in disease states [1]. Recently published array-based methods are able to retain spatial information at different resolutions. Slide-seq (and Slide-seqV2) operates with 10-*μ*m beads that are evenly and randomly distributed on a 2-dimensional surface termed “puck” [2, 3]. This size roughly corresponds to single-cell resolution. Other methods, such as spatial transcriptomics or the commercially available 10X Visium, work with a grid of 100-*μ*m diameter spots, regularly placed on a square glass (with a 200-*μ*m distance between the centers), or 55-*μ*m diameter spots with a 100-*μ*m distance between the centers, respectively [4,5]. These methods usually capture between 1 and 10 cells per spot, depending on the cellular density of the studied tissue. In more recent publications, high-definition spatial transcriptomics recovers gene expression at a 2-*μ*m spatial resolution [6], while MiSeq Illumina flowcells were used to sequence mouse colon and liver tissues, achieving subcellular spatial resolution [7]. Fluorescent RNA labeling methods also achieve very high, often subcellular resolution but operate on only a preselected panel of genes and are hence restricted to targeted studies of gene expression [1,8, 9].

Akin to a technological revolution that took place with the advance of RNA-seq and scRNA-seq, we anticipate STS techniques to become invaluable for better understanding biological processes and mechanisms that lead to diseased states. Dissection of a tumor's transcriptional heterogeneity is a prime example. Tumor progression is an intricate process that involves the coexistence of several cell types within the tumor, such as immune cells, native tissue cell types, and abnormally growing tumor cells. While scRNA-seq can accurately identify different cell types and their transcriptional programs, all spatial information regarding the cellular communication across cell types is lost. This information is critical to characterize spatial interactions within the tumor microenvironment and identify the mechanisms that create suitable conditions for the further progression of the disease, such as angiogenesis and hypoxia.

The various array-based STS methods differ not only in their experimental procedures but also in the data they output and the associated software provided to process and analyze the raw data. Therefore, researchers who wish to take advantage of multiple methods need to get acquainted with several computational pipelines that operate with different logic and output structures. Such a situation can be time-consuming and perplexing, and it can lead to the accumulation of errors when alternating between the different methods. There are a few computational processing tools available to date, namely, the spaceranger from 10X [5], the ST pipeline [10], and slideseq-tools [2, 3, 10]. These tools, however, were developed for one specific STS technology (ST pipeline and spaceranger for Visium and slideseq-tools for Slide-seq data sets) and are therefore not accommodating different types of data. Furthermore, they lack a unified framework to enable simultaneous processing of many different samples. Finally, they lack additional functionality, such as subsampling or merging of samples, integration of scRNA-seq with spatial data sets, or support for troubleshooting of sequencing library construction by using long-read sequencing (Table 1).

**Table 1:** Comparison of spacemake with other published spatial transcriptomics pipelines

		**Slideseq-tools**	**Spaceranger**	**ST pipeline**	**Spacemake**
**Input data**	Slide-seqV2	✓	✗	✓	✓
	10X Visium	✗	✓	✓	✓
	Seq-scope	✗	✗	✗	✓
	Other array-based STS	✗	✗	✗	✓
	Fluorescence in situ hybridization	✗	✗	✗	✗
**Pipeline**	Customizable processing mode	✗	✗	✗	✓
	H&E integration	✗	✓	✗	✓
	Structured output	✓	✓	✓	✓
	Parallel sample processing	✗	✗	✗	✓
	Graphic quality control reports	✓	✓	✗	✓
	Automated downstream analysis	✗	✓	✗	✓
**Additional modules**	Saturation analysis	✓	✓	✓	✓
	Technical replicate merging	✗	✗	✗	✓
	novoSpaRc integration	✗	✗	✗	✓
	Pacbio reads for troubleshooting	✗	✗	✗	✓
**General aspects**	Open source	✓	✗	✓	✓
	Extendable	✓	✗	✓	✓

Here, we present spacemake, a unified computational framework for analyzing spatial transcriptomics data sets produced with Visium, Slide-seq, Seq-scope, or any other STS technology. Importantly, spacemake performs data processing and downstream analysis in the same way, resulting in uniform reports and quality metrics that are easier to compare and interpret across different technologies. This renders spacemake an excellent candidate for multimethod projects. Apart from the standardized processing of raw data, spacemake can perform additional analyses that we organize in different modules: integration of histological staining images, downsampling and saturation analysis, merging of biological replicates, spatial reconstruction of scRNA-seq data or merging of scRNA-seq and STS data sets by using novoSpaRc [11, 12], and analysis of long-read sequencing data for troubleshooting. Spacemake is written in snakemake [13] with a back-end logic written in Python. It provides an easy-to-use command-line interface, through which it can be configured and run using a handful of commands. It readily works with various types of array-based STS methods and allows diverse, user-definable processing modes. Spacemake is versatile and can be used as a new workflow or be readily integrated into existing pipelines. Finally, spacemake is open source and freely distributed through a GitHub repository.

## Findings

### Spacemake processes different input data in a single workflow

Spacemake can handle different sequencing-based spatial-transcriptomic data sets, such as those stemming from—but not limited to—Slide-seqV2, 10X Visium, or Seq-scope. In particular, it processes raw data (Illumina basecalls or fastq files) in identical fashion, regardless of the sequencing technology or the barcoding strategy of the spatial unit. As STS methods differ experimentally, we employ throughout the text the term *spatial unit* to describe the fundamental barcoded unit in space (e.g., beads, spots, or clusters).

**Figure 1:**
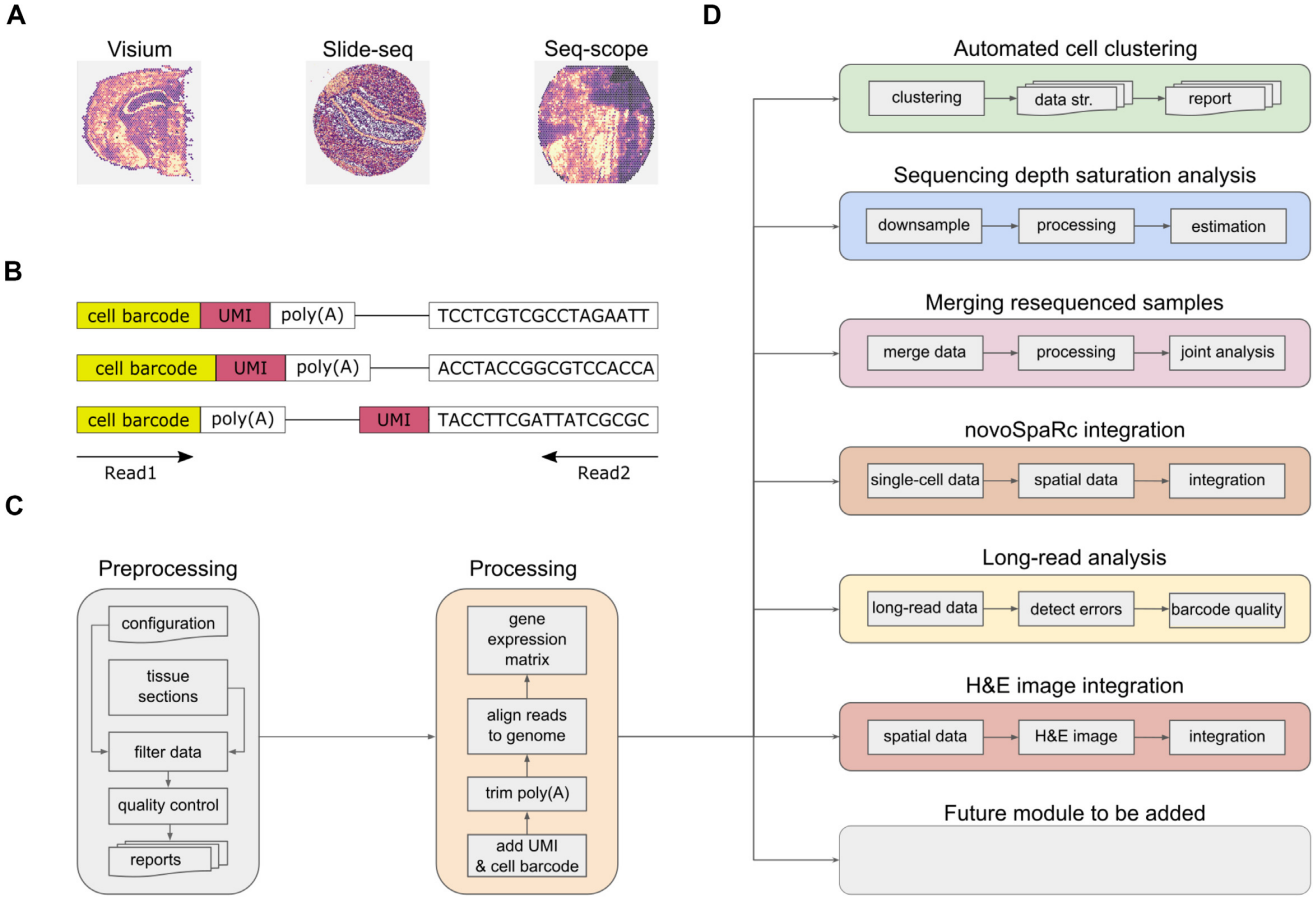
Overview of spacemake. (A) Spacemake can handle inputs from different spatial transcriptomics technologies. (B) Spacemake is able to handle any barcode strategy. Cell barcode and unique molecular identifier (UMI) lengths are variable, and their position can be on either read. (C) Preprocessing, qualuty control, and processing steps. Each sample is processed the same way, regardless of the input type. (D) Spacemake is modular and extendable. Each module is implemented with a separate set of rules and commands, and everything is assembled in a top-level Snakefile.

To allow for maximum flexibility, in spacemake, each sample is associated with a set of “sample variables,” namely, a “barcode-flavor,” at least one “run-mode,” a “puck,” and a “species” (Methods). The “barcode-flavor” describes the barcoding strategy, that is, how the spatial unit barcodes and the unique molecular identifiers (UMIs) should be extracted from Read1 and Read2. The “run-mode” parameter contains several variables that describe how the sample will be processed downstream and currently include poly(A) and adapter trimming, tissue detection, multimapping read counting, intronic read counting, barcode cleaning, meshgrid creation, and UMI cutoff (Methods). The “puck” parameter allows the user to specify the spatial dimensions and bead diameter size of the underlying STS assay. Lastly, “species” is a pair of a genome fasta file and an annotation file, from which spacemake will generate indices to be used later during mapping. After spacemake is configured and all parameters are set for all samples, it can be run, producing a unified and structured output for each sample (Fig. 1, Methods).

### Overview of the spacemake pipeline

Spacemake processes each sample starting from raw reads, which can be either Illumina basecalls or demultiplexed fastq files. In the first case, spacemake demultiplexes the data using Illumina's bcl2fastq2 tool [14]. Once raw fastq files have been created, a custom preprocessing script creates an unmapped BAM file: from each Read1, Read2 pair, a spatial unit barcode (or Cell Barcode, CB) and a UMI will be extracted and attached to the unmapped BAM file as CB and MI tags, respectively. For each sample, this extraction is based on the previously defined barcode-flavor. Read sequences in this unmapped BAM come from Read2 sequences. Next, using Dropseq-tools [15], adapters and 3′ poly(A) stretches are optionally trimmed from each read. Reads are then mapped with STAR [16] and by using samtools [17] to input the unmapped BAM. After mapping, each read that maps to a gene body will be assigned a gene annotation using the TagReadWithGeneFunction command of Dropseq-tools. If the run-mode has a multimapper counting turned on, spacemake will process the mapped BAM file line-by-line and out of all possible alignments keep at most one alignment per read, to be counted later. Specifically, a multimapper is kept only if there is exactly one alignment to a genic region and all others to intergenic regions. In this case, the intergenic alignments are discarded. If a read aligns to multiple genes, it is discarded. Finally, the digital gene expression (DGE) matrix is created using the DigitalExpression command of Dropseq-tools, with spatial unit barcodes used as a whitelist (Fig. 1). After the DGE matrix is created, each sample is automatically analyzed: data filtering and clustering is done with scanpy [18], and the resulting data are saved as an hdf5 file. At the last step, web-based reports are generated by using Rmarkdown [19] and knitr [20] (Methods).

### Spacemake produces unified quality control reports

**Figure 2:**
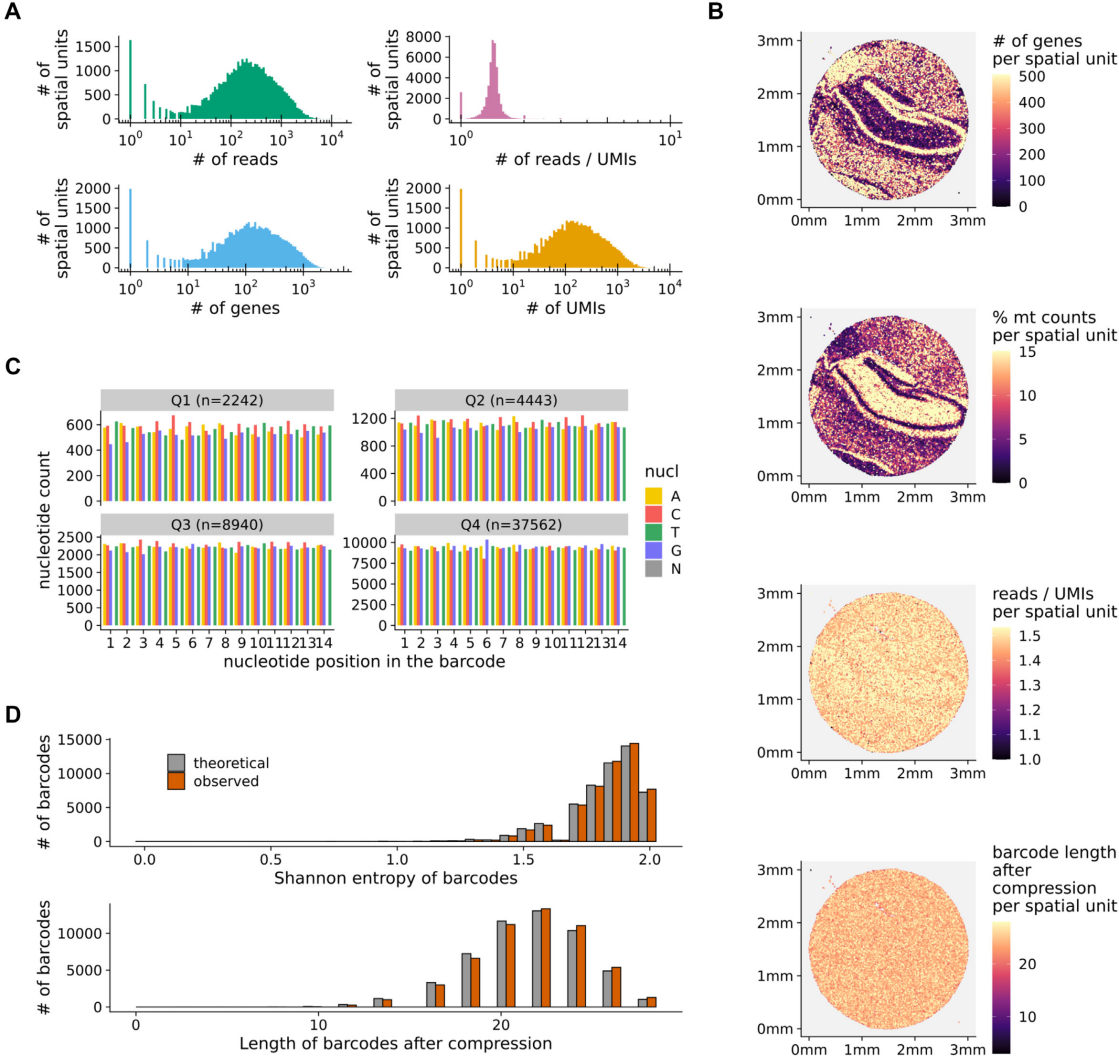
Spacemake produces uniform quality control reports. (A) Histograms showing the number of genes, reads, UMIs, and reads/UMIs ratios per spatial unit. (B) Quality control metrics plotted in tissue space. Top to bottom: number of genes, percentage of mitochondrial counts, reads/UMIs ratios, and barcode length after compression, all shown per spatial unit. (C) Nucleotide frequencies per barcode position and quantile (segregated by the number of reads). (D) Shannon entropy and string compression length of the sequenced barcodes versus the expected theoretical distributions.

Spacemake assesses the quality of each sample with multiple metrics. The commonly used FastQC [21] tool is first optionally called to assess sequencing library quality by flagging repetitive sequences, adapter content, GC bias, nucleotide composition, and basecall qualities, among others. Then, each sample is mapped to ribosomal RNA (rRNA) with bowtie2 [22] to assess the efficacy of poly(A) messenger RNA (mRNA) capture relative to abundant, contaminating rRNAs. After these quality control (QC) steps are run, a per-sample web-based QC report is generated (Fig. 2). In particular, the number of genes, reads, UMIs, and the reads/UMIs ratio are shown both as a histogram over all barcodes (Fig. 2A) and in tissue space (Fig. 2B). Randomness underlies the combinatorial complexity of the barcodes and is required for collision-free encoding spatial information. To assess the barcode randomness, the spacemake QC contains the following plots: a per-position nucleotide ratio, separated into quartiles by read counts (Fig. 2C, Methods), histograms of the Shannon entropy, and the string compression length of the observed barcode sequences against the expected theoretical distributions (Fig. 2C and D, Methods). Barcodes exhibiting unusual distributions in the per-position nucleotide ratio plot would imply artifacts in the sequencing data. Similarly, large deviations from the expected theoretical distributions of the Shannon entropy and the string compression would imply the existence of low-complexity barcodes in the data, so that troubleshooting would be required.

### Spacemake can readily aggregate spatial units

**Figure 3:**
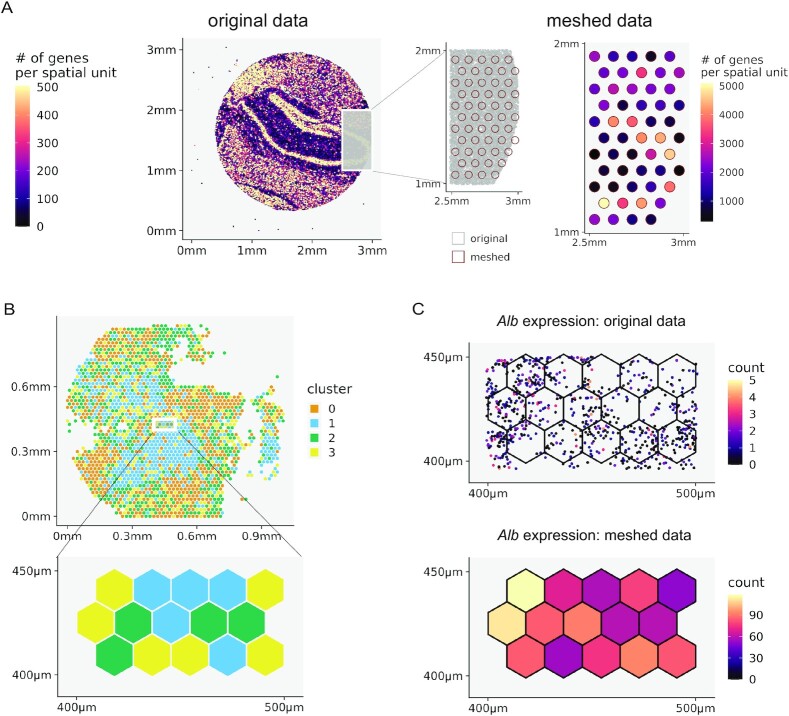
Spacemake seamlessly aggregates spatial units. (A) Spacemake can automatically create a Visium-style mesh grid (a 55-*μ*m diameter in a 100-*μ*m distance; also user defined) and further processes the data mapped on this mesh. (B) Running on subcellular resolution data sets, such as Seq-scope, spacemake utilizes mesh-creation to join subcellular diameter spots into a 10-*μ*m-side hexagonal mesh. After the hexagonal mesh is created, downstream analyses use it as input (e.g., for cell type identification). (C) The highest expressed gene for this adult mouse liver sample is shown. Top right: raw counts in the subcellular spots; bottom right: counts assigned to hexagonal mesh cells.

In some cases, it is useful to join nearby spatial units, effectively trading spatial resolution for statistical power by accumulating read counts (Fig. 3, Methods). This is particularly suitable for irregularly spaced data points, such as Slide-seq, or when the data stem from an STS assay with subcellular resolution and are hence sparse, such as Seq-scope [7]. In addition, this aggregation also facilitates the comparison of spatial technologies operating at different resolutions, for instance, Slide-seq and Visium.

In Seq-scope, for instance, ∼800,000 barcodes spread out on a 1 × 1 mm^2^ surface, so that the underlying diameter of each spatial unit is smaller than 1 *μ*m and contains a very low (not more than a few dozen) number of transcripts. To efficiently analyze such a sparse data set, it is practical to create a “meshed” grid (meshgrid) *in silico*, where the diameter of each newly created spatial unit is 10 *μ*m, the approximate size of a eukaryotic cell. Spacemake offers two types of meshgrids out of the box: (i) a Visium-style meshgrid, where circles with a certain diameter are placed at equal distances from each other in a hexagonal grid (Fig. 3A) and (ii) a hexagonal meshgrid, where equal hexagons are created on top of the whole data set, without holes in between (Fig. 3B). As the hexagonal meshgrid covers the entire area, no counts are discarded. For both meshgrids, spatial units falling into the same hexagon/circle are joined together and their gene expression counts are summed up (Fig. 2A, B).

### Downsampling analysis reveals library complexity and depth saturation

**Figure 4:**
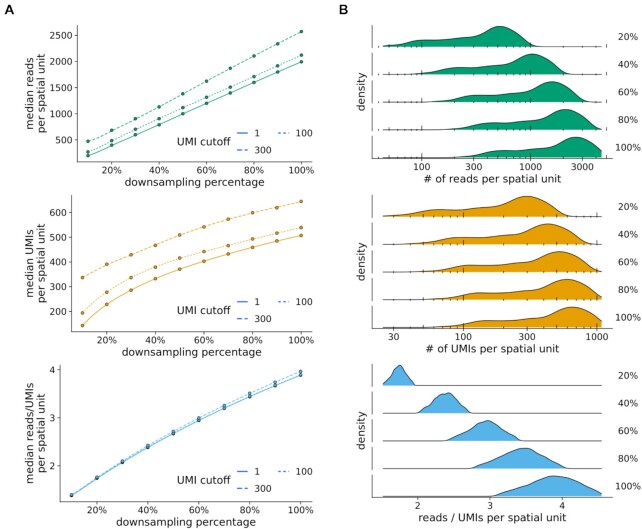
Spacemake can readily downsample the data to perform a saturation analysis. (A) Median number of reads, UMIs, and reads/UMIs ratios per spatial unit are plotted against the downsampling percentage. Saturation analysis reveals that this Slide-seq sample has not reached saturation yet, as the median UMIs curve has not reached a plateau. (B) Density plots of a Seq-scope downsampled data set.

To assess library complexity and if saturation has been reached in scRNA-seq or STS experiments, a downsampling analysis is employed to estimate whether resequencing would result in a higher number of molecular counts per spatial unit. In spacemake, saturation analysis is implemented as a separate module (Fig. 4, Methods). First, the final BAM file is subsampled to 10%, 20%, …, 90% of the total reads using sambamba [23], and for each ratio, a separate DGE matrix is generated. A saturation report is then compiled where median metrics are plotted as a function of the downsampling ratio (Fig. 4A). From the linearity of this curve, it can be deduced that saturation has not yet been reached for this Seq-scope sample, even at 10^9^ sequenced reads. In addition to plotting the median values, spacemake also reports histograms for each downsampling ratio per spatial unit, showing the global pattern rather than a single value per ratio (Fig. 4B, Supplementary Fig. S3E).

### Spacemake can readily merge technical replicates

Resequencing a library of sufficient complexity is a common practice to achieve higher molecular counts. A single experiment can result in several sequencing runs, with each of these replicates being technical, as the underlying library is the same. In these cases, the original and resequenced data sets have to be joined together, so that counts are quantified in the DGE matrix by properly removing duplicate reads. In spacemake, this process is implemented in the sample merging module, which inputs the two separate, already processed data sets and joins them. If a sequencing run was repeated for a sample, the user can add both samples separately to a spacemake project and later merge them using the spacemake command line (using the “spacemake projects merge_samples” command). After this step, a new, merged sample is created, and this sample will be processed in an identical manner downstream as the individual nonmerged samples. As a result, this module significantly reduces the hands-on computational analysis time when processing technical replicates.

### Spacemake offers a spatial reconstruction baseline of scRNA-seq data

**Figure 5:**
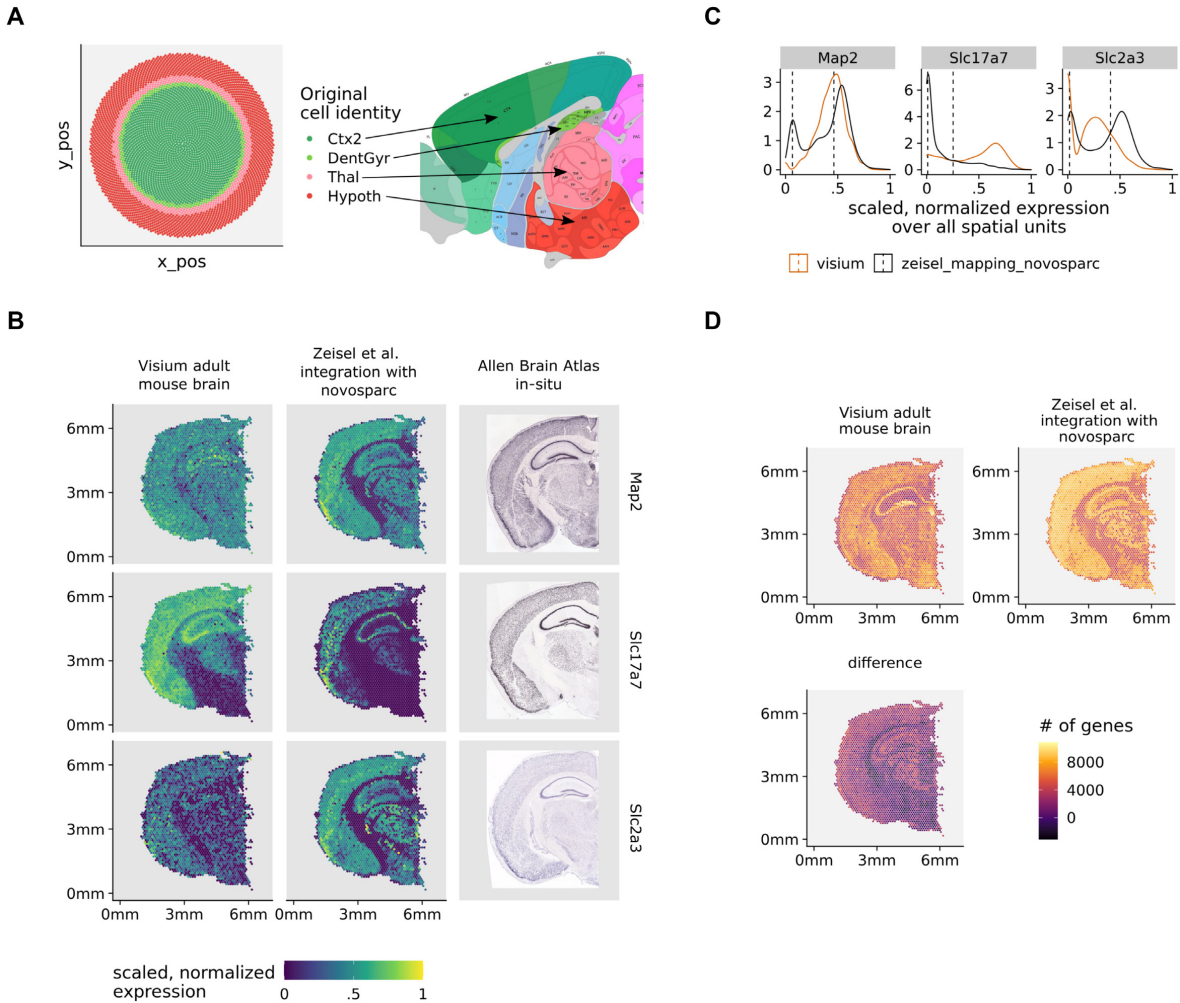
Spacemake can integrate scRNA-seq and spatial transcriptomics data sets. (A) Spatially mapping an adult mouse brain scRNA-seq data set with 30,000 cells onto an *in silico* created circular puck with 5,000 locations reveals cortical layers (left). Tissue labels used: Thal, CA1, Hypoth, Ctx2, DentGyr, SScortex. The identified clusters correspond to spatially distinct anatomical regions (right, adapted from Allen Reference Atlas—http://atlas.brain-map.org). (B) Expression of neuronal markers (*Map2*,*Slc2a3*,*Slc17a7*) in the original Visium data (right column), after novoSpaRc integration (middle column) and in *in-situ* images from the Allen Brain Atlas (left column). (C) The bimodal distributions of gene expression are shown together with the corresponding mean values. To arrive at the results of panel (D), the expression of each gene was modeled with a Gaussian mixture model with 2 components. For each spatial unit, only genes whose expression was in the upper mode were counted. (D) Integrating the single-cell and spatial transcriptomics data sets increases the number of genes quantified per spatial unit.

Although spacemake is primarily designed to process STS data sets, it can also efficiently process data produced by the more standardized and popular scRNA-seq technologies. By now, several pipelines exist for analyzing scRNA-seq data, for instance [24]. None of these, however, aims at incorporating a spatial reconstruction to the analysis. For this, spacemake utilizes novoSpaRc, a computational framework that reconstructs spatial information solely from scRNA-seq data based on the hypothesis that cells that are spatially neighboring also share similar transcriptional profiles [11, 12]. Although novoSpaRc greatly benefits when a reference atlas of gene expression is available, its *de novo* mode is powerful and can yield insights into substructures of complex tissues, such as liver lobules, the intestinal epithelium, or the kidney [11]. Spacemake employs novoSpaRc to yield a basic spatial reconstruction of scRNA-seq data that can serve as a baseline and be used to derive further insights (Fig. 5, Table 2, Methods). Applied to a data set of an adult mouse brain, for instance, spacemake recovers the basic structure representation of the mouse brain cortex when compared to the Allen Reference Atlas [25] (Fig. 5A).

**Table 2:** NovoSpaRc: modes offered by spacemake and their outcome based on data availability

**Data produced**	**Publicly available data**	**NovoSpaRc mode**	**Outcome**
–	scRNA-seq	*De novo*	Basic spatial reconstruction
–	scRNA-seq + spatial	With markers	Enhanced gene counts
scRNA-seq	–	*De novo*	Basic spatial reconstruction
scRNA-seq	Spatial	With markers	Enhanced gene counts
Spatial	scRNA-seq	With markers	Enhanced gene counts
Spatial + scRNA-seq	–	With markers	Enhanced gene counts

### Spacemake can integrate scRNA-seq data to a spatial transcriptomics data set

When both spatial and scRNA-seq data sets of the investigated tissue are available, spacemake leverages novoSpaRc to integrate them. For this, the spatial data set is regarded as a reference atlas and the scRNA-seq transcriptomes are mapped onto the locations of the spatial units. Importantly, spacemake is not restricted to a specific technology but can utilize any spatial data set as a reference atlas guiding the reconstruction. This becomes especially useful for widely studied or stereotypical tissues for which spatial data sets are already available, such as the adult mouse brain [26]. Mapping a publicly available scRNA-seq data set [26] onto an existing spatial data set [27], for instance, results in an enhanced number of genes per spatial unit (Fig. 5). Upon inspection of the spatial patterns of genes expressed across all neurons (*Map2*,*Slc2a3*,*Slc17a7* taken from http://mousebrain.org/ [26]), the expression profiles become more distinct and defined in space after novoSpaRc integration (Fig. 5B). Moreover, when compared to *in situ* images from the Allen Mouse Brain Atlas [28], the expression profiles with scRNA-seq data integration are more similar to the *in situ* hybridization (ISH) data than those without (Fig. 5B). To quantify the number of genes that are expressed in Visium spots after novoSpaRc integration, we modeled the expression of each gene by using a Gaussian mixture model with 2 components (Fig. 5C). Assuming that the lower (upper) mode of the bimodal distribution describes low-to-no (low-to-high) expression, we calculated for each spot the number of genes expressed and compared it to the original data (Fig. 5D).

### Spacemake can leverage long-reads to troubleshoot library construction

Generation of STS and scRNA-seq libraries can be challenging due to the low amounts of RNA that may be captured from some samples. Especially when protocols are customized to accommodate specific experimental goals and needs, we have found it helpful to investigate our sequencing libraries by long-read sequencing. To this end, spacemake features a module to automatically annotate tens of thousands of long reads against a user-provided reference of expected adapter sequences and other oligonucleotides such as primers used during library construction (Supplementary Fig. S2, Methods). The module then groups these annotations into recurring patterns of how these building blocks are arranged and provides an overview of the relative contributions of each class of such arrangements to the library. This allows the user to monitor cDNA integrity, for example, from 10X Chromium beads (Supplementary Fig. S2B, C), and enables to detect and subsequently mitigate potential primer and Template Switch Oligo (TSO) concatenations as described in [29].

### Spacemake offers flexible run-mode settings

**Figure 6:**
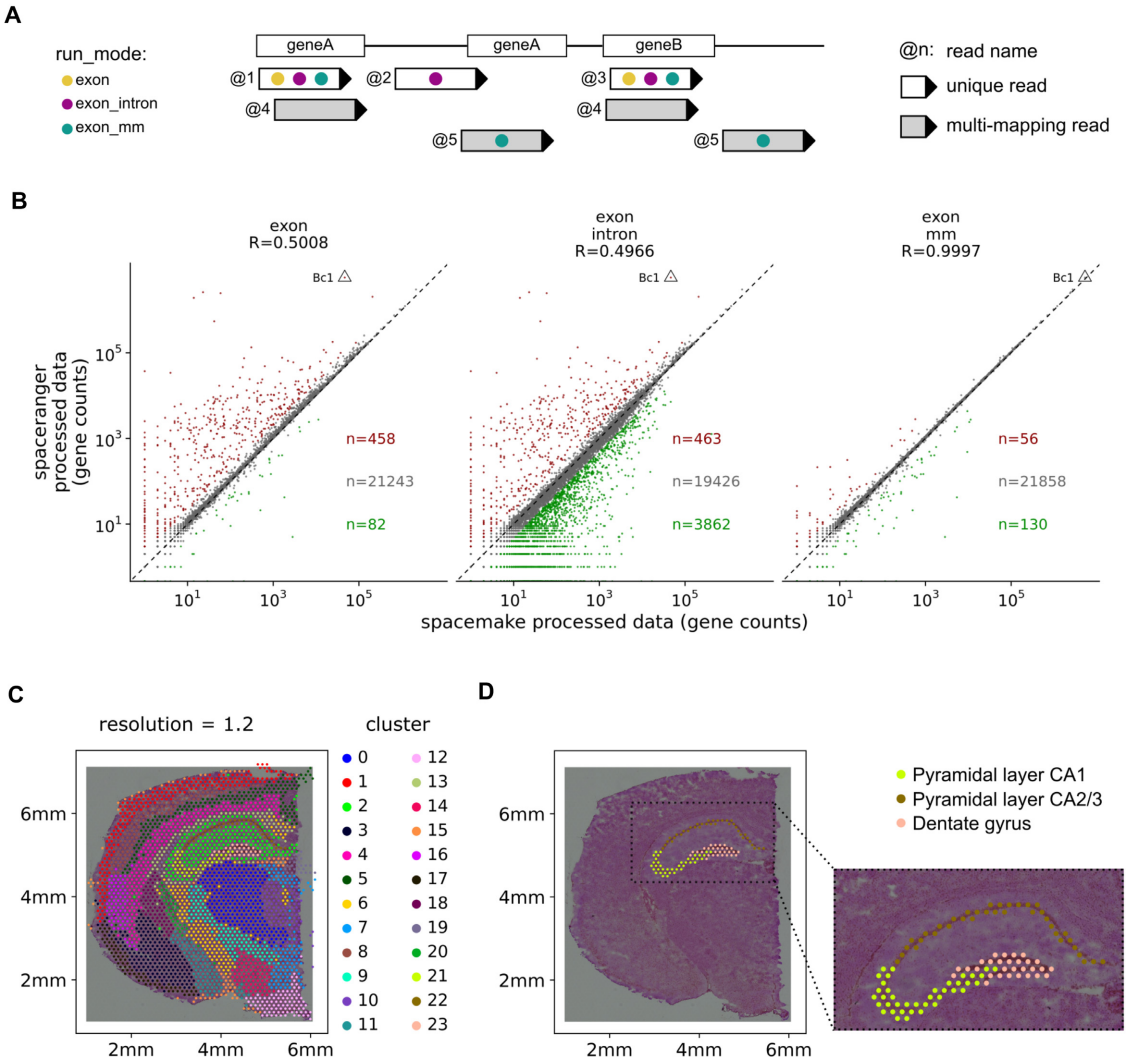
Spacemake offers several processing modes while producing a unified downstream output and can align spatial count data with H&E images. (A) Spacemake can be run using several user-defined settings. Gene quantification depends on the run-mode set to include reads mapping only on exons, on both exons and introns, on exons and intergenic regions, and whether the reads should be trimmed for poly(A)-tails and adapters. (B) Comparison of spacemake run-modes with spaceranger. Genes with twice higher counts are colored red (higher in spaceranger) or green (higher in spacemake); all other genes are colored gray. (C) Spacemake automatically performs clustering analysis of the data. At 1.2 resolution, clusters become distinct along defined structures in space, such as the cortical layers, CA2/CA3, CA1, and dentate gyrus. (D) Spacemake automatically aligns spatial transcriptomics data with H&E images. Here the pyramidal layers and the dentate gyrus, as taken from the Allen Brain Atlas, are shown to perfectly overlap with the corresponding clusters.

A major strength of spacemake are the user-defined run-mode settings. A run-mode is created with the configuration command and provides complete control over how samples using this run-mode should be processed downstream (Methods). Adapter- and poly(A)-trimming can be turned on or off, and multimapper and intronic-read counting rules can be set. As each of these settings produces different results (Fig. 6A, B), it is often beneficial to initially run the analysis with several run-modes in parallel and then identify robust and reproducible results.

To demonstrate spacemake's flexibility, we compared it against spaceranger on a publicly available adult mouse brain data set [27]. First we ran spacemake by using several run modes and then compared the results with that of spaceranger. We focused on 2 types of correlations between spaceranger and spacemake: (i) gene–gene expression Pearson correlation over all spatial units, treating the data as bulk (Fig. 6B, Supplementary Fig. S1C) and (ii) gene–gene expression Pearson correlation per spatial unit, in space (Supplementary Fig. S1A, B). We found the (i) correlations to be between 0.48 and 0.99 for all run-modes (Fig. 6B, Supplementary Fig. S1C) and the (ii) correlations to have a median value (per run-mode) between 0.5 and 1.0 (Supplementary Fig. S1A, B). As we observed that these correlation metrics mostly depend on the highest expressed gene in the data set (*Bc1*), we further counted the number of genes that are twice as abundant in spaceranger versus spacemake or vice versa (Fig. 6B, Supplementary Fig. S1C) and how large the difference of counts between the 2 processing methods is per gene (Supplementary Fig. S1D). Spacemake produced most similar results to spaceranger when poly(A) trimming is turned off; only exonic reads are counted and multimapping read counting is turned on (Fig. 6A ,B and Supplementary Fig. S2).

Building on top of the flexibility offered by run-modes, spacemake also allows to cluster the data using different parameters and saves all clustering results in the same automated analysis report (Fig. 6B). For the aforementioned data set, higher clustering resolution leads to more biologically meaningful regions identified: at resolution 1.2, for instance, the pyramidal layer of the hippocampus separates into CA1/2, CA3, and the dentate gyrus (Supplementary Fig. 4A).

### Spacemake provides automated downstream analysis

After processing is completed, spacemake performs a basic automated analysis of the data (Methods). For this, spacemake employs scanpy [18] and squidpy [18, 30]. More specifically, spacemake identifies cell types and their corresponding marker genes and plots them in an automatically generated report. If the user defines multiple UMI cutoffs for performing the downstream analysis, then multiple such reports are generated. For STS data sets in particular, spacemake uses squidpy to generate a cluster-to-cluster neighborhood enrichment heatmap (Supplementary Fig. S4B) to calculate co-occurrence of spatial units and predict ligand–receptor interactions between spatial units.

We benchmarked spacemake against the results obtained in a Slide-seqV2 data set [3]. For this, we first generated a raw fastq file from the slideseq-tools processed BAM file provided by the authors. Then, from the same file, we created a DGE matrix using Dropseq-tools [15]. Finally, using the raw fastq files as input, we ran spacemake and compared the results with the DGE matrix from the Slide-seqV2 BAM file. Spacemake achieves very high correlation with the Slide-seqV2 data, with most beads having a gene–gene correlation higher than 0.95 and the overall correlation being as high as 0.98 (Supplementary Fig. S4B). Spacemake automatic clustering identifies spatially informative clusters, such as the cortical region, mouse hippocampus pyramidal layer, dentate gyrus, and thalamic region, and the squidpy neighborhood enrichment analysis reveals spatial closeness of pyramidal-layer and cortical neurons (Supplementary Fig. S3C).

### Spacemake can automatically align and integrate hematoxylin and eosin data

Integrating imaging with spatial transcriptomics data can facilitate the investigation of complex tissues. Spacemake automatically aligns imaging data, such as hematoxylin and eosin (H&E) microscopy images, with count-based data (Fig. 6C, D, Supplementary Fig. S5, Methods). Upon aligning the Visium mouse brain data set [27] with the corresponding H&E .tiff image, we observed that the pyramidal layer of the hippocampus perfectly aligns with clusters from the automated clustering performed by spacemake (Fig. 6D). To further demonstrate spacemake's image alignment capability, we downloaded and processed 2 more public Visium data sets: a sagittal mouse brain section [31] and a coronal mouse kidney section [32] with their corresponding images. Spacemake successfully aligned both samples, illustrating that its underlying algorithm works well with Visium data (Supplementary Fig. S5C, Methods).

Contrary to Visium images, Seq-scope images do not possess a clear tissue boundary, thus hindering the alignment. Spacemake addresses this by first attempting to deduce the tissue boundaries from the H&E. In case this fails, the user can manually set the parameters to achieve a better match (Supplementary Fig. S5A, B). After identifying the tissue for the Seq-scope images, spacemake utilizes the same algorithm as for Visium to match the imaging and count data (Supplementary Fig. S5B, Methods).

### Spacemake is fast and scales with number of reads

Spacemake is fast, is scalable, and supports multithreaded processing. To benchmark spacemake, we processed the publicly available adult mouse 10X Visium data using both spaceranger and spacemake. We observed that when using 6 cores, spacemake is 1 hour faster than spaceranger while producing the same results (Supplementary Fig. S6A). Spacemake also scales well with the number of reads: for the Slide-seqV2 sample with 70 million reads, total run-time was just over 1 hour, while 1 billion Seq-scope reads took 18 hours to process (Supplementary Fig. S6A, B). Moreover, spacemake can run several samples in parallel. For a single sample, spacemake requires 4 cores minimum to run, so that with 8 or 12 cores, several samples can be processed together, thus starkly reducing the average running time per sample.

## Conclusions

As spatial sequencing technologies become increasingly available, the existence of robust, reproducible bioinformatics pipelines is of paramount importance. Here, we present spacemake, a comprehensive computational framework that efficiently analyzes spatial transcriptomics data sets stemming from different technologies. Spacemake is extendable, is scalable, and provides a complete solution from processing of raw data over several quality controls and automated reports all the way to advanced downstream analyses. Spacemake's core strength is the unified processing of different data types, rendering it highly suitable for projects that use multiple methods. Spacemake is open source, is freely available, and can be smoothly integrated with other packages that perform downstream analysis [30].

Spacemake is highly modular. It currently contains modules for downsampling and saturation analysis, sample merging, a baseline spatial reconstruction of scRNA-seq data sets, and analysis of long reads, and it can be readily extended to add more functionality. Moreover, spacemake is versatile enough and can be used to analyze not only spatial transcriptomics data sets but also scRNA-seq data. To demonstrate spacemake's capabilities, we have used it to process and analyze Slide-seqV2 and 10X Visium data sets, showing that spacemake accurately reproduces the processed data of the 2 technologies. We further illustrated how spacemake can integrate scRNA-seq and STS data sets by employing novoSpaRc.

It should be noted that currently, spacemake processes and analyzes sequencing data but not imaging data. Some spatial transcriptomics techniques, however, require registering the barcodes of the beads or spots in space by imaging. In a companion paper, some of us present a complete computational framework for efficiently handling such data sets, called Optocoder [33]. Spacemake can be readily integrated with Optocoder or similar methods.

Finally, it would be useful to extend spacemake to handle different types of data (e.g., protein expression or chromatin state). As novel techniques that provide diverse molecular readouts from the same cell are being constantly developed, it will be essential to possess a unified framework that can process the different data modalities. We plan to extend spacemake to accommodate such data sets in the future.

## Methods

### Run-mode settings

For each sample, 1 or multiple “run-modes” are defined to describe how spacemake should process it downstream. Each run-mode has a name and several parameters: automatic tissue detection (on/off), poly(A) and adapter trimming (on/off), intronic read counting (on/off), multimapping read counting (on/off), data meshing (on/off), number of expected barcodes, UMI cutoff, and DGE matrix cleaning (on/off). Each of these parameters is set through the command line. Currently, spacemake offers the following run-modes out of the box: scRNA_seq, visium, slide_seq, and seq_scope, with parameters corresponding to each technology.

### Data preprocessing and mapping

The publicly available data sets were obtained as described in the data availability section below. FastQC (v0.11.9) was used to assess sequencing quality, and a Python custom script was used to retrieve the cellular barcodes and UMIs for the different read structures (Visium: R1[1–16] for the spot barcode and R1[16–24] for the UMI and cellular barcodes; Seq-scope: R1[1–20] for the bead barcode and R2[1–9] for UMI; Slide-seq: R1[1–14] for bead barcode and R2[15–23] for UMI). During the barcode and UMI retrieval, an unmapped BAM was created where each R2 sequence was tagged with the correct cell-barcode and UMI.

#### Poly(A) and adapter trimming

If poly(A) and adapter trimming is switched on for the current run-mode, the 3′ ends of reads are trimmed for poly(A) and overlapping user-defined adapter stretches. This processing is performed with the functions TrimStartingSequence and PolyATrimmer of Drop-seq tools (v2.4.0) for poly(A) and adapter trimming, respectively.

#### Mapping and gene tagging

Alignment to the genome was performed with STAR (v2.7.9a) using the unmapped BAM as input and under the default parameters. The following genomes and annotation files were used: mm10 and M23 and were downloaded from Gencode. Gene tags were added with the function TagReadWithGeneFunction of Dropseq-tools.

#### Multimapping read counting

Multimapping reads were counted using a custom Python script that parsed the read-name sorted (STAR default output) final BAM line-by-line. For each read name, maximally 1 read was kept. If a read mapped to several genomic locations—but only 1 exonic region—this exonic-mapping read was kept and the rest were discarded. If a read mapped to several exonic locations, it was removed altogether. During parsing, each kept read was flagged as primary, and the parsed output (now containing at most 1 read for each multimapper) was piped into the DigitalExpression of Dropseq-tools, which was run with a MAPQ = 0 filter, to ensure multimapper inclusion.

#### DGE creation

Once the aforementioned steps are run, the DGE matrix is generated. If the provided data set contains a list of spatial barcodes, it is used as a “whitelist.” Otherwise, snakemake uses the n_beads parameter of the current run-mode to select the top n_beads number of barcodes with the highest read count using the BamTagHistogram function of Dropseq-tools. Finally, the DGE matrix is generated using either the “whitelist” of spatial barcodes or the top n_beads barcodes.

#### DGE barcode cleaning

For a user-defined set of primers, spacemake can optionally discard barcodes that overlap with any of these primers. This is controlled by the clean_dge parameter of a run-mode. When set to true, the following barcodes are removed: (i) barcodes that have at least 4 nt overlap with any of the primers in the 3′ end and (ii) barcodes that have an at least 7 nt overlap with any of the primers, anywhere in the barcode itself. If selected, this step is run before generating the DGE matrix.

### Tissue detection

For the samples in which tissue detection was turned on, spacemake performed it as follows: first, spatial units with UMIs above a certain threshold (provided by the user) were treated as “under-the-tissue” spatial units. Then, for each tissue spatial unit, its neighboring spatial units were computed. For 10X Visium, that is straightforward, as the data points lie within a hexagonal grid. For irregular grids such as Slide-seq data sets, we created a meshgrid and then quantified the spatial unit neighborhoods. This resulted in the generation of contiguous areas. Spatial units lying within the largest contiguous area were then considered to be under the tissue.

### Automated downstream analysis

For downstream analysis, the text-based DGE matrix was first parsed line-by-line using a custom Python script to create a sparse matrix (Compressed Sparse Column), cast as an AnnData object, and finally saved in h5 format to ensure minimal space. Then, the standard scanpy single-cell workflow followed with default parameters. We selected the top 2,000 highly variable genes and 40 principal components to use for clustering using the leiden algorithm [34] and lower-dimensional representation with UMAP [35]. Each sample was clustered using the scanpy.tl.leiden functions and for several resolution values. Cell-type markers were identified with the rank_genes_groups function. For STS data sets, squidpy was used by running the built-in squidpy.gr.spatial_neighbors function. Spatial co-occurrence was computed with squidpy.gr.co_occurrence and the ligand–receptor analysis with squidpy.gr.ligrec.

### Meshgrid creation

We created the mesh grids *in silico* using the numpy.mesh function. For both grids (Visium style and hexagonal), a rectangular grid was first created with spot_distance_um (spacemake parameter—user definable) horizontal distances and sqrt(3) * spot_distance_um vertical distances. This mesh was then duplicated and spatially translated, so that the result of the 2 meshes was a mesh where the distance between any 2 neighboring points was exactly spot_distance_um. For the Visium-style mesh, we joined beads that fall into any circle (with mesh points as circle centers) with a diameter of diameter spot_diameter_um. For the hexagonal mesh, we calculated the distance between each spatial unit in the data and the mesh points, and for each spatial unit, we selected exactly one mesh point, the one with the minimum value.

### Downsampling analysis

Downsampling analysis was done by first splitting the final BAM file into different percentages with sambamba (v0.6.8). Then, the downsampled BAM files were fed into the same processing pipeline described above for further analysis.

### Spatial reconstruction with novoSpaRc

The *de novo* spatial reconstruction of the adult mouse brain scRNA-seq data was done with novoSpaRc (v0.4.3) and by using the default parameters and a circular disk as a target space. The top 100 highly variable genes were selected for the reconstruction. For the spatial reconstruction with markers, the corresponding Visium data set was used to first create a reference atlas. The top 200 highly variable genes were first obtained (both from Visium and single-cell data sets), and 195 of them remained after intersecting them. Reconstruction was done with novoSpaRc and with parameter alpha = 0.5. For the *de novo* reconstruction, we used single-cell data from 6 areas: Thal, CA1, Hypoth, Ctx2, DentGyr, and SScortex. To assign each of the 5,000 positions to one of the annotated areas, for each position, the area having the highest median value for that position was picked. In this way, each position was assigned only to 1 area. Out of the 6 original areas, 4 were assigned to at least 1 position (Ctx2, DentGyr, Thal, Hypoth) and 2 did not have the highest median probability for any position (SScortex, CA1).

### Long-read analysis

The complementary DNA (cDNA) molecules should contain specific oligonucleotide building blocks in the right places, in addition to mRNA sequence and (parts of) the original poly(A) tail. Spacemake first aligns a catalog of such building blocks (SMART primer handles, poly(T), Template Switch Oligo, Illumina sequencing adapters, etc.) via local Smith & Waterman to each read. These alignments are then analyzed jointly for each long read and condensed into “signatures” that identify the presence/absence and relative ordering of each building block. Finally, the observed signatures are counted, compared systematically against the expected signature (for example: P5, bead_start, poly(T), N70X for a Dropseq bead-derived Illumina library) and the following diagnostic plots are generated: graphical breakdown of the library by signatures, zoom-in on bead-related features, mismatch and deletion analysis, and histograms of start/end positions for each part of the expected signature. We acquired the publicly available data as described below, and every 250th read was selected and analyzed with the spacemake.longread module using the “chromium” long-read signature.

### QC reports

QC plots were created with custom R scripts based on the ggplot2 package (v3.3.5). The automatically generated QC sheets were created with a custom Rmarkdown script, which takes the downstream processed and analyzed data files and creates the .html QC report. The Shannon entropy for each spatial unit barcode BC was calculated using the following formula:

$$
\begin{equation*}
{H_{BC\,\,}} = \,\, - \mathop \sum \limits_{n \in BC}^{} f\left( {n,\,\,BC} \right)\,\,*lo{g_2}\left( {f\left( {n,\,\,BC} \right)} \right),\,\,
\end{equation*}
$$

where $f( {n,\,\,BC} )$ is the relative frequency of a nucleotide *n* in barcode $BC$. The length of string compression for a spatial unit barcode was calculated the following way: first the barcode BC was compressed (such that AAACATTA becomes 3A1C1A2T1A) and then the character length of this compression representation was returned. The observed values were compared against theoretical values as follows: random barcodes were first generated for each sample, and their Shannon entropy and string compression were then computed. The number of random barcodes generated was always the same as the number of real barcodes, for all samples.

#### External DGE processing

Spacemake offers the possibility to process external count data. In this case, instead of starting from the raw data, the sample is processed downstream from the DGE matrix creation. Spacemake will perform the automated analysis and clustering and generate the corresponding reports.

### Alignment of H&E images with count data

#### Generating binary images from count data

To align H&E images with spatial count data, spacemake first generates gray-scale images from the count data themselves. For Visium, a 1,000 × 1,000 pixel image was generated (corresponding to a 6.5 × 6.5 mm square glass) based on the beads that were previously identified by spacemake as under the tissue (Methods). Each bead was then drawn using the openCV circle() function—according to Visium coordinates—with a 100-micron distance from each other, with each bead having 55-micron diameter. Each circle was drawn black and the rest of the image was white.

For the Seq-scope samples, a pixel image was first generated from the actual count data. Raw counts were scaled to have a maximum value of 255, so that the image could be stored as an 8-bit grayscale. Then, pixels were aggregated so that the image had a 1,000 × 1,000 pixel dimension. Next, a binary filter was run between 190 and 200 to generate the binary image from grayscale, hence resulting in the final binary count image.

#### Generating binary images from H&E

H&E images were first loaded using the openCV imread() function. Next, grayscale images were generated using the openCV cvtColor() function. Then a binary image was generated using the openCV threshold() function, using automatic thresholding.

#### Matching binary H&E image and binary count image

To align the H&E image and the count image, we used the openCV matchTemplate() function. This function, given a reference image (in our case the binary H&E), finds the position at which a template image (in our case, the binary count image) has the highest correlation with the reference image. Our algorithm will first scale down the binary count image to be one-third of the size of the H&E image, and then gradually the zooming ratio will be increased and the highest correlation will be picked. The zooming itself scales y and x independently, thus ensuring that if the x–y ratios are not matching between the template and the reference, the match would be still found. Finally, at the last step, the highest correlation is picked, and the resulting H&E is imaged and saved. Then, this image can be imported using spacemake's attach_he_image function.

## Code Availability and Requirements

Spacemake is freely available and can be found on GitHub [36].

Operating system: Unix

Programming language: Python, R

Requirements: Python 3.6 or higher, R 4.0 or higher

License: GPLv2

RRID: SCR_022 207

biotools ID: spacemake

## Data Availability

### Spacemake

Supporting data and an archival copy of the code are also available via the GigaScience database GigaDB [37].

### Slide-SeqV2

The Slide-seqV2 adult mouse brain data set was downloaded from [38] (Puck_200 115_08).

### Visium

The 10X Visium data sets were downloaded from [27] (coronal mouse brain), [32] (coronal mouse kidney), and [31] (sagittal mouse brain).

For each sample, we downloaded the original .fastq.gz raw files and processed them with spacemake. For the H&E integration, we downloaded the original high-resolution .tif images and resized them to 10% using ImageMagick [39] before integration.

### Seq-scope

Seq-scope data were downloaded from [40]. For the analysis shown in this article, the data set from healthy mouse liver with accession ID SRR14082759 was used. We used tile numbers 2105, 2106, and 2107 and extracted the bead barcodes, and their positions were from raw fastq files found in [41], with the help of Seq-scope's own script available in [42].

For the H&E integration, we downloaded the original .jpg files from [43]. We aligned count data from tile 2105 with used wt_4X_2.jpg and for count data from tiles 2106 and 2017 with wt_4X_1.jpg.

### Single-cell data

For the single-cell and novoSpaRc mapping, we used publicly available adult mouse brain data from [26], available in [44]. We only used tissue labels comparable with the spatial Visium sample, namely, Thal, CA1, Hypoth, Ctx2, DentGyr, and SScortex. We processed the data using spacemake and by treating them as an external DGE matrix.

### Long-read data

For long-read sequencing data, we used a subset of reads from SRR9008425 and SRR9008429, which were Nanopore sequenced cDNA sequences derived from 10X Chromium beads from [45].

## Additional Files

**Supplementary Fig. S1**. Spacemake offers customizable run-mode settings and correlates well with spaceranger.

**Supplementary Fig. S2**. Nanopore long-read analysis.

**Supplementary Fig. S3**. Spacemake efficiently processes Slide-seqV2 data.

**Supplementary Fig. S4**. Using a higher-resolution parameter during clustering leads to more defined clusters in the physical space.

**Supplementary Fig. S5**. Spacemake integrates and aligns spatial count data with H&E images.

**Supplementary Fig. S6**. Spacemake is fast, scales well, and can simultaneously process multiple samples.

giac064_GIGA-D-21-00370_Original_Submission

giac064_GIGA-D-21-00370_Revision_1

giac064_GIGA-D-21-00370_Revision_2

giac064_Response_to_Reviewer_Comments_Original_Submission

giac064_Response_to_Reviewer_Comments_Revision_1

giac064_Reviewer_1_Report_Original_Submission Qianqian Song -- 11/29/2021 Reviewed

giac064_Reviewer_1_Report_Revision_1 Qianqian Song -- 3/21/2022 Reviewed

giac064_Reviewer_2_Report_Original_Submission Ruben Dries, Ph.D. -- 12/26/2021 Reviewed

giac064_Reviewer_2_Report_Revision_1 Ruben Dries, Ph.D. -- 3/25/2022 Reviewed

giac064_Supplemental_Figures

## Abbreviations

STS: Spatial Transcriptomics Sequencing

DGE: Digital Expression Matrix

CB: Cell Barcode

UMI/MI: Unique Molecular Identifier

TSO: Template Switch Oligo

QC: Quality Control

H&E: Hematoxylin and eosin

## Competing Interests

The authors declare no competing interests.

## Author Contributions

N.K. conceived, designed, and implemented the initial version of the pipeline. T.R.S.-T. implemented the pipeline in snakemake. T.R.S.-T. and M.J. developed the pipeline. T.R.S.-T designed and implemented all modules, except for the long-read analysis module that was designed and implemented by M.J. T.R.S.-T. performed all computational and data analyses except for the long-read analysis, which was performed by M.J. N.K. and N.R. supervised the study. All authors wrote the manuscript.
