# Supplementary material for: Spacemake: processing and analysis of large-scale spatial transcriptomics data
Source: Gigascience. 2022 Jul 19;11:giac064. doi: 10.1093/gigascience/giac064 (PMC9295369; doi:10.1093/gigascience/giac064)
Supplement: giac064_GIGA-D-21-00370_Revision_1 [file giac064_giga-d-21-00370_revision_1.pdf]

GigaScience

Spacemake: processing and analysis of large-scale spatial transcriptomics data

--Manuscript Draft--

**Manuscript Number:**	GIGA-D-21-00370R1
**Full Title:**	Spacemake: processing and analysis of large-scale spatial transcriptomics data
**Article Type:**	Technical Note
**Funding Information:**	Deutsche Forschungsgemeinschaft (RA 838/5-1) — Dr Nikolaus Rajewsky<br>Deutsche Forschungsgemeinschaft (KA 5006/1-1) — Dr Nikos Karaiskos<br>H2020 Marie Skłodowska-Curie Actions (721890) — Dr Nikolaus Rajewsky<br>Deutsche Forschungsgemeinschaft (Excellence Cluster 2049/Neurocure) — Dr Nikolaus Rajewsky<br>Deutsches Zentrum für Herz-Kreislaufforschung — Mr Tamas Ryszard Sztanka-Toth
**Abstract:**	Spatial sequencing methods increasingly gain popularity within RNA biology studies. State-of-the-art techniques quantify mRNA expression levels from tissue sections and at the same time register information about the original locations of the molecules in the tissue. The resulting datasets are processed and analyzed by accompanying software which, however, is incompatible across inputs from different technologies. Here, we present spacemake, a modular, robust and scalable spatial transcriptomics pipeline built in Snakemake and Python. Spacemake is designed to handle all major spatial transcriptomics datasets and can be readily configured for other technologies. It can process and analyze several samples in parallel, even if they stem from different experimental methods. Spacemake's unified framework enables reproducible data processing from raw sequencing data to automatically generated downstream analysis reports. Spacemake is built with a modular design and offers additional functionality such as sample merging, saturation analysis and analysis of long-reads as separate modules. Moreover, spacemake employs novoSpaRc to integrate spatial and single-cell transcriptomics data, resulting in increased gene counts for the spatial dataset. Spacemake is open-source, extendable and can be seamlessly integrated with existing computational workflows.
**Corresponding Author:**	Nikos Karaiskos<br>Max Delbrück Centrum für Molekulare Medizin Berlin Buch<br>Berlin, GERMANY
**Corresponding Author Secondary Information:**	
**Corresponding Author's Institution:**	Max Delbrück Centrum für Molekulare Medizin Berlin Buch
**Corresponding Author's Secondary Institution:**	
**First Author:**	Tamas Ryszard Sztanka-Toth
**First Author Secondary Information:**	
**Order of Authors:**	Tamas Ryszard Sztanka-Toth<br>Marvin Jens<br>Nikos Karaiskos<br>Nikolaus Rajewsky
**Order of Authors Secondary Information:**	
**Response to Reviewers:**	Dear Editor,<br><br>We would like to thank both reviewers for their constructive comments. We have now enhanced spacemake and revised our manuscript accordingly, addressing all raised

Powered by Editorial Manager® and ProduXion Manager® from Aries Systems Corporation

points.

Importantly, we have introduced a new module in spacemake that integrates imaging and spatial transcriptomics data, for instance images stemming from histological stainings, such as H&E. As H&E stains are common practice in several spatial transcriptomics technologies, we believe that iterating the two data modalities can provide significant insights. We demonstrate spacemake's integration of H&E images in several Visium and Seq-scope samples (Fig 6D and Sup Fig 5, Methods). Further details on the usage of the new module are found in the online documentation of spacemake.

Regarding the installation of spacemake, we have now tested it in several computing environments running UNIX (Ubuntu 18.04, Ubuntu 20.04, CentOS) and could not reproduce the installation problems that Reviewer 2 mentioned. We note that, a critical step in the installation of spacemake, is the existence of a new Conda environment. We have now clarified this further in the online documentation of our tool. The message mentioned by the reviewer is a warning message shown by the pysam library, which arises from backward compatibility issues with htslib (more info below). In fact, this warning message is shown during every spacemake run, even when the pipeline finishes without any errors. As it is difficult to deduce the actual cause of the installation failing from this one warning message alone, we invite the reviewer to send us their snakemake log files, so that we can further investigate the issue.

Our point-by-point responses to the reviewers' comments follows below.

We believe that we have now addressed all comments and have substantially improved both spacemake and our manuscript.

On behalf of the authors,

Dr. Nikos Karaiskos

Reviewer #1:
This manuscript proposed a python-based framework named spacemake, to process and analyze spatial transcriptomics datasets. It offers functionalities including sample merging, saturation
analysis and analysis of long-reads as separate modules, etc. Overall, this tool holds promises for spatial analysis, though this manuscript lacks details and explanations of methods and results. Specifically, I have some concerns regarding this manuscript.

1) As shown in table 1, it is noticeable that spacemake doesn't include H&E integration, which is kind of necessary in spatial data. I would recommend the authors at least discuss the potential functionality in including H&E images.

A: We have now included H&E integration in spacemake. An additional module offers automatic integration of imaging and spatial data. For the cases where the H&E image is of low quality, the user can perform a manual integration based on ImageJ. To integrate with ImageJ, the user has first to generate a grayscale image from count data using spacemake, and then align this image with H&E using ImageJ. This is done by first selecting a few (at least 4) corresponding points between the two images, and then transforming the landmark correspondences. Finally, the resulting aligned H&E can be readily attached to spacemake processed datasets.

2) From the legend of Fig 2B, I didn't find the plot with Shannon entropy, please double check.

A: We have corrected the legend of Fig 2B (previously Fig 1B) so that it corresponds to the figure panel shown.

3) I don't understand the meaning of fig 2D. The authors should explain how they calculate the Shannon entropy and string compression length of the sequenced barcodes, as well as how they define the expected theoretical distributions. More details are needed here. Though the authors mentioned related information/details

Powered by Editorial Manager® and ProduXion Manager® from Aries Systems Corporation

would be in methods (last line in QC section), I didn't find any in methods.

A: The methods for calculating the Shannon entropy and the string compression length were described in the Methods, under the QC reports section. We have now added a sentence in the corresponding passage of the main text describing the interpretation of the plots.

4) In Fig 4 A, the authors show the mapped scRNA-seq of mouse cortical layers. I think a complement spatial plot with annotations is necessary, as there is a gap between Fig 4A and Fig 4B.

A: We have now complemented Fig 5A (previously Fig 4A) with the corresponding sagittal section from a mouse brain to demonstrate the annotations, and recolored the figure so that the identified clusters and anatomy matches.

5) Fig 5C lack the annotations of different colors.

A: We now show the figure legend for Fig 6C (previously Fig 5C), as well as for the newly introduced Sup Fig 4

6) In page 16, the authors cited a manuscript in preparation, which is not good. I suggest remove the citation.

A: We have now adapted the reference and properly cite the manuscript that is publicly available as a bioRxiv pre-print.

7) Supplementary Fig 1 would be better if put as fig 1, thus it would show the overall flow & functionality of spacemake.

A: We agree with the reviewer's point and have now adapted Sup Fig 1 and have put it as Fig 1.

8) Based on Supplementary Fig 1, the authors should add a section illustrating how they annotate the spatial data and the involved gene markers.

A: We have now modified the header of the respective module in Fig 1 (previously Sup Fig 1) to illustrate that spacemake performs automatic cell clustering analysis and not cell type identification with associated gene markers. The latter would require the existence and the leveraging of a comprehensive cell type dataset, which falls beyond the scope of the current manuscript.

9) The paragraph "Spacemake can readily merge resequenced samples" lacks detailed explanation and results.

A: One of spacemake's functionalities is the handling of technical replicates, that is, the cases where a library is re-sequenced. In this case, raw data is spread across several files and the user would have to merge them themselves. To facilitate data analysis, we have included a module into spacemake that merges replicates which were already added to the spacemake projects. As this module is purely technical we omit showing figures in the manuscript. Motivated by the reviewer's comment, we have now updated the corresponding section in the manuscript and further included an explanation in the docs.

10) Though spackemake claims it is fast in processing data, well, Supplementary Fig 5 doesn't fully support that. Meanwhile, the authors should explain what the different colors represent.

A: In Sup Fig 6 (previously Sup Fig 5) it is shown that spacemake processes data at least as fast as spaceranger, if not faster (panel A, red and brown bars). Panel B demonstrates that spacemake scales well with a higher number of reads in the data, as the times in the panel are normalized by the reads number. The different colors correspond to the colors shown in the legend. As spaceranger is not modular, it is not possible to extract from its pipeline the running times for the individual processing steps. We have now adapted the figure to clarify the different colors, as well as

Powered by Editorial Manager® and ProduXion Manager® from Aries Systems Corporation

expanded the figure legend with these details.

11) In Supplementary Fig 2, the authors show very high correlation between spacemake and spaceranger, especially the exon intron and exon sub-figures. It looks like the correlations is close to 1. I suggest the authors double check the results and give explanations on their correlation analysis.

A: We thank the reviewer for insisting on this point. We have now double checked and verified the results of our correlation analysis. There is indeed a high concordance between spacemake and spaceranger when multi-mapper reads are included in the quantification, as the vast majority of genes (>21,800) fall along the diagonal. When multi-mapper reads are excluded, the correlations drop to R=0.4966 and R=0.7312, due to a large number of genes (~4,200 and ~4,000) that fall out of the diagonal. Overall, however, the correlations are largely driven by the most highly abundant gene (Bc1). Therefore, we have complemented our analysis by showing boxplots of the difference in normalized gene expression between spacemake and spaceranger (Sup. Fig. 1D).

Reviewer #2
In this article, the authors created a modular and scalable pipeline to process raw sequencing data from spatially resolved transcriptomic technologies. In contrast to other popular genomics technologies, such as (single-cell) RNA sequencing, there are virtually no existing public tools that allow users to quickly and efficiently process the raw spatial transcriptomic sequencing data that are generated through Illumina sequencing. This is largely due to the fact that each spatial transcriptomic workflow creates its own unique spatially barcoded reads and thus typically requires technology-specific tools or scripts to extract both the barcode and gene expression information. Here the authors created Spacemake which consists of multiple modules that are tied together using the popular workflow management system Snakemake. The innovative part of Spacemake comes from the creation of specific 'sample variables', such as the barcode-flavor, run-mode and puck, which
allows them to create a flexible pipeline that in theory can be adapted to any type of spatial array-based sequencing technology. The authors use well-established tools for downstream quality control and data processing and provide useful additional modules to assess or improve spatial data quality. Finally, Spacemake is also directly linked to Squidpy for downstream analysis and creates a web-based report, which could certainly help to lower initial spatial data analysis barriers.

Overall, the presentation of the tool and the methods used in the pipeline as described in their contents are comprehensive and the user manual is easy to understand. We appreciate the efforts to provide this tool to the spatial transcriptomics community and to make it open-source and flexible. However, we do have some suggestions and concerns regarding the manuscript and/or use of this tool.

Major comments:
1. We managed to install the spacemake software on the linux based server but failed to install it on a MacOS machine due to the compatibility issue with bcl2fastq2. Unfortunately, we also ran into an issue on our linux server, which happened during one of the reading steps from "/dev/stdin" in the middle of the spacemake workflow. More specifically we encountered the following error:

Job error: Job 7, TagReadWithGeneFunction
Error message: [E::idx_find_and_load] Could not retrieve index file for '/dev/stdin'

Even with the help of our IT team we were unable to resolve this issue. To help troubleshoot it might be helpful if the authors can provide exact commands for the examples provided in the manuscript and show what should be expected output of each job in the snakemake pipeline. As a result we were unable to re-run any of the provided examples, which severely limited our reviewing options.

A: The error mentioned by the reviewer should not pose an issue, and is expected. It is produced by pysam as a warning when there is no index file available for a BAM file, even though it is not required (spacemake requires a pysam version of at least

0.16.0.1). A thread on github explains this: https://github.com/pysam-developers/pysam/issues/939. We believe that if spacemake fails to install, it is most likely due to inconsistent packages installed. We have successfully run and installed spacemake on three separate platforms (Ubuntu 18.04, Ubuntu 20.04 and CentOS 7.9.2009) without any problems. We highly recommend following the installation instructions provided here: https://spacemake.readthedocs.io/en/latest/install.html. Briefly: (1) install mamba, (2) create a fresh conda environment using mamba and the provided environment.yaml, (3) run `pip install spacemake`. Should the reviewer encounter additional errors during installation, we would be pleased to assist troubleshooting via log files, or by directly reporting the error through our github page.

2. A major drawback of Spacemake is that it currently does not offer solutions for the integration of imaging information, which is typically an essential step in any spatial sequencing workflow. The authors do note this shortcoming in their discussion and as a potential solution they argue that Spacemake can be used with another tool called Optocoder, which is currently being developed in their lab. However no information can be found anywhere. There is no biorxiv or github page available based on our search results and as such we were unable to test or assess this solution. At minimum the authors should provide general guidelines on how users could potentially integrate images together with the created spatial downstream results.

A: We agree with the reviewer that aligning and integrating imaging data is important when it comes to spatial transcriptomics. Therefore, we have extended spacemake with an additional module that offers an automatic image alignment algorithm. We demonstrate its usage by automatically aligning several Visium and Seq-scope datasets with their corresponding H&E images (Fig. 6D, Sup. Fig. 5, Methods)

Minor comments:
1. The figure labels and legends are not always clear. More specifically it's sometimes hard to figure out which samples are being used for each figure or panel. This could be simply resolved by writing more informative legends that specifically state which sample was used to create each figure panel. According to the text Seq-Scope was used to generate figure 3, however in the legend of figure 3 it says Slide-seq …

A: We have now corrected the figure legend of Fig. 4 (previously Fig. 3). Also, we have expanded and rewritten the legends of all figures that needed further clarification.

2. Overall, the figures are pretty and informative, however I would suggest starting with a general overview figure that highlights the spacemake pipeline and it's innovative framework. Given the goal and content of the manuscript this seems to be appropriate as a main figure.

A: We agree with the reviewer and have now moved the former Sup. Fig. 1 as Fig. 1.

3. In order to initialize a spacemake project, the dropseq tools that are required by Spacemake lack any introduction. Please provide a brief introduction and a link to the associated github page to improve this step.

A: We have now added an explanation about dropseq-tools to our docs.

4. In order to configure the spacemake project by adding a sample species, the pipeline does not allow compressed versions of genome files. This could be simply fixed and allows the user to directly link to their, typically compressed, genome files.

A: Spacemake now allows the addition of compressed genome and annotation files.

5. More information is needed about the R1 R2 arguments in the add sample function. For example, SeqScope has two separate libraries to get sequenced. Where each round of libraries should be loaded is not immediately clear from the tutorial the authors provided.

A: In the manuscript we have used a bash script written for Seq-scope (Cho et al. 2021) to generate the coordinate file from the FASTQ flowcell library. In spacemake, R1 and R2 refer to the tissue library, and that holds true also for Seq-scope. We have

Powered by Editorial Manager® and ProduXion Manager® from Aries Systems Corporation

	now updated the docs to explain this better.<br><br>6. The downsampling and NovoSparc modules together might create an opportunity to identify the relative error that is introduced when NovoSparc is used to enhance spatial expression patterns. Although this might be outside the scope of this paper.<br><br>A: We thank the reviewer for this suggestion, although it is indeed outside the scope of this paper. It would be interesting to perform this analysis and potentially identify the applicability area of the novoSpaRc integration.<br><br>7. As mentioned in the Major comments section we were unable to successfully run an example script, but it would be of great interest to the large spatial community if this pipeline can easily be used with other downstream analysis tools, such as Giotto, Seurat, Bioconductor (spatialExperiment class), etc.<br><br>A: Spacemake creates both a text based and a compressed (.h5ad format) Digital Expression Matrix as a result of data-processing. This can be easily imported into Seurat and other downstream tools. Spacemake stores the intermediate and processed data files in the AnnData format which is immediately compatible with the Python based scanpy, or squidpy. For users that use the data structures in R, we recommend the https://github.com/theislab/zellkonverter package from the Theis lab which converts between AnnData and Bioconductor.
**Additional Information:**	
**Question**	**Response**
Are you submitting this manuscript to a special series or article collection?	No
**Experimental design and statistics**<br><br>Full details of the experimental design and statistical methods used should be given in the Methods section, as detailed in our Minimum Standards Reporting Checklist. Information essential to interpreting the data presented should be made available in the figure legends.<br><br>Have you included all the information requested in your manuscript?	Yes
**Resources**<br><br>A description of all resources used, including antibodies, cell lines, animals and software tools, with enough information to allow them to be uniquely identified, should be included in the Methods section. Authors are strongly encouraged to cite Research Resource Identifiers (RRIDs) for antibodies, model organisms and tools, where possible.	Yes

Powered by Editorial Manager® and ProduXion Manager® from Aries Systems Corporation

Have you included the information requested as detailed in our Minimum Standards Reporting Checklist?	
**Availability of data and materials**<br><br>All datasets and code on which the conclusions of the paper rely must be either included in your submission or deposited in publicly available repositories (where available and ethically appropriate), referencing such data using a unique identifier in the references and in the "Availability of Data and Materials" section of your manuscript.<br><br>Have you have met the above requirement as detailed in our Minimum Standards Reporting Checklist?	Yes

Powered by Editorial Manager® and ProduXion Manager® from Aries Systems Corporation

Compiled version of main manuscript & sup material Click here to access/download;Manuscript;spacemake_ms.pdf

Spacemake: processing and analysis of large-scale spatial transcriptomics data

Tamas Ryszard Sztanka-Toth[1,2], Marvin Jens[1], Nikos Karaiskos[1,#] & Nikolaus Rajewsky[1,2,3,4,#]

[1]Systems Biology of Gene Regulatory Elements, Berlin Institute for Medical Systems Biology, Max Delbrück Center for Molecular Medicine in the Helmholtz Association, Berlin, Germany

[2]Humboldt-Universität zu Berlin, Institut für Biologie, 10099 Berlin, Germany

[3]DZHK (German Center for Cardiovascular Disease), Partner Site Berlin, Berlin, Germany

[4]Department of Pediatric Oncology, Universitätsmedizin Charité, Berlin, Germany

[#]Corresponding: nikolaos.karaiskos@mdc-berlin.de, rajewsky@mdc-berlin.de

Keywords: bioinformatics, computational biology, computational pipeline, sequence analysis, spatial transcriptomics, single-cell transcriptomics, reproducibility, modularity, scalability, workflow.

Abstract

Spatial sequencing methods increasingly gain popularity within RNA biology studies. State-of-the-art techniques quantify mRNA expression levels from tissue sections and at the same time register information about the original locations of the molecules in the tissue. The resulting datasets are processed and analyzed by accompanying software which, however, is incompatible across inputs from different technologies. Here, we present spacemake, a modular, robust and scalable spatial transcriptomics pipeline built in Snakemake and Python. Spacemake is designed to handle all major spatial transcriptomics datasets and can be readily configured for other technologies. It can process and analyze several samples in parallel, even if they stem from different experimental methods. Spacemake's unified framework enables reproducible data processing from raw sequencing data to automatically generated downstream analysis reports. Spacemake is built with a modular design and offers additional functionality such as sample merging, saturation analysis and analysis of long-reads as separate modules. Moreover, spacemake employs novoSpaRc to integrate spatial and single-cell transcriptomics data, resulting in increased gene counts for the spatial dataset. Spacemake is open-source, extendable and can be seamlessly integrated with existing computational workflows.

Introduction

Tremendous advances during the last decade led to high-throughput single-cell RNA sequencing technologies (scRNA-seq) that became the state-of-the-art for dissecting cellular

heterogeneity within tissues. Spatial transcriptomics sequencing (STS) technologies present a further vital development that allows the assignment of single molecules to spatial positions, thus obtaining coordinates of gene expression. When spatial resolution is high enough to discern individual cells, this enables the identification of cell types and their interactions in spatial context. Spatial information is crucial in studying cell-cell communication mechanisms within the native tissue context and can yield new insights in disease states [1]. Recently published array-based methods are able to retain spatial information at different resolutions. Slide-seq (and Slide-seqV2) operates with 10μm beads that are evenly and randomly distributed on a 2D surface termed "puck" [2,3]. This size roughly corresponds to single-cell resolution. Other methods, such as spatial transcriptomics or the commercially available 10X Visium, work with a grid of 100μm diameter spots, regularly placed on a square glass (with 200μm distance between the centers), or 55μm diameter spots with 100μm distance between the centers, respectively [4,5]. These methods usually capture between 1-10 cells per spot, depending on the cellular density of the studied tissue. In more recent publications, high-definition spatial transcriptomics recovers gene expression at 2μm spatial resolution [6], while MiSeq Illumina flowcells were used to sequence mouse colon and liver tissues, achieving subcellular spatial resolution [7]. Fluorescent RNA labeling methods also achieve very high, often subcellular resolution, but operate on only a pre-selected panel of genes and are hence restricted to targeted studies of gene expression [1,8,9].

Akin to a technological revolution that took place with the advance of RNA-seq and scRNA-seq, we anticipate STS techniques to become invaluable for better understanding biological processes and mechanisms that lead to diseased states. Dissection of a tumor's transcriptional heterogeneity is a prime example. Tumor progression is an intricate process that involves the coexistence of several cell types within the tumor, such as immune cells, native tissue cell types and abnormally growing tumor cells. While scRNA-seq can accurately identify different cell types and their transcriptional programmes, all spatial information regarding the cellular communication across cell types is lost. This information is critical to characterize spatial interactions within the tumor microenvironment and identify the mechanisms that create suitable conditions for the further progression of the disease, such as angiogenesis and hypoxia.

The various array-based STS methods differ not only in their experimental procedures, but also in the data they output and the associated software provided to process and analyze the raw data. Therefore, researchers who wish to take advantage of multiple methods need to get acquainted with several computational pipelines that operate with different logic and output structures. Such a situation can be time-consuming, perplexing, and can lead to the accumulation of errors when alternating between the different methods. There are a few computational processing tools available to date, namely the spaceranger from 10X [5], the ST pipeline [10] and slideseq-tools [2,3,10]. These tools, however, were developed for one specific STS technology (ST-pipeline and spaceranger for Visium and slideseq-tools for Slide-seq datasets), and are therefore not accommodating

different types of data. Furthermore, they lack a unified framework to enable simultaneous processing of many different samples. Finally, they lack additional functionality, such as sub-sampling or merging of samples, integration of scRNA-seq with spatial datasets or support for troubleshooting of sequencing library construction by using long-read sequencing (Table 1).

		slideseq-tools	spaceranger	ST pipeline	spacemake
**Input data**	Slide-seqV2	✓	✗	✓	✓
	10X Visium	✗	✓	✓	✓
	Seq-Scope	✗	✗	✗	✓
	Other array-based STS	✗	✗	✗	✓
	FISH	✗	✗	✗	✗
**Pipeline**	Customisable processing mode	✗	✗	✗	✓
	H&E integration	✗	✓	✗	✓
	Structured output	✓	✓	✓	✓
	Parallel sample processing	✗	✗	✗	✓
	Graphic QC reports	✓	✓	✗	✓
	Automated downstream analysis	✗	✓	✗	✓
**Additional modules**	Saturation analysis	✓	✓	✓	✓
	Technical replicate merging	✗	✗	✗	✓
	novoSpaRc integration	✗	✗	✗	✓
	Pacbio reads for troubleshooting	✗	✗	✗	✓
**General aspects**	Open-source	✓	✗	✓	✓
	Extendable	✓	✗	✓	✓

Table 1. Comparison of spacemake with other published spatial transcriptomics pipelines.

Here, we present spacemake, a unified computational framework for analyzing spatial transcriptomics datasets produced with Visium, Slide-seq, Seq-scope or any other STS technology. Importantly, spacemake performs data processing and downstream analysis in the same way, resulting in uniform reports and quality metrics that are easier to compare and interpret across different technologies. This renders spacemake an excellent candidate for multi-method projects. Apart from the standardized processing of raw data, spacemake can perform additional analyzes which we organize in different modules: integration of histological staining images, downsampling and saturation analysis, merging of biological replicates, spatial reconstruction of scRNA-seq data or merging of scRNA-seq and STS datasets by using novoSpaRc [11,12] and analysis of long-read sequencing data for troubleshooting. Spacemake is written in snakemake [13] with a back-end logic written in Python. It provides an-easy-to-use command-line interface, through which it can be configured and run using a handful of commands. It readily works with various types of array-based STS methods and allows diverse, user-definable processing modes. Spacemake is versatile and can be used either as a new workflow, or be readily integrated into existing pipelines. Finally, spacemake is open-source and freely distributed through a Github repository.

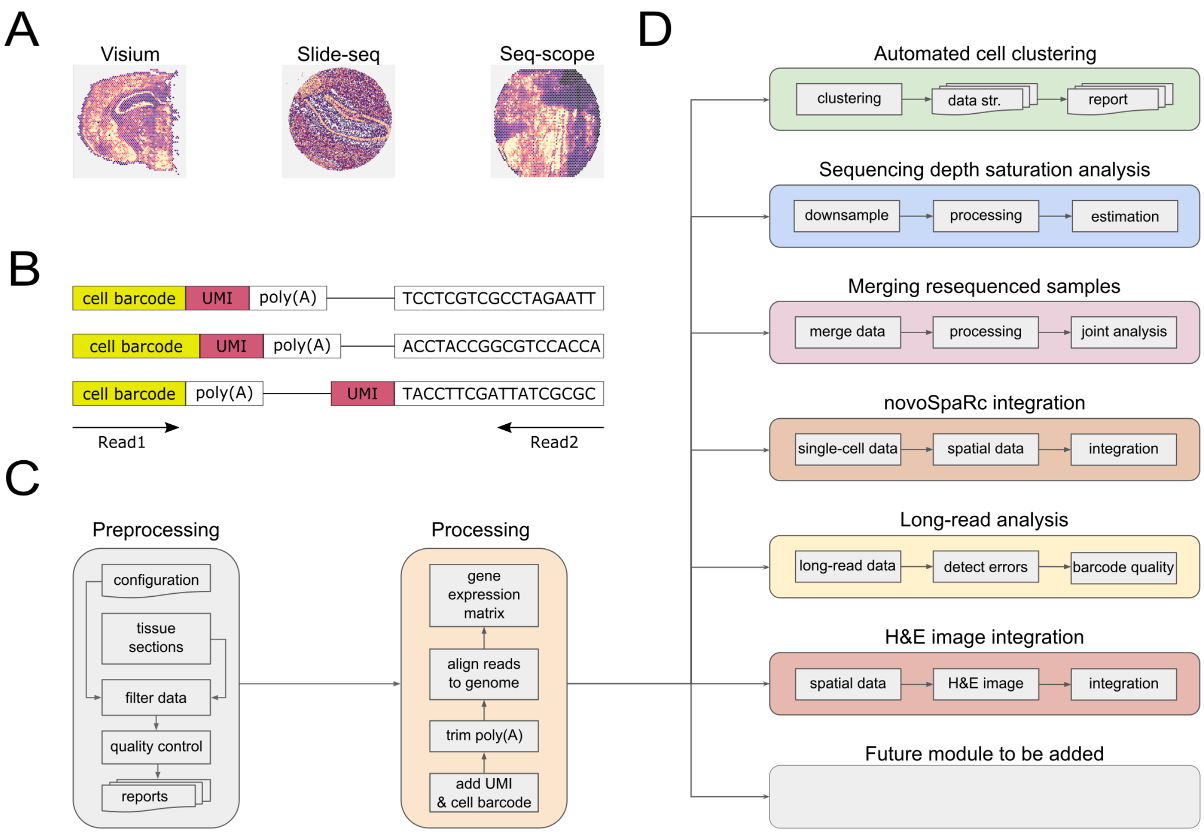

Figure 1. Overview of spacemake. **(A)** Spacemake can handle inputs from different spatial transcriptomics technologies. **(B)** Spacemake is able to handle any barcode strategy. Cell barcode and unique molecular identifier (UMI) lengths are variable, and their position can be on either Read. **(C)** Preprocessing, QC and processing steps. Each sample is processed the same way, regardless of the input type. **(D)** Spacemake is modular and extendable. Each module is implemented with a separate set of rules and commands, and everything is assembled in a top level Snakefile.

Results

Spacemake processes different input data in a single workflow

Spacemake can handle different sequencing-based spatial-transcriptomic datasets, such as those stemming from - but not limited to - Slide-seqV2, 10x Visium or Seq-scope. In particular, it processes raw data (Illumina basecalls or fastq files) in identical fashion, regardless of the sequencing technology or the barcoding strategy of the spatial unit. As STS methods differ experimentally, we employ throughout the text the term spatial unit to describe the fundamental barcoded unit in space, e.g. beads, spots or clusters.

To allow for maximum flexibility, in spacemake each sample is associated with a set of 'sample variables', namely: a 'barcode-flavor', at least one 'run-mode', a 'puck' and a 'species' (Methods). The 'barcode-flavor' describes the barcoding strategy, that is, how the spatial unit barcodes and the unique molecular identifiers (UMIs) should be extracted from Read1 and Read2. The 'run-mode' parameter contains several variables which describe how the sample will be processed downstream and currently include: poly(A) and adapter trimming, tissue detection, multi-mapping read counting, intronic read counting, barcode

cleaning, meshgrid creation and UMI cutoff (Methods). The ‘puck’ parameter allows the user to specify the spatial dimensions and bead diameter size of the underlying STS assay. Lastly, ‘species’ is a pair of a genome fasta file and an annotation file, from which spacemake will generate indices to be used later during mapping. After spacemake is configured and all parameters are set for all samples, it can be run, producing a unified and structured output for each sample (Fig. 1, Methods).

Overview of the spacemake pipeline

Spacemake processes each sample starting from raw reads, which can be either Illumina basecalls, or demultiplexed fastq files. In the first case, spacemake demultiplexes the data using Illumina’s bcl2fastq2 tool [14]. Once raw fastq files have been created, a custom preprocessing script creates an unmapped BAM file: from each Read1, Read2 pair, a spatial unit barcode (or Cell Barcode, CB) and a UMI will be extracted and attached to the unmapped BAM file as CB and MI tags respectively. For each sample, this extraction is based on the previously defined barcode-flavor. Read sequences in this unmapped BAM come from Read2 sequences. Next, using Dropseq-tools [15] adapters and 3’ poly(A) stretches are optionally trimmed from each read. Reads are then mapped with STAR [16] and by using samtools [17] to input the unmapped BAM. After mapping, each read that maps to a gene body will be assigned a gene annotation using the TagReadWithGeneFunction command of Dropseq-tools. If the run-mode has multi-mapper counting turned on, spacemake will process the mapped BAM file line-by-line, and out of all possible alignments keep at most one alignment per read, to be counted later. Specifically, a multi-mapper is kept only if there is exactly one alignment to a genic region and all others to intergenic regions. In this case, the intergenic alignments are discarded. If a read aligns to multiple genes it is discarded. Finally, the digital gene expression (DGE) matrix is created using the DigitalExpression command of Drop-seq tools, with spatial unit barcodes used as a whitelist (Fig. 1). After the DGE matrix is created, each sample is automatically analyzed: data filtering and clustering is done with scanpy [18] and the resulting data is saved as an hdf5 file. At the last step, web-based reports are generated by using Rmarkdown [19] and knitr [20] (Methods).

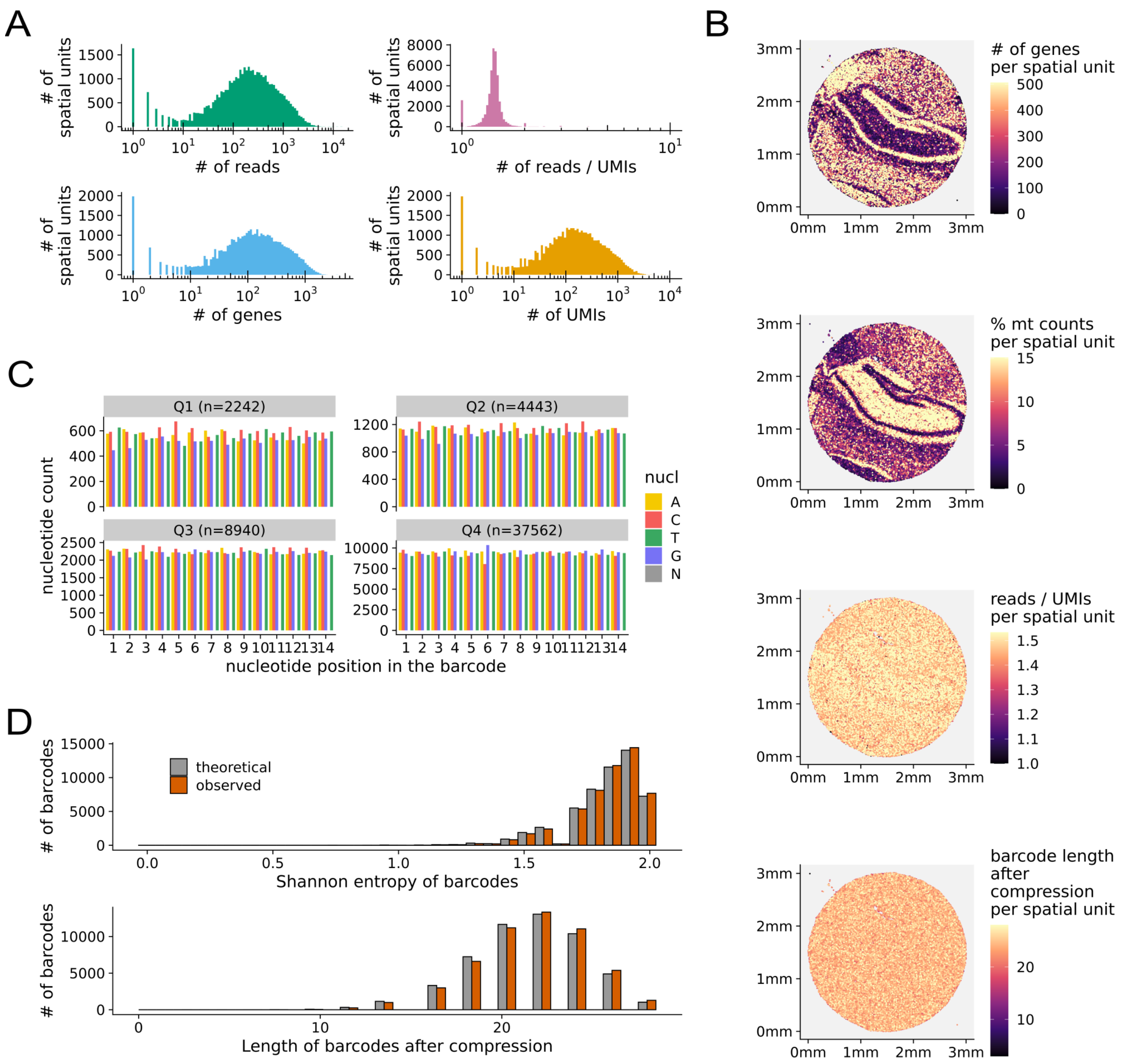

Figure 2. Spacemake produces uniform quality control reports. **(A)** Histograms showing the number of genes, reads, UMIs and reads/UMIs ratio per spatial unit. **(B)** Quality control metrics plotted in tissue space. Top to bottom: number of genes, percentage of mitochondrial counts, reads/UMIs ratios and barcode length after compression, all shown per spatial unit. **(C)** Nucleotide frequencies per barcode position and quantile (segregated by the number of reads). **(D)** Shannon entropy and string compression length of the sequenced barcodes versus the expected theoretical distributions.

Spacemake produces unified quality control (QC) reports

Spacemake assesses the quality of each sample with multiple metrics. The commonly used FastQC [21] tool is first optionally called to assess sequencing library quality by flagging repetitive sequences, adapter content, GC bias, nucleotide composition and basecall qualities among others. Then, each sample is mapped to rRNA with bowtie2 [22] to assess the efficacy of poly(A) mRNA capture relative to abundant, contaminating ribosomal RNAs. After these QC steps are run, a per-sample web-based QC report is generated (Fig. 2). In particular, the number of genes, reads, UMIs and the reads/UMIs ratio are shown both as a histogram over all barcodes (Fig. 2A) and in tissue space (Fig. 2B). Randomness underlies the combinatorial complexity of the barcodes and is required for collision-free encoding spatial

information. To assess the barcode randomness, the spacemake QC contains the following plots: a per-position nucleotide ratio, separated into quartiles by read counts (Fig. 2C, Methods); histograms of the Shannon entropy and the string compression length of the observed barcode sequences against the expected theoretical distributions (Fig. 2C and D, Methods). Barcodes exhibiting unusual distributions in the per-position nucleotide ratio plot would imply artifacts in the sequencing data. Similarly, large deviations from the expected theoretical distributions of the Shannon entropy and the string compression would imply the existence of low complexity barcodes in the data, so that troubleshooting would be required.

Spacemake can readily aggregate spatial units

In some cases, it is useful to join nearby spatial units, effectively trading spatial resolution for statistical power by accumulating read counts (Fig. 3, Methods). This is particularly suitable for irregularly-spaced data points, such as Slide-seq, or when the data stems from an STS assay with subcellular resolution and is hence sparse, such as Seq-scope[7]. In addition, this aggregation also facilitates the comparison of spatial technologies operating at different resolutions, for instance Slide-seq and Visium.

In Seq-scope, for instance, ~800,000 barcodes spread out on a 1x1mm^2 surface, so that the underlying diameter of each spatial unit is smaller than 1µm and contains a very low (not more than a few dozen) number of transcripts. To efficiently analyze such a sparse dataset, it is practical to create a 'meshed' grid (meshgrid) *in-silico*, where the diameter of each newly created spatial unit is 10µm, the approximate size of a eukaryotic cell. Spacemake offers two types of meshgrids out of the box: (1) a Visium-style meshgrid, where circles with a certain diameter are placed at equal distances from each other in a hexagonal grid (Fig. 3A); (2) a hexagonal meshgrid, where equal hexagons are created on top of the whole dataset, without holes in between (Fig. 3B). As the hexagonal meshgrid covers the entire area, no counts are discarded. For both meshgrids, spatial units falling into the same hexagon/circle are joined together and their gene expression counts are summed up (Fig. 2A,B).

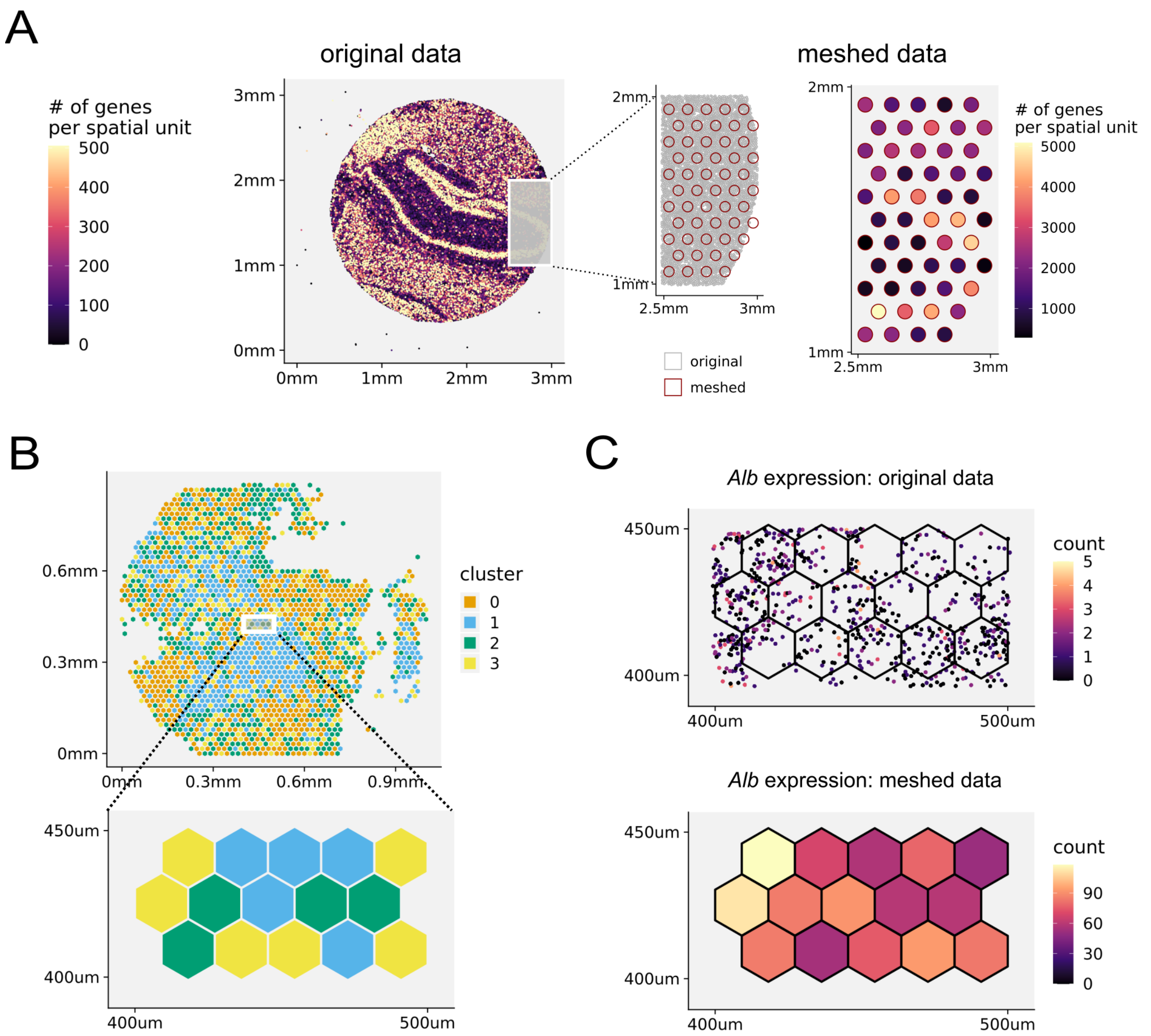

Figure 3. Spacemake seamlessly aggregates spatial units. **(A)** Spacemake can automatically create a Visium-style mesh grid (55µm diameters in a 100µm distance; also user-defined) and further processes the data mapped on this mesh. **(B)** Running on subcellular resolution datasets, such as Seq-scope, spacemake utilizes mesh-creation to join sub-cellular diameter spots into a 10µm-side hexagonal mesh. After the hexagonal mesh is created, downstream analyzes use it as input, *e.g* for cell type identification. **(C)** The highest expressed gene for this adult mouse liver sample is shown. Top right: raw-counts in the subcellular spots; bottom-right: counts assigned to hexagonal mesh cells.

Downsampling analysis reveals library complexity and depth saturation

To assess library complexity and if saturation has been reached in scRNA-seq or STS experiments, a downsampling analysis is employed to estimate whether resequencing would result in a higher number of molecular counts per spatial unit. In spacemake, saturation analysis is implemented as a separate module (Fig. 4, Methods). First, the final BAM file is subsampled to 10%, 20%, …, 90% of the total reads using sambamba [23], and for each ratio a separate DGE matrix is generated. A saturation report is then compiled where median metrics are plotted as a function of the downsampling ratio (Fig. 4A). From the linearity of this curve it can be deduced that saturation has not yet been reached for this Seq-scope sample, even at 10^9 sequenced reads. In addition to plotting the median values, spacemake

also reports histograms for each downsampling ratio per spatial unit, showing the global pattern rather than a single value per ratio (Fig. 4B, Sup. Fig. 3E).

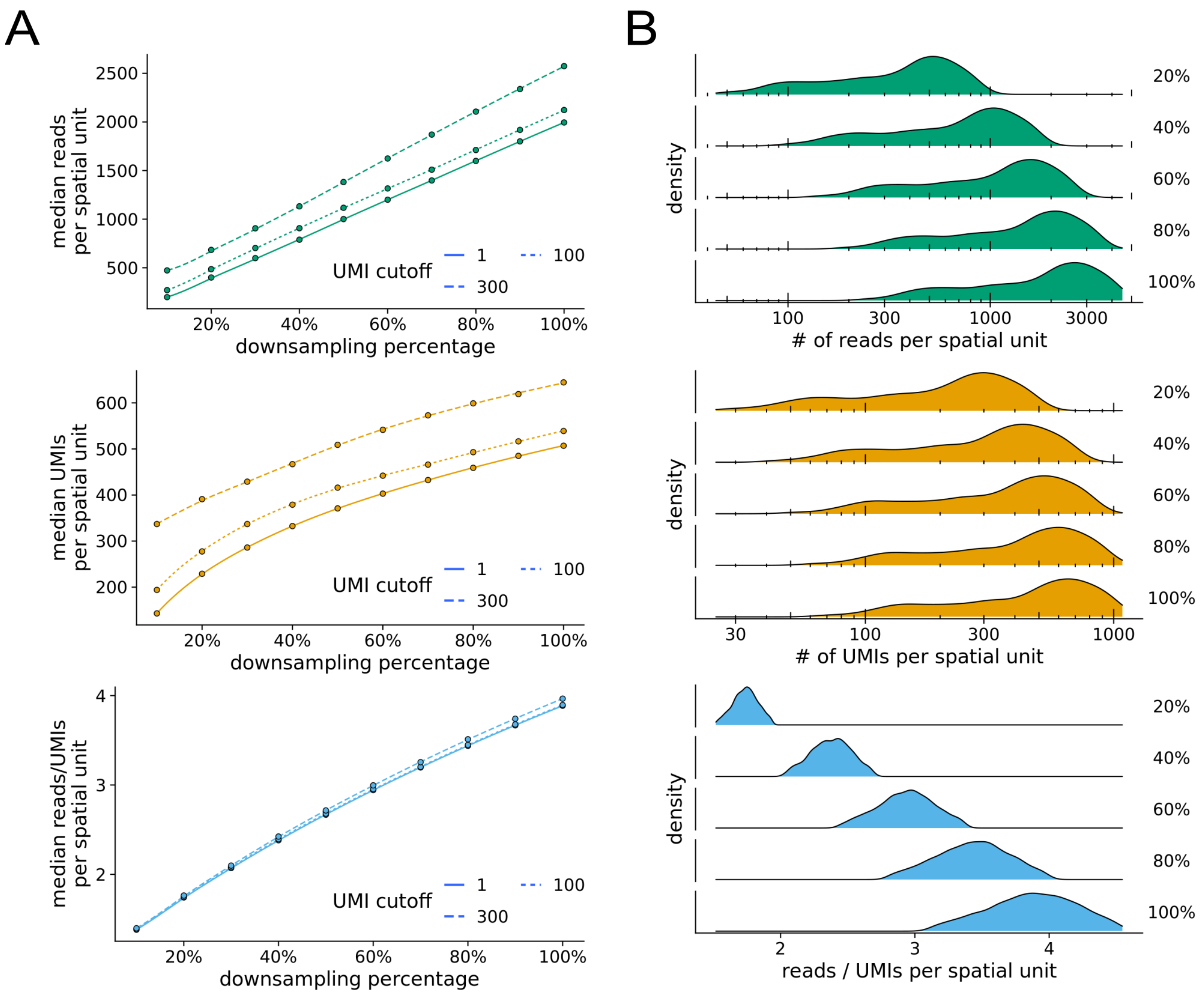

Figure 4. Spacemake can readily downsample the data to perform a saturation analysis. **(A)** Median number of reads, UMIs and reads/UMIs ratios per spatial unit are plotted against the downsampling percentage. Saturation analysis reveals that this Slide-seq sample hasn't reached saturation yet, as the median UMIs curve hasn't reached a plateau. **(B)** Density plots of a Seq-scope downsampled dataset.

Spacemake can readily merge technical replicates

Resequencing a library of sufficient complexity is a common practice to achieve higher molecular counts. A single experiment can result in several sequencing runs, with each of these replicates being technical, as the underlying library is the same. In these cases the original and resequenced dataset have to be joined together, so that counts are quantified in the DGE matrix by properly removing duplicate reads. In spacemake, this process is implemented in the sample merging module which inputs the two separate, already processed datasets and joins them. If a sequencing run was repeated for a sample, the user can add both samples separately to a spacemake project, and later merge them using the spacemake command line (using the `spacemake projects merge_samples` command). After this step a new, merged sample is created and this sample will be processed in an identical

manner downstream as the individual non-merged samples. As a result, this module significantly reduces the hands-on computational analysis time when processing technical replicates.

Spacemake offers a spatial reconstruction baseline of scRNA-seq data

Although spacemake is primarily designed to process STS datasets, it can also efficiently process data produced by the more standardized and popular scRNA-seq technologies. By now several pipelines exist for analyzing scRNA-seq data, for instance [24]. None of these, however, aims at incorporating a spatial reconstruction to the analysis. For this, spacemake utilizes novoSpaRc, a computational framework that reconstructs spatial information solely from scRNA-seq data based on the hypothesis that cells which are spatially neighboring also share similar transcriptional profiles [11,12]. Although novoSpaRc greatly benefits when a reference atlas of gene expression is available, its *de novo* mode is powerful and can yield insights into sub-structures of complex tissues, such as liver lobules, the intestinal epithelium or the kidney [11]. Spacemake employs novoSpaRc to yield a basic spatial reconstruction of scRNA-seq data that can serve as a baseline and can be used to derive further insights (Fig. 5, Table 2, Methods). Applied to a dataset of an adult mouse brain, for instance, spacemake recovers the basic structure representation of the mouse brain cortex when compared to the Allen Reference Atlas [25](Fig. 5A).

**Data produced**	**Publicly available data**	**novoSpaRc mode**	**Outcome**
-	scRNA-seq	*de novo*	basic spatial reconstruction
-	scRNA-seq +spatial	with markers	enhanced gene counts
scRNA-seq	-	*de novo*	basic spatial reconstruction
scRNA-seq	spatial	with markers	enhanced gene counts
spatial	scRNA-seq	with markers	enhanced gene counts
spatial +scRNA-seq	-	with markers	enhanced gene counts

Table 2. NovoSpaRc modes offered by spacemake and their outcome based on data availability.

Spacemake can integrate scRNA-seq data to a spatial transcriptomics dataset

When both spatial and scRNA-seq datasets of the investigated tissue are available, spacemake leverages novoSpaRc to integrate them. For this, the spatial dataset is regarded as a reference atlas and the scRNA-seq transcriptomes are mapped onto the locations of the spatial units. Importantly, spacemake is not restricted to a specific technology but can utilize any spatial dataset as a reference atlas guiding the reconstruction. This becomes especially useful for widely studied or stereotypical tissues for which spatial datasets are already available, such as the adult mouse brain [26]. Mapping a publicly available scRNA-seq dataset [26] onto an existing spatial dataset [27], for instance, results in an enhanced number of genes per spatial unit (Fig. 5). Upon inspection of the spatial patterns of genes

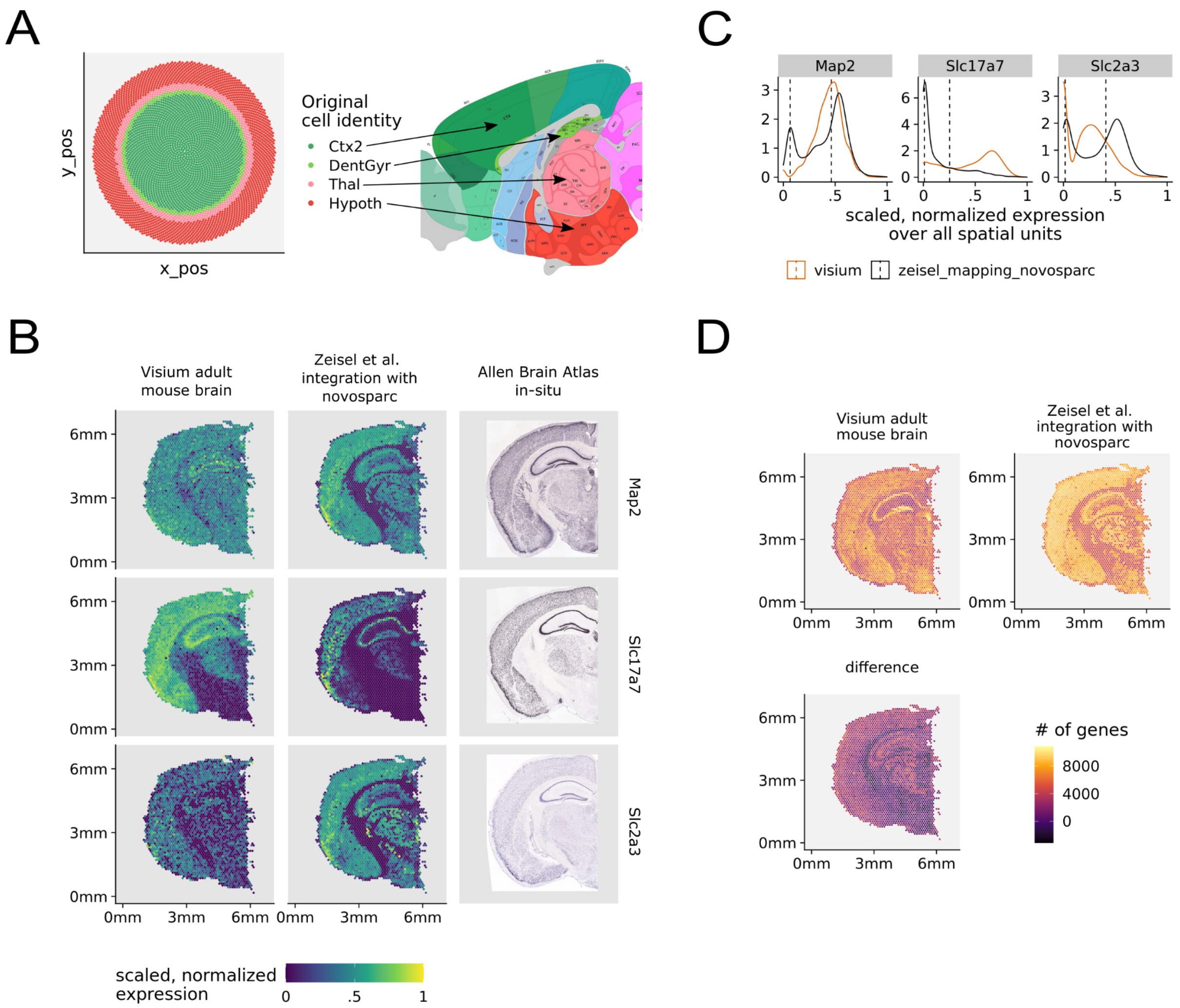

Figure 5. Spacemake can integrate scRNA-seq and spatial transcriptomics datasets. **(A)** Spatially mapping an adult mouse brain scRNA-seq dataset with 30,000 cells onto an *in silico* created circular puck with 5,000 locations reveals cortical layers (left). Tissue labels used: Thal, CA1, Hypoth, Ctx2, DentGyr, SScortex. The identified clusters correspond to spatially distinct anatomical regions (right, adapted from Allen Reference Atlas - http://atlas.brain-map.org) **(B)** Integrating the single-cell and spatial transcriptomics datasets increases the number of genes quantified per spatial unit. **(C)** Expression of spatially informative genes as identified using squidpy. NovoSpaRc integration (right column) results in smoother expression patterns compared to the original ones (left column). **(D)** The bimodal distributions of gene expression are shown together with the corresponding mean values. To arrive at the results of panel (B), the expression of each gene was modeled with a Gaussian Mixture model with 2 components. For each spatial unit, only genes whose expression was in the upper mode were counted.

expressed across all neurons (*Map2, Slc2a3, Slc17a7* taken from http://mousebrain.org/ [26]), the expression profiles become more distinct and defined in space after novoSpaRc integration (Fig. 5B). Moreover, when compared to in-situ images from the Allen Mouse Brain Atlas [28] the expression profiles with scRNA-seq data integration are more similar to the ISH data than those without (Fig. 5B). To quantify the number of genes that are expressed in Visium spots after novoSpaRc integration, we modeled the expression of each gene by using a Gaussian Mixture Model with 2 components (Fig. 5C). Assuming that the lower (upper) mode of the bi-modal distribution describes low-to-no (low-to-high)

expression, we calculated for each spot the number of genes expressed and compared it to the original data (Fig. 5D).

Spacemake can leverage long-reads to troubleshoot library construction

Generation of STS and scRNA-seq libraries can be challenging due to the low amounts of RNA that may be captured from some samples. Especially when protocols are customized to accommodate specific experimental goals and needs, we have found it helpful to investigate our sequencing libraries by long-read sequencing. To this end, spacemake features a module to automatically annotate tens of thousands of long reads against a user-provided reference of expected adapter sequences and other oligo-nucleotides such as primers used during library construction (Sup. Fig. 2, Methods). The module then groups these annotations into recurring patterns of how these building blocks are arranged and provides an overview of the relative contributions of each class of such arrangements to the library. This allows the user to monitor cDNA integrity, for example from 10X Chromium beads (Sup. Fig. 2B,C), and enables to detect and subsequently mitigate potential primer and TSO concatenations as described in [29].

Spacemake offers flexible run-mode settings

A major strength of spacemake are the user-defined run-mode settings. A run-mode is created with the configuration command and provides complete control over how samples using this run-mode should be processed downstream (Methods). Adapter- and poly(A)-trimming can be turned on or off, and multi-mapper and intronic-read counting rules can be set. As each of these settings produces different results (Fig. 6A,B), it is often beneficial to initially run the analysis with several run-modes in parallel and then identify robust and reproducible results.

To demonstrate spacemake's flexibility, we compared it against spaceranger on a publicly available adult mouse brain dataset [27]. First we ran spacemake by using several run modes and then compared the results with that of spaceranger. We focused on two types of correlations between spaceranger and spacemake: (1) gene-gene expression Pearson correlation over all spatial units, treating the data as bulk (Fig. 6B, Sup. Fig. 1C); (2) gene-gene expression Pearson correlation per spatial unit, in space (Sup. Fig 1A,B). We found the (1) correlations to be between 0.48 and 0.99 for all run modes (Fig. 6B, Sup. Fig. 1C) and the (2) correlations to have a median value (per run mode) between 0.5 and 1.0 (Sup. Fig. 1A,B). As we observed that these correlation metrics mostly depend on the highest expressed gene in the dataset (*Bc1*), we further counted the number of genes that are twice as abundant in spaceranger vs spacemake or vice versa (Fig. 6B, Sup. Fig. 1C), and how large the difference of counts between the two processing methods is, per gene (Sup. Fig. 1D). Spacemake produced most similar results to spaceranger when poly(A) trimming is turned

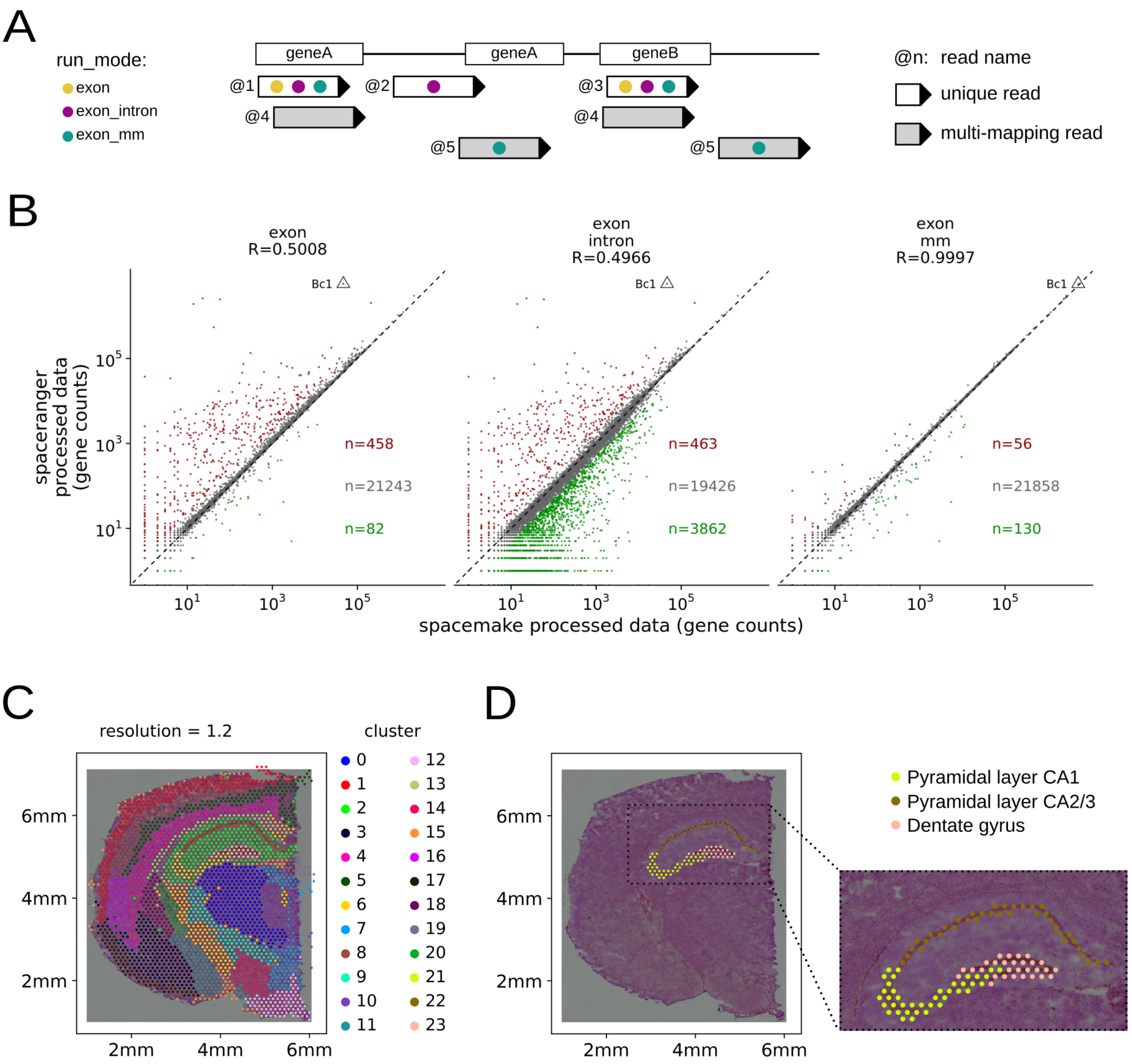

Figure 6. Spacemake offers several processing modes while producing a unified downstream output and can align spatial count data with H&E images. **(A)** Spacemake can be run using several user-defined settings. Gene quantification depends on the run mode set to include reads mapping only on exons; on both exons and introns; on exons and intergenic regions; and whether the reads should be trimmed for poly(A)-tails and adapters. **(B)** Comparison of spacemake run-modes with spaceranger. Genes with twice higher counts are colored red (higher in spaceranger) or green (higher in spacemake); all other genes are colored gray. **(C)** Spacemake automatically performs clustering analysis of the data. At 1.2 resolution clusters become distinct along defined structures in space, such as the cortical layers, CA2/CA3, CA1 and dentate gyrus. **(D)** Spacemake automatically aligns spatial transcriptomics data with H&E images. Here the pyramidal layers and the dentate gyrus as taken from the Allen Brain Atlas, are shown to perfectly overlap with the corresponding clusters.

off, only exonic reads are counted and multi-mapping read counting is turned on (Fig. 6A,B and Sup. Fig. 2).

Building on top of the flexibility offered by run modes, spacemake also allows to cluster the data using different parameters and saves all clustering results in the same automated analysis report (Fig. 6B). For the aforementioned dataset, higher clustering

resolution leads to more biologically meaningful regions identified: at resolution 1.2, for instance, the pyramidal layer of the hippocampus separates into CA1/2, CA3 and the dentate gyrus (Sup Fig. 4A).

Spacemake provides automated downstream analysis

After processing is completed, spacemake performs a basic automated analysis of the data (Methods). For this spacemake employs scanpy [18] and squidpy [18,30]. More specifically, spacemake identifies cell types and their corresponding marker genes and plots them in an automatically generated report. If the user defines multiple UMI cutoffs for performing the downstream analysis, then multiple such reports are generated. For STS datasets in particular, spacemake uses squidpy to generate a cluster-to-cluster neighborhood enrichment heatmap (Sup. Fig. 4B), to calculate co-occurrence of spatial units and predict ligand-receptor interactions between spatial units.

We benchmarked spacemake against the results obtained in a Slide-seqV2 dataset [3]. For this, we first generated a raw fastq file from the slideseq-tools processed BAM file provided by the authors. Then, from the same file we created a DGE matrix using Dropseq-tools [15]. Finally, using the raw fastq files as input we ran spacemake and compared the results with the DGE matrix from the Slide-seqV2 BAM file. Spacemake achieves very high correlation with the Slide-seqV2 data, with most beads having a gene-gene correlation higher than 0.95 and the overall correlation being as high as 0.98 (Sup. Fig. 4B). Spacemake automatic clustering identifies spatially informative clusters, such as the cortical region, mouse hippocampus pyramidal layer, dentate gyrus and thalamic region, and the squidpy neighborhood enrichment analysis reveals spatial closeness of pyramidal-layer and cortical neurons (Sup. Fig. 3C).

Spacemake can automatically align and integrate H&E data

Integrating imaging with spatial transcriptomics data can facilitate the investigation of complex tissues. Spacemake automatically aligns imaging data, such as Hematoxylin and Eosin (H&E) microscopy images, with count-based data (Fig. 6DC, Supp. Fig. 5, Methods). Upon aligning the Visium mouse brain dataset [27] with the corresponding H&E .tiff image, we observed that the pyramidal layer of the hippocampus perfectly aligns with clusters from the automated clustering performed by spacemake (Fig. 6D). To further demonstrate spacemake’s image alignment capability, we downloaded and processed two more public Visium datasets: a sagittal mouse brain section [31] and a coronal mouse kidney section [32] their corresponding images. Spacemake successfully aligned both samples, illustrating that its underlying algorithm works well with Visium data (Sup. Fig. 5C, Methods).

Contrary to Visium images, Seq-scope images do not possess a clear tissue boundary, thus hindering the alignment. Spacemake addresses this by first attempting to deduce the tissue boundaries from the H&E. In case that this fails, the user can manually set the parameters to achieve a better match (Sup. Fig. 5A,B). After identifying the tissue for the

Seq-scope images, spacemake utilizes the same algorithm as for Visium to match the imaging and count datas (Sup. Fig. 5B, Methods).

Spacemake is fast and scales with number of reads

Spacemake is fast, scalable and supports multithreaded processing. To benchmark spacemake, we processed the publicly available adult mouse 10X visium data using both spaceranger and spacemake. We observed that when using 6 cores, spacemake is 1h faster than spaceranger while producing the same results (Sup. Fig. 6A). Spacemake also scales well with the number of reads: for the Slide-seqV2 sample with 70 million reads, total run time was just over 1h, while 1 billion Seq-scope reads took 18 hours to process (Sup. Fig. 6A,B). Moreover, spacemake can run several samples in parallel. For a single sample, spacemake requires 4 cores minimum to run, so that with 8 or 12 cores several samples can be processed together, thus starkly reducing the average running time per sample.

Discussion

As spatial sequencing technologies become increasingly available, the existence of robust, reproducible bioinformatics pipelines is of paramount importance. Here, we present spacemake, a comprehensive computational framework that efficiently analyzes spatial transcriptomics datasets stemming from different technologies. Spacemake is extendable, scalable and provides a complete solution from processing of raw data, over several quality controls and automated reports all the way to advanced downstream analyzes. Spacemake's core strength is the unified processing of different data types, rendering it highly suitable for projects that use multiple methods. Spacemake is open-source, freely available and can be smoothly integrated with other packages that perform downstream analysis [30].

Spacemake is highly modular. It currently contains modules for downsampling and saturation analysis, sample merging, a baseline spatial reconstruction of scRNA-seq datasets and analysis of long-reads, and can be readily extended to add more functionality. Moreover, spacemake is versatile enough and can be used to analyze not only spatial transcriptomics datasets, but also scRNA-seq data. To demonstrate spacemake's capabilities, we have used it to process and analyze Slide-seqV2 and 10X Visium datasets, showing that spacemake accurately reproduces the processed data of the two technologies. We further illustrated how spacemake can integrate scRNA-seq and STS datasets by employing novoSpaRc.

It should be noted that currently, spacemake processes and analyzes sequencing data, but not imaging data. Some spatial transcriptomics techniques, however, require to register the barcodes of the beads or spots in space by imaging. In a companion paper, some of us present a complete computational framework for efficiently handling such datasets, called Optocoder [33]. Spacemake can be readily integrated with Optocoder or similar methods.

Finally, it would be useful to extend spacemake to handle different types of data, e.g. protein expression or chromatin state. As novel techniques that provide diverse molecular

readouts from the same cell are being constantly developed, it will be essential to possess a unified framework that can process the different data modalities. We plan to extend spacemake to accommodate such datasets in the future.

Methods

Run-mode settings

For each sample one or multiple 'run-modes' are defined to describe how spacemake should process it downstream. Each run-mode has a name and several parameters: automatic tissue detection (on/off), poly(A) and adapter trimming (on/off), intronic read counting (on/off), multi-mapping read counting (on/off), data meshing (on/off), number of expected barcodes, UMI-cutoff, DGE matrix cleaning (on/off). Each of these parameters are set through the command line. Currently, spacemake offers the following run-modes out of the box: scRNA_seq, visium, slide_seq and seq_scope, with parameters corresponding to each technology.

Data preprocessing and mapping

The publicly available datasets were obtained as described in the data availability section below. FastQC (v0.11.9) was used to assess sequencing quality and a Python custom script was used to retrieve the cellular barcodes and UMIs for the different read structures (Visium: R1[1-16] for the spot barcode and R1[16-24] for the UMI and cellular barcodes; Seq-scope: R1[1-20] for the bead barcode and R2[1-9] for UMI; Slide-seq: R1[1-14] for bead barcode and R2[15-23] for UMI). During the barcode and UMI retrieval an unmapped BAM was created where each R2 sequence was tagged with the correct cell-barcode and UMI.

Poly(A) and adapter trimming

If poly(A) and adapter trimming is switched on for the current run-mode, the 3' ends of reads are trimmed for poly(A) and overlapping user-defined adapter stretches. This processing is performed with the functions TrimStartingSequence and PolyATrimmer of Drop-seq tools (v2.4.0) for poly(A) and adapter trimming respectively.

Mapping and gene tagging

Alignment to the genome was performed with STAR (v2.7.9a) using the unmapped BAM as input and under the default parameters. The following genomes and annotation files were used: mm10 & M23 and were downloaded from Gencode. Gene tags were added with the function TagReadWithGeneFunction of Drop-seq tools.

Multi-mapping read counting

Multi-mapping reads were counted using a custom python script which parsed the read-name sorted (STAR default output) final BAM line-by-line. For each read name,

maximally one read was kept. If a read mapped to several genomic locations - but only one exonic region - this exonic-mapping read was kept and the rest were discarded. If a read mapped to several exonic locations it was removed altogether. During parsing, each kept read was flagged as primary, and the parsed output (now containing at most 1 read for each multi-mapper) was piped into the DigitalExpression of Dropseq-tools, which was run with a MAPQ=0 filter, to ensure multi-mapper inclusion.

DGE creation

Once the aforementioned steps are run, the DGE matrix is generated. If the provided dataset contains a list of spatial barcodes, it is used as a 'whitelist'. Otherwise, snakemake uses the n_beads parameter of the current run-mode to select the top n_beads number of barcodes with the highest read count using the BamTagHistogram function of Dropseq-tools. Finally, the DGE matrix is generated using either the 'whitelist' of spatial barcodes or the top n_beads barcodes.

DGE barcode cleaning

For a user-defined set of primers, spacemake can optionally discard barcodes that overlap with any of these primers. This is controlled by the clean_dge parameter of a run-mode. When set to true, the following barcodes are removed: (1) barcodes that have at least 4nt overlap with any of the primers in the 3'-end; (2) barcodes that have an at least 7nt overlap with any of the primers, anywhere in the barcode itself. If selected, this step is run before generating the DGE matrix.

Tissue detection

For the samples that tissue detection was turned on, spacemake performed it as follows: first spatial units with UMIs above a certain threshold (provided by the user) were treated as 'under the tissue' spatial units. Then, for each tissue spatial unit its neighboring spatial units were computed. For 10X Visium that is straightforward, as the data points lie within a hexagonal grid. For irregular grids such as Slide-seq datasets, we created a meshgrid and then quantified the spatial unit neighborhoods. This resulted in the generation of contiguous areas. Spatial units lying within the largest contiguous area were then considered to be under the tissue.

Automated downstream analysis

For downstream analysis the text based DGE matrix was first parsed line-by-line using a custom python script to create a sparse matrix (Compressed Sparse Column), and cast as an AnnData object, and finally saved in h5 format to ensure minimal space. Then, the standard scanpy single-cell workflow followed with default parameters. We selected the top 2,000 highly variable genes and 40 principal components to use for clustering using the leiden algorithm [34] and lower-dimensional representation with UMAP [35]. Each sample was

clustered using the scanpy.tl.leiden functions and for several resolution values. Cell type markers were identified with the rank_genes_groups function. For STS datasets squidpy was used by running the built-in squidpy.gr.spatial_neighbors function. Spatial co-occurrence was computed with squidpy.gr.co_occurrence and the ligand-receptor analysis with squidpy.gr.ligrec.

Meshgrid creation

We created the mesh grids *in silico* using the numpy.mesh function. For both grids (Visium-style and hexagonal), a rectangular grid was first created with spot_distance_um (spacemake parameter - user definable) horizontal distances and sqrt(3) * spot_distance_um vertical distances. This mesh was then duplicated and spatially translated, so that the result of the two meshes was a mesh where the distance between any two neighboring points was exactly spot_distance_um. For the Visium-style mesh we joined beads which fall into any circle (with mesh points as circle centers) with a diameter of diameter spot_diameter_um. For the hexagonal mesh we calculated the distance between each spatial unit in the data and the mesh-points, and for each spatial unit we selected exactly one mesh point, the one with the minimum value.

Downsampling analysis

Downsampling analysis was done by first splitting the final BAM file into different percentages with sambamba (v0.6.8). Then the downsampled BAM files were fed into the same processing pipeline described above for further analysis.

Spatial reconstruction with novoSpaRc

The *de novo* spatial reconstruction of the adult mouse brain scRNA-seq data was done with novoSpaRc (v0.4.3) and by using the default parameters and a circular disk as a target space. The top 100 highly variable genes were selected for the reconstruction. For the spatial reconstruction with markers, the corresponding Visium dataset was used to first create a reference atlas. The top 200 highly variable genes were first obtained (both from Visium and single-cell datasets) and 195 of them remained after intersecting them. Reconstruction was done with novoSpaRc and with parameter alpha=0.5. For the *de novo* reconstruction we used single cell data from six areas: Thal, CA1, Hypoth, Ctx2, DentGyr, SScortex. To assign each of the 5,000 positions to one of the annotated areas, for each position the area having the highest median value for that position was picked. In this way, each position was assigned only to one area. Out of the six original areas, four were assigned to at least one position (Ctx2, DentGyr, Thal, Hypoth) and two did not have the highest median probability for any position (SScortex, CA1).

Long-read analysis

The cDNA molecules should contain specific oligo-nucleotide building blocks in the right places, in addition to mRNA sequence and (parts of) the original poly(A) tail. Spacemake first aligns a catalog of such building blocks (SMART primer handles, poly(T), Template Switch Oligo, Illumina sequencing adapters, etc.) via local Smith & Waterman to each read. These alignments are then analyzed jointly for each long read and condensed into "signatures" which identify the presence/absence and relative ordering of each building block. Finally, the observed signatures are counted, compared systematically against the expected signature (for example: P5, bead_start, poly(T), N70X for a DropSeq bead-derived Illumina library) and the following diagnostic plots are generated: graphical breakdown of the library by signatures, zoom-in on bead-related features, mismatch and deletion analysis, as well as histograms of start/end positions for each part of the expected signature. We acquired the publicly available data as described below and every 250th read was selected and analyzed with the spacemake.longread module using the 'chromium' longread-signature.

QC reports

QC plots were created with custom R scripts based on the ggplot2 package (v3.3.5). The automatically generated QC sheets were created with a custom Rmarkdown script which takes the downstream processed and analyzed data files and creates the .html QC report. The Shannon entropy for each spatial unit barcode BC was calculated using the following formula:

$$H_{BC} = -\sum_{n \in BC} f(n,\ BC)\ * log_2(f(n,\ BC)),$$

where $f(n,\ BC)$is the relative frequency of a nucleotide n in barcode BC. The length of string compression for a spatial unit barcode was calculated the following way: first the barcode BC was compressed (such that AAACATTA becomes 3A1C1A2T1A) and then the character-length of this compression representation was returned. The observed values were compared against theoretical values as follows: random barcodes were first generated for each sample and their Shannon entropy and string compression were then computed. The number of random barcodes generated was always the same as the number of real barcodes, for all samples.

External DGE processing

Spacemake offers the possibility to process external count data. In this case, instead of starting from the raw data, the sample is processed downstream from the DGE matrix creation. Spacemake will perform the automated analysis and clustering and generate the corresponding reports.

Alignment of H&E images with count data

Generating binary images from count data

To align H&E images with spatial count data, spacemake first generates gray-scale images from the count data itself. For Visium, a 1000 x 1000 pixel image was generated (corresponding to a 6.5mm x 6.5mm square glass) based on the beads which were previously identified by spacemake as under the tissue (Methods). Each bead was then drawn using the openCV circle() function - according to visium coordinates - with a 100 micron distance from each other, with each bead having 55 micron diameter. Each circle was drawn black and the rest of the image was white.
For the Seq-scope samples, a pixel image was first generated from the actual count data. Raw counts were scaled to have a maximum value of 255, so that the image can be stored as an 8-bit grayscale. Then, pixels were aggregated so that the image has a 1000 x 1000 pixel dimension. Next, a binary filter was run between 190-200 to generate the binary image from grayscale, hence resulting in the final binary count image.

Generating binary images from HE

H&E images were first loaded using the openCV imread() function. Next, grayscale images were generated using the openCV cvtColor() function. Then a binary image was generated using the openCV threshold() function, using automatic thresholding.

Matching binary H&E image and binary count image

To align the H&E image and the count image, we used the openCV matchTemplate() function. This function given a reference image (in our case the binary HE) finds the position at which a template image (in our case the binary count image) has the highest correlation with the reference image. Our algorithm will first scale down the binary count image to be one third of the size of the H&E image, and then gradually the zooming ratio will be increased and the highest correlation will be picked. The zooming itself scales y and x independently, thus ensuring that if the x-y ratios are not matching between the template and the reference, the match would be still found. Finally at the last step, the highest correlation is picked, and the resulting H&E is imaged and saved. Then, this image can be imported using spacemake's attach_he_image function.

Code availability and requirements

Spacemake is freely available and can be found on Github:
https://github.com/rajewsky-lab/spacemake
License: GPLv2
Operating system: Unix
Programming language: Python, R
Requirements: Python 3.6 or higher. R 4.0 or higher.

Data availability

Slide-SeqV2

The Slide-seqV2 adult mouse brain dataset was downloaded from https://singlecell.broadinstitute.org/single_cell/study/SCP815/highly-sensitive-spatial-transcriptomics-at-near-cellular-resolution-with-slide-seqv2 (Puck_200115_08).

Visium

The 10X Visium datasets were downloaded from
https://support.10xgenomics.com/spatial-gene-expression/datasets/1.0.0/V1_Adult_Mouse_Brain (coronal mouse brain);
https://www.10xgenomics.com/resources/datasets/mouse-kidney-section-coronal-1-standard-1-1-0 (coronal mouse kidney);
https://www.10xgenomics.com/resources/datasets/mouse-brain-serial-section-1-sagittal-anterior-1-standard-1-1-0 (sagittal mouse brain)
For each sample we downloaded the original .fastq.gz raw files and processed them with spacemake. For the H&E integration we downloaded the original high resolution .tif images, and resized them to 10% using ImageMagick [36] before integration.

Seq-scope

Seq-scope data was downloaded from https://www.ncbi.nlm.nih.gov/geo/query/acc.cgi?acc=GSE169706. For the analysis shown in this paper the dataset from healthy mouse liver with accession id SRR14082759 was used. We used tile Nr. 2105, 2106, 2107 and extracted the bead barcodes and their positions were from raw fastq files found here https://deepblue.lib.umich.edu/data/concern/data_sets/9c67wn05f?locale=en, with the help of Seq-scope's own script available here:
https://github.com/leeju-umich/Cho_Xi_Seqscope/blob/main/script/extractCoord.sh
For the H&E integration we downloaded the original .jpg files from https://deepblue.lib.umich.edu/data/concern/data_sets/9c67wn05f. We aligned count data from tile 2105 with used wt_4X_2.jpg, for count data from tiles 2106, 2017 with wt_4X_1.jpg.

Single-cell data

For the single-cell and novosparc mapping we used publicly available adult mouse brain data from [26], available here: https://storage.googleapis.com/linnarsson-lab-loom/l5_all.loom. We only used tissue labels comparable with the spatial Visium sample, namely: Thal, CA1, Hypoth, Ctx2, DentGyr, SScortex. We processed the data using spacemake and by treating them as an external DGE matrix.

Long-read data

For long-read sequencing data we used a subset of reads from SRR9008425 and SRR9008429, which were nanopore sequenced cDNA sequences derived from 10X Chromium beads from [37].

Acknowledgements

N.K. was supported by DFG grant RA 838/5-1 and DFG grant KA 5006/1-1. T.R.Sz-T. acknowledges funding from the European Union's Horizon 2020 research and innovation program, under the Marie Sklodowska-Curie Actions (MSCA) grant (721890) and funding from DFG Excellence Cluster 2049/Neurocure. This work has been partially funded by the DZHK.

Competing interest

The authors declare no competing interests.

Author contributions

N.K. conceived, designed and implemented the initial version of the pipeline. T.R.Sz.-T. implemented the pipeline in Snakemake. T.R.Sz.-T. and M.J. developed the pipeline. T.R.Sz.-T designed and implemented all modules, except for the long-read analysis module that was designed and implemented by M.J. T.R.Sz.-T. performed all computational and data analyses except for the long-read analysis which was performed by M.J. N.K. and N.R. supervised the study. All authors wrote the manuscript.

References

1. Rao A, Barkley D, França GS, Yanai I. Exploring tissue architecture using spatial transcriptomics. Nature. 2021;596: 211–220.

2. Rodriques SG, Stickels RR, Goeva A, Martin CA, Murray E, Vanderburg CR, et al. Slide-seq: A scalable technology for measuring genome-wide expression at high spatial resolution. Science. 2019;363: 1463–1467.

3. Stickels RR, Murray E, Kumar P, Li J, Marshall JL, Di Bella DJ, et al. Highly sensitive spatial transcriptomics at near-cellular resolution with Slide-seqV2. Nat Biotechnol. 2021;39: 313–319.

4. Ståhl PL, Salmén F, Vickovic S, Lundmark A, Navarro JF, Magnusson J, et al. Visualization and analysis of gene expression in tissue sections by spatial transcriptomics. Science. 2016;353: 78–82.

5. Spatial Transcriptomics - 10x Genomics. [cited 3 Jun 2021]. Available: https://www.10xgenomics.com/spatial-transcriptomics

6. Vickovic S, Eraslan G, Salmén F, Klughammer J, Stenbeck L, Schapiro D, et al. High-definition

spatial transcriptomics for in situ tissue profiling. Nat Methods. 2019;16: 987–990.

7. Microscopic examination of spatial transcriptome using Seq-Scope. Cell. 2021;184: 3559–3572.e22.

8. Lee JH, Daugharthy ER, Scheiman J, Kalhor R, Ferrante TC, Terry R, et al. Fluorescent in situ sequencing (FISSEQ) of RNA for gene expression profiling in intact cells and tissues. Nat Protoc. 2015;10: 442–458.

9. Xia C, Fan J, Emanuel G, Hao J, Zhuang X. Spatial transcriptome profiling by MERFISH reveals subcellular RNA compartmentalization and cell cycle-dependent gene expression. Proc Natl Acad Sci U S A. 2019;116: 19490–19499.

10. Navarro JF, Sjöstrand J, Salmén F, Lundeberg J, Ståhl PL. ST Pipeline: an automated pipeline for spatial mapping of unique transcripts. Bioinformatics. 2017;33: 2591–2593.

11. Nitzan M, Karaiskos N, Friedman N, Rajewsky N. Gene expression cartography. Nature. 2019;576: 132–137.

12. Moriel N, Senel E, Friedman N, Rajewsky N, Karaiskos N, Nitzan M. NovoSpaRc: flexible spatial reconstruction of single-cell gene expression with optimal transport. Nat Protoc. 2021;16: 4177–4200.

13. Mölder F, Jablonski KP, Letcher B, Hall MB, Tomkins-Tinch CH, Sochat V, et al. Sustainable data analysis with Snakemake. F1000Res. 2021;10: 33.

14. bcl2fastq Conversion Software. [cited 12 Oct 2021]. Available: https://support.illumina.com/sequencing/sequencing_software/bcl2fastq-conversion-software.html

15. Drop-seq-tools. Broad Institute; Available: https://github.com/broadinstitute/Drop-seq

16. Dobin A, Davis CA, Schlesinger F, Drenkow J, Zaleski C, Jha S, et al. STAR: ultrafast universal RNA-seq aligner. Bioinformatics. 2013;29: 15–21.

17. Li H, Handsaker B, Wysoker A, Fennell T, Ruan J, Homer N, et al. The Sequence Alignment/Map format and SAMtools. Bioinformatics. 2009;25. doi:10.1093/bioinformatics/btp352

18. Wolf FA, Angerer P, Theis FJ. SCANPY: large-scale single-cell gene expression data analysis. Genome Biol. 2018;19: 15.

19. Dynamic Documents for R [R package rmarkdown version 2.11]. 2021 [cited 12 Oct 2021]. Available: https://CRAN.R-project.org/package=rmarkdown

20. knitr - Yihui Xie. [cited 12 Oct 2021]. Available: https://yihui.org/knitr/

21. Andrews S. FastQC A quality control tool for high throughput sequence data. 2010. Available: http://www.bioinformatics.babraham.ac.uk/projects/fastqc/

22. Langmead B, Salzberg SL. Fast gapped-read alignment with Bowtie 2. Nat Methods. 2012;9: 357–359.

23. Sambamba: process your BAM data faster! [cited 12 Oct 2021]. Available: https://lomereiter.github.io/sambamba/

24. Wurmus R, Uyar B, Osberg B, Franke V, Gosdschan A, Wreczycka K, et al. PiGx: reproducible genomics analysis pipelines with GNU Guix. Gigascience. 2018;7. doi:10.1093/gigascience/giy123

25. Allen reference atlases :: Atlas viewer. [cited 24 Feb 2022]. Available: http://atlas.brain-map.org

26. Zeisel A, Hochgerner H, Lönnerberg P, Johnsson A, Memic F, van der Zwan J, et al. Molecular Architecture of the Mouse Nervous System. Cell. 2018;174: 999–1014.e22.

27. V1_Adult_Mouse_Brain -Datasets -Spatial Gene Expression -Official 10x Genomics Support. [cited 4 Jun 2021]. Available: https://support.10xgenomics.com/spatial-gene-expression/datasets/1.0.0/V1_Adult_Mouse_Brain

28. ISH data :: Allen brain atlas: Mouse brain. [cited 24 Feb 2022]. Available: http://mouse.brain-map.org

29. Kapteyn J, He R, McDowell ET, Gang DR. Incorporation of non-natural nucleotides into template-switching oligonucleotides reduces background and improves cDNA synthesis from very small RNA samples. BMC Genomics. 2010;11: 413.

30. Palla G, Spitzer H, Klein M, Fischer D, Schaar AC, Kuemmerle LB, et al. Squidpy: a scalable framework for spatial single cell analysis. bioRxiv. 2021. p. 2021.02.19.431994. doi:10.1101/2021.02.19.431994

31. Mouse Brain Serial Section 1 (Sagittal-Anterior). [cited 24 Feb 2022]. Available: https://www.10xgenomics.com/resources/datasets/mouse-brain-serial-section-1-sagittal-anterior-1-standard-1-1-0

32. Mouse Kidney Section (Coronal). [cited 24 Feb 2022]. Available: https://www.10xgenomics.com/resources/datasets/mouse-kidney-section-coronal-1-standard-1-1-0

33. Senel E, Rajewsky N, Karaiskos N. Optocoder: computational decoding of spatially indexed bead arrays. bioRxiv. 2022. p. 2022.02.04.478148. doi:10.1101/2022.02.04.478148

34. Traag VA, Waltman L, van Eck NJ. From Louvain to Leiden: guaranteeing well-connected communities. Sci Rep. 2019;9: 1–12.

35. McInnes L, Healy J, Melville J. UMAP: Uniform Manifold Approximation and Projection for Dimension Reduction. arXiv [stat.ML]. 2018. Available: http://arxiv.org/abs/1802.03426

36. ImageMagick Studio LLC. ImageMagick. In: ImageMagick [Internet]. [cited 9 Mar 2022]. Available: https://imagemagick.org/

37. Lebrigand K, Magnone V, Barbry P, Waldmann R. High throughput error corrected Nanopore single cell transcriptome sequencing. Nat Commun. 2020;11: 4025.

Supplementary Data and Figures

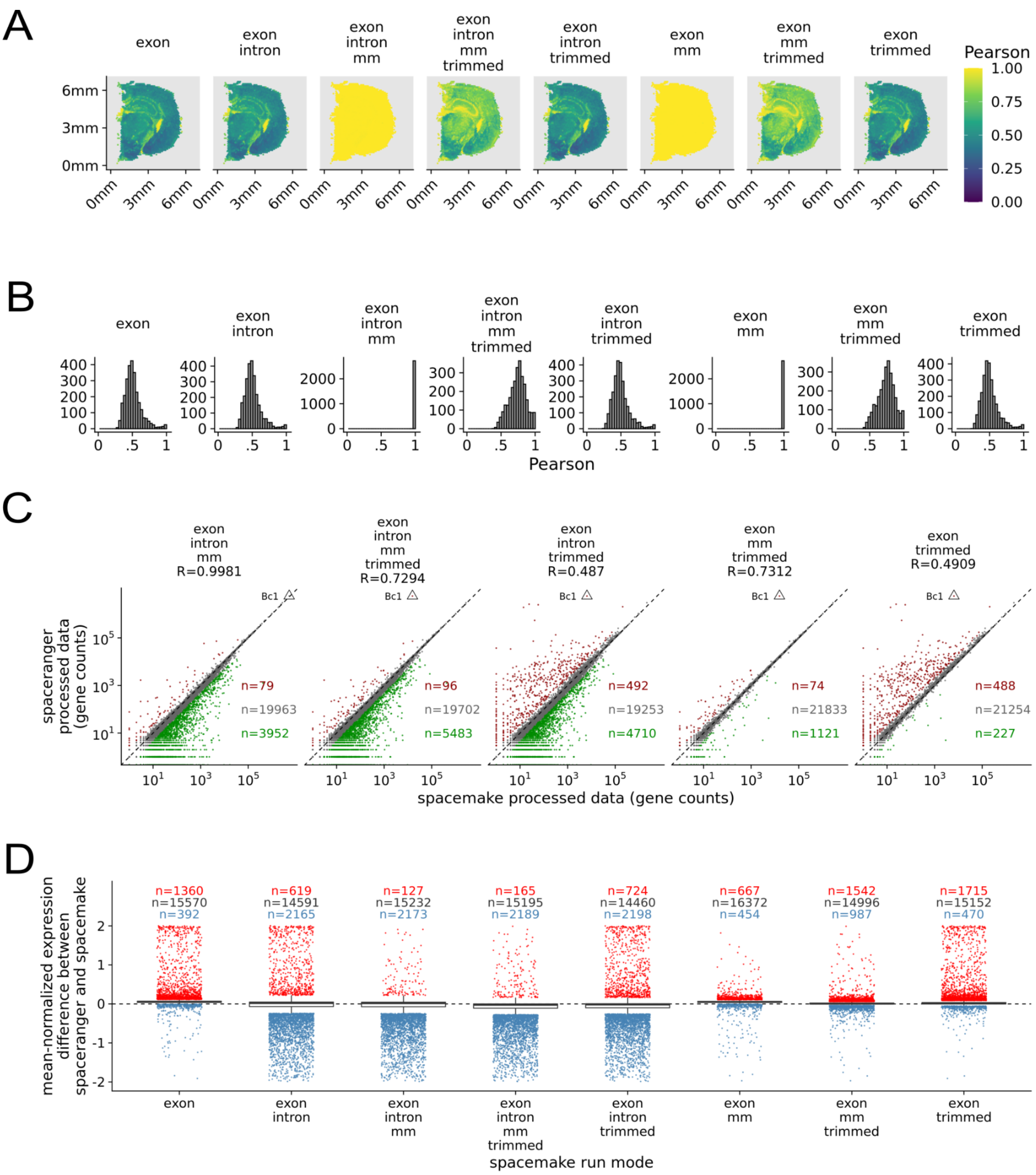

Supplementary Figure 1. Spacemake offers customizable run-mode settings and correlates well with spaceranger. **(A)** Correlations of spacemake run-modes with spaceranger. **(B)** Histograms of Pearson correlations for the different run-modes. **(C)** Correlations of aggregated gene counts. Each dot represents a gene. Red-colored genes exhibit an at least 2-fold increase in spaceranger vs spacemake, while green-colored genes exhibit an at least 2-fold increase in spacemake vs spaceranger. **(D)** Normalized expression differences (per gene) between spaceranger and spacemake run modes. For each gene, spacemake counts were subtracted from spaceranger counts, and the difference was normalized to the mean. Genes expressed higher in spaceranger (spacemake) are colored in red (blue).

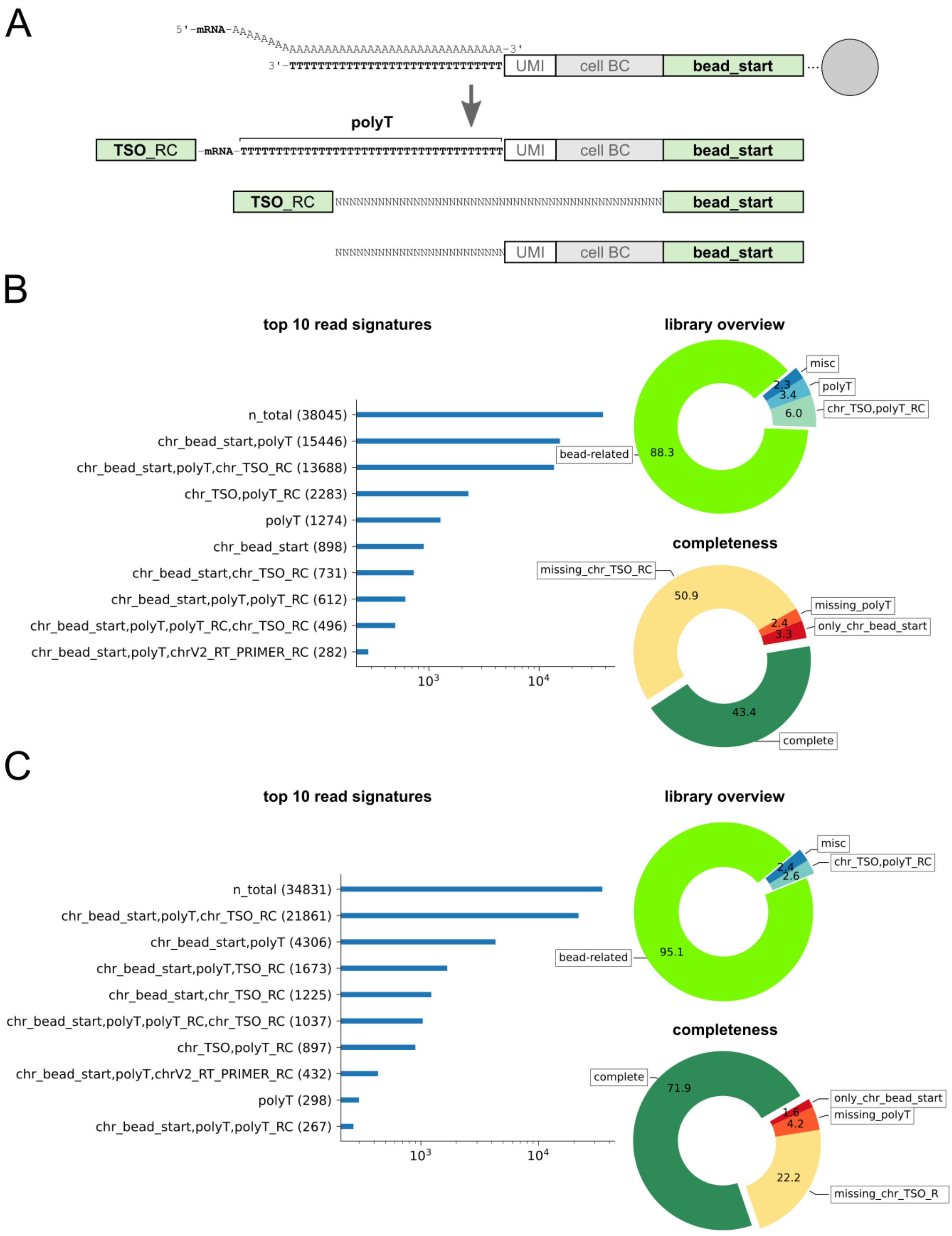

Supplementary Figure 2. Nanopore long-read analysis **(A)** Capture and reverse transcription of mRNA molecules with barcoded beads (top) can lead to the production of different cDNA species (bottom). The expected product contains Template Switch Oligo (TSO), mRNA, poly(T), and the bead-side primer handle labeled 'bead_start'. Identifiable building blocks labeled in bold-face. **(B, C)** Spacemake long-read module annotation of 10X Chromium-derived cDNA from [37]. Left: the most common signatures are plotted on a horizontal bar-chart (log-scaled x-axis). Right, top: overview

donut-plot showing that the majority of cDNAs contain the expected bead primer-handle (bright green). Right, bottom: breakdown of the primer-handle containing cDNAs reveals that < 5% lack a detectable poly(T) tract and that 51% (B, SRR9008425) and 24% (C, SRR9008429) of reads are not terminated by identifiable TSO sequences. Note that oligo block labels for (B), (C) include 'chr_' as a prefix for 10X Chromium specific sequences, whereas (A) applies more broadly, for example also to Drop-seq beads.

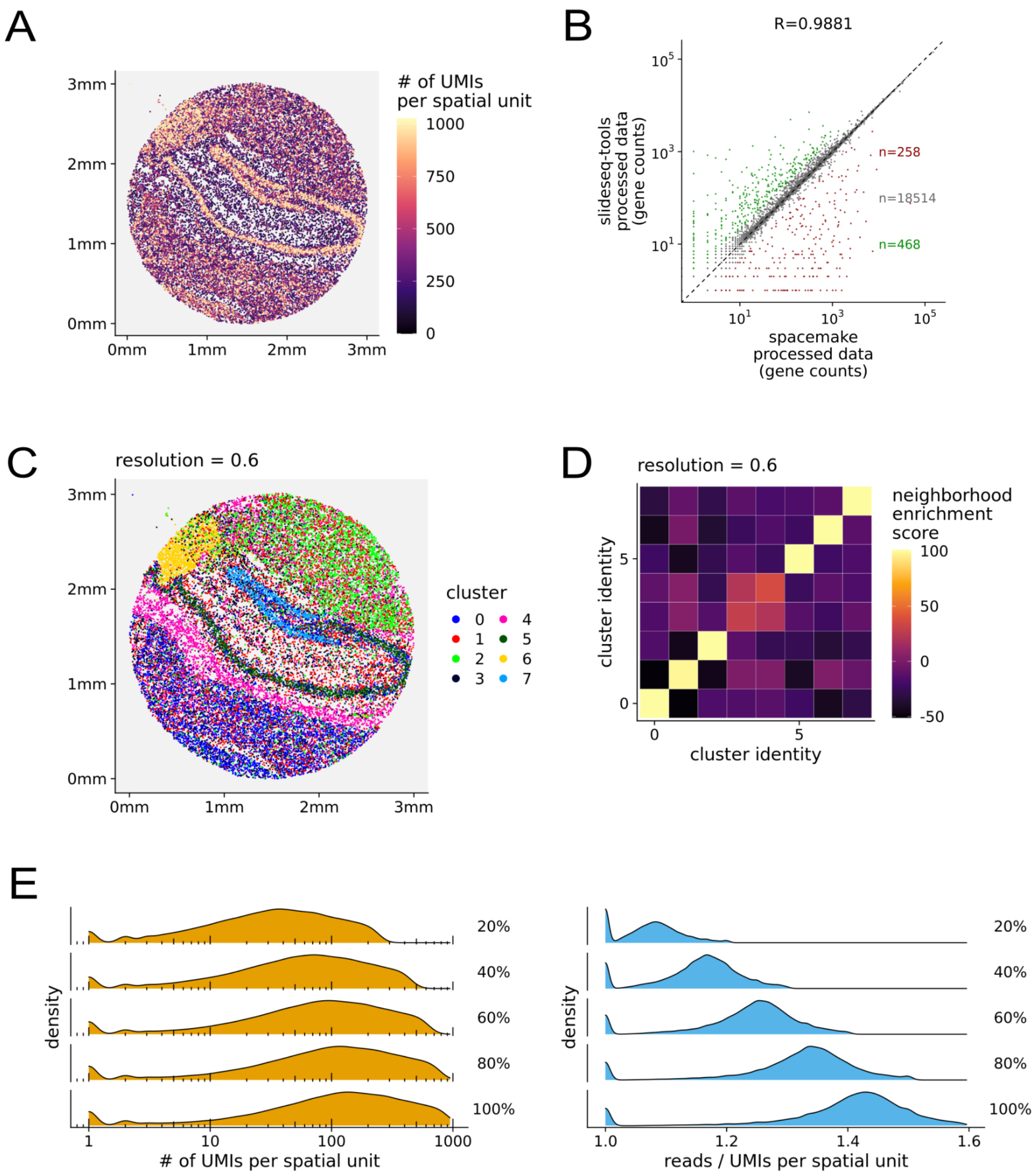

Supplementary Figure 3. Spacemake efficiently processes Slide-seqV2 data **(A)** Distribution of UMIs per bead over the puck. **(B)** Per-gene correlation of slideseq-tools and spacemake. Red genes are 2-fold enriched in slideseq-tools, while green genes are 2-fold enriched in spacemake. **(C)** Automated analysis identifies spatially resolved clusters, such as the cortical layer, dentate gyrus, pyramidal layer and thalamic region. **(D)** Neighborhood-enrichment with squidpy identifies clusters 3 and 4 to be neighboring in space. **(E)** Downsampling analysis reveals that distributions of UMIs per bead increase with sequencing depth (left) while the reads/UMIs ratio remains low (right), indicating that the sample has not reached sequencing saturation.

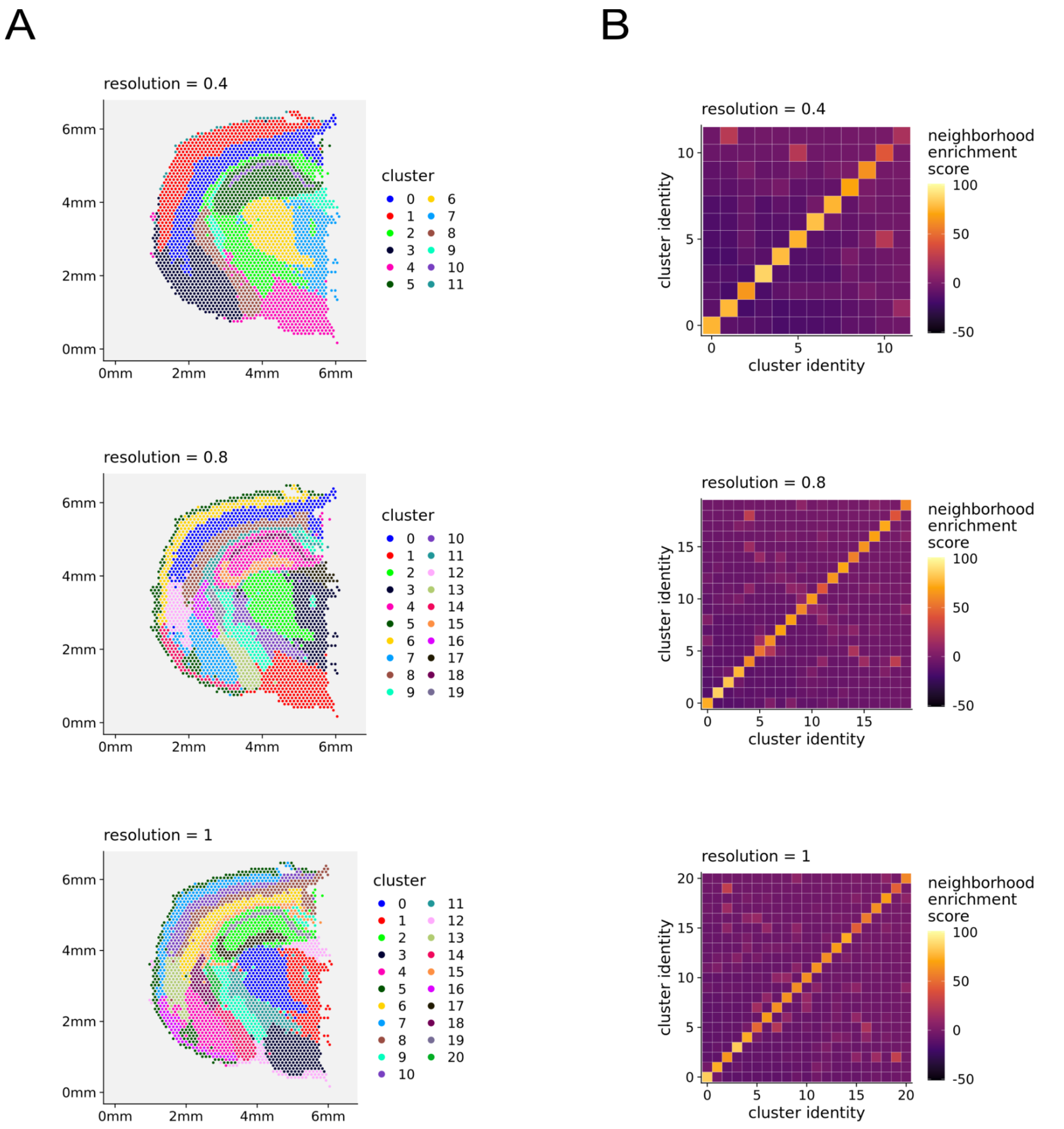

Supplementary Figure 4. Using a higher resolution parameter during clustering leads to more defined clusters in the physical space. **(A)** Higher resolutions result in more clearly defined structures in space. At resolution 0.8 we see a separation in the cortical layers as well as CA1/CA2, CA3 and the dentate gyrus **(B)** The accompanying neighborhood enrichment analysis shows how these clusters interact with each other. Spacemake will run squidpy's neighborhood enrichment analysis for each clustering resolution automatically.

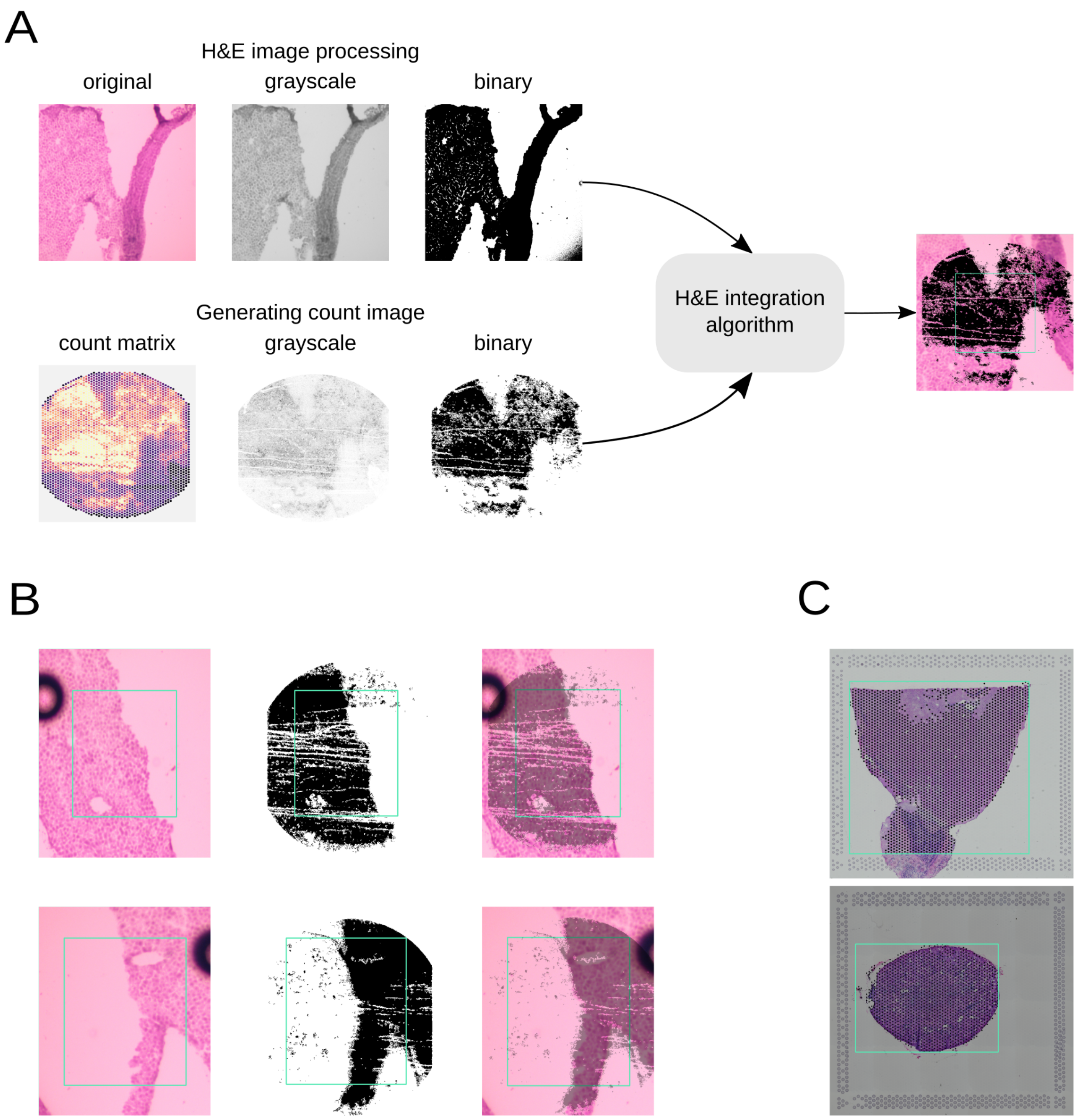

Supplementary Figure 5. Spacemake integrates and aligns spatial count data with H&E images. **(A)** Workflow of the alignment shown for Seq-scope tile 2105. The H&E images are first converted to grayscale; the tissue is then deduced by converting the image to binary (top). From the count data, a grayscale image is first generated with each pixel intensity corresponding to the UMI count in a given square area, after which a binary image is generated to assess the tissue (bottom). The two images are then aligned to find the best match between the H&E and the count data. **(B)** Example alignment for two Seq-scope mouse liver samples: tile 2107 (top) and tile 2106 (bottom). The green box in the middle shows which area of the image was used for alignment. **(C)** Alignment result of a Visium sagittal mouse brain section (top) and a Visium coronal mouse kidney section (bottom). The green boxes here show the identified tissue and the area of the match with Visium spots (shown in black).

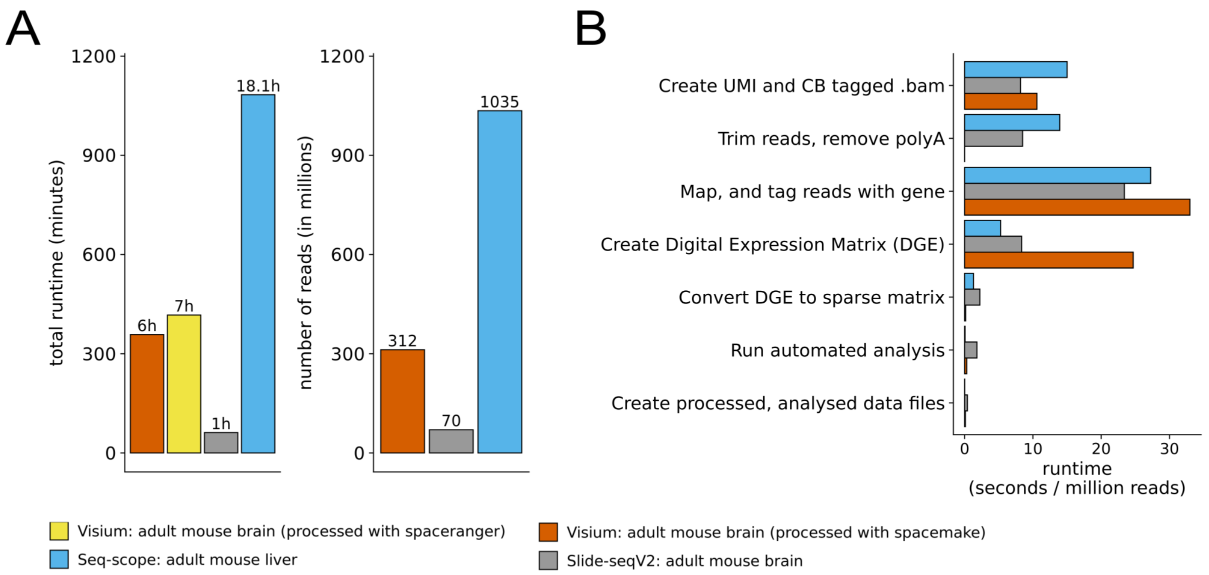

Supplementary Figure 6. Spacemake is fast, scales well and can simultaneously process multiple samples. **(A)** Spacemake is fast and is slightly faster than 10X spaceranger, while offering user-modifiable run-mode settings. Data here shown for a run-mode with spaceranger-like settings (multi-mapping reads counted, no poly(A) trimming, only exonic reads counted). **(B)** Spacemake scales well with increased library size. When normalized to the number of input reads, spacemake running times are similar for every sample, regardless of the underlying sequencing depth. As spaceranger is not modular, running times of the individual processing steps cannot be obtained.
