# Supplementary figures and images for: Spacemake: processing and analysis of large-scale spatial transcriptomics data
Source: Gigascience. 2022 Jul 19;11:giac064. doi: 10.1093/gigascience/giac064 (PMC9295369; doi:10.1093/gigascience/giac064)

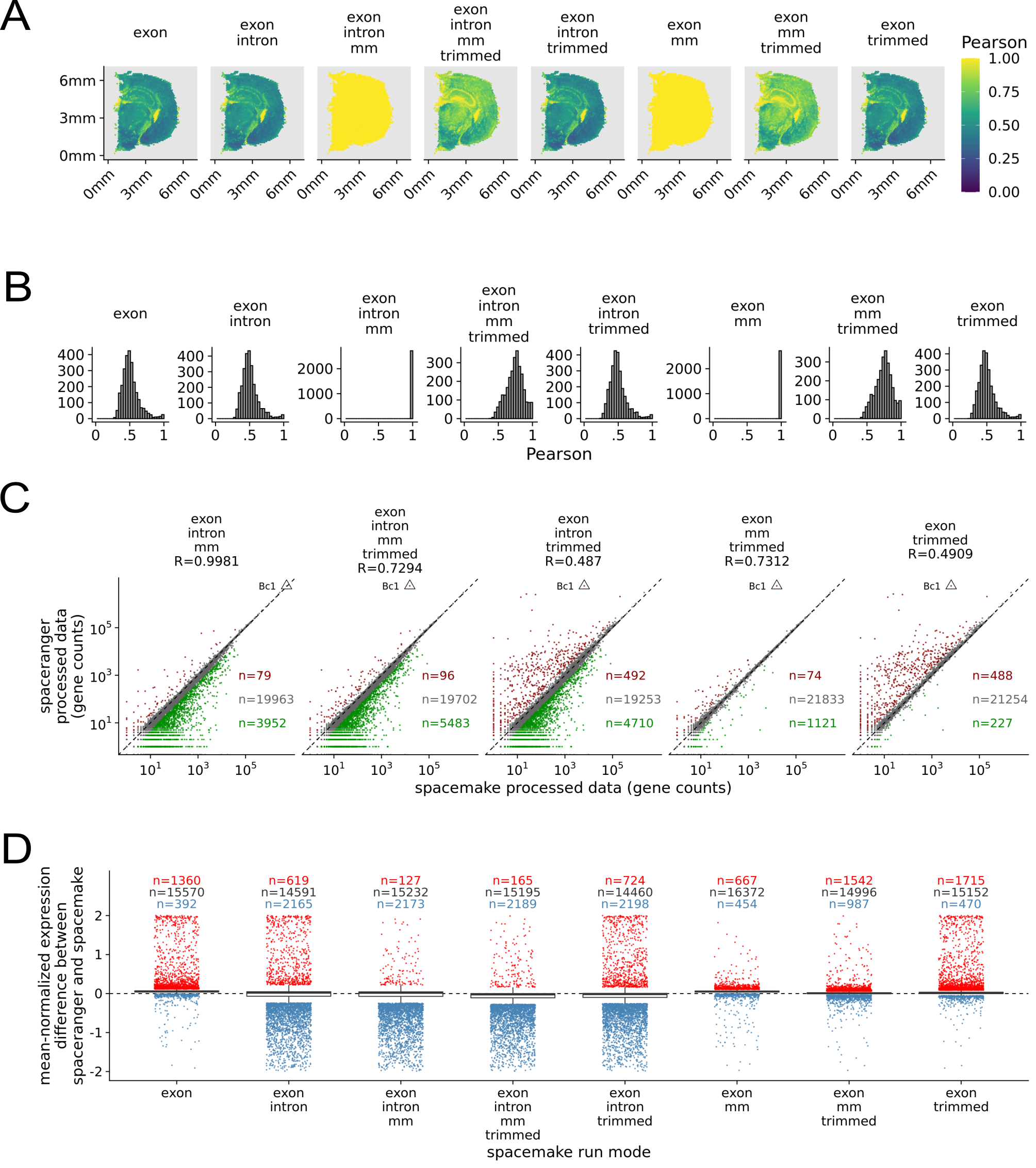

Supplement: giac064_Supplemental_Figures [file giac064_supplemental_figures.zip › supp_figure_1.png]

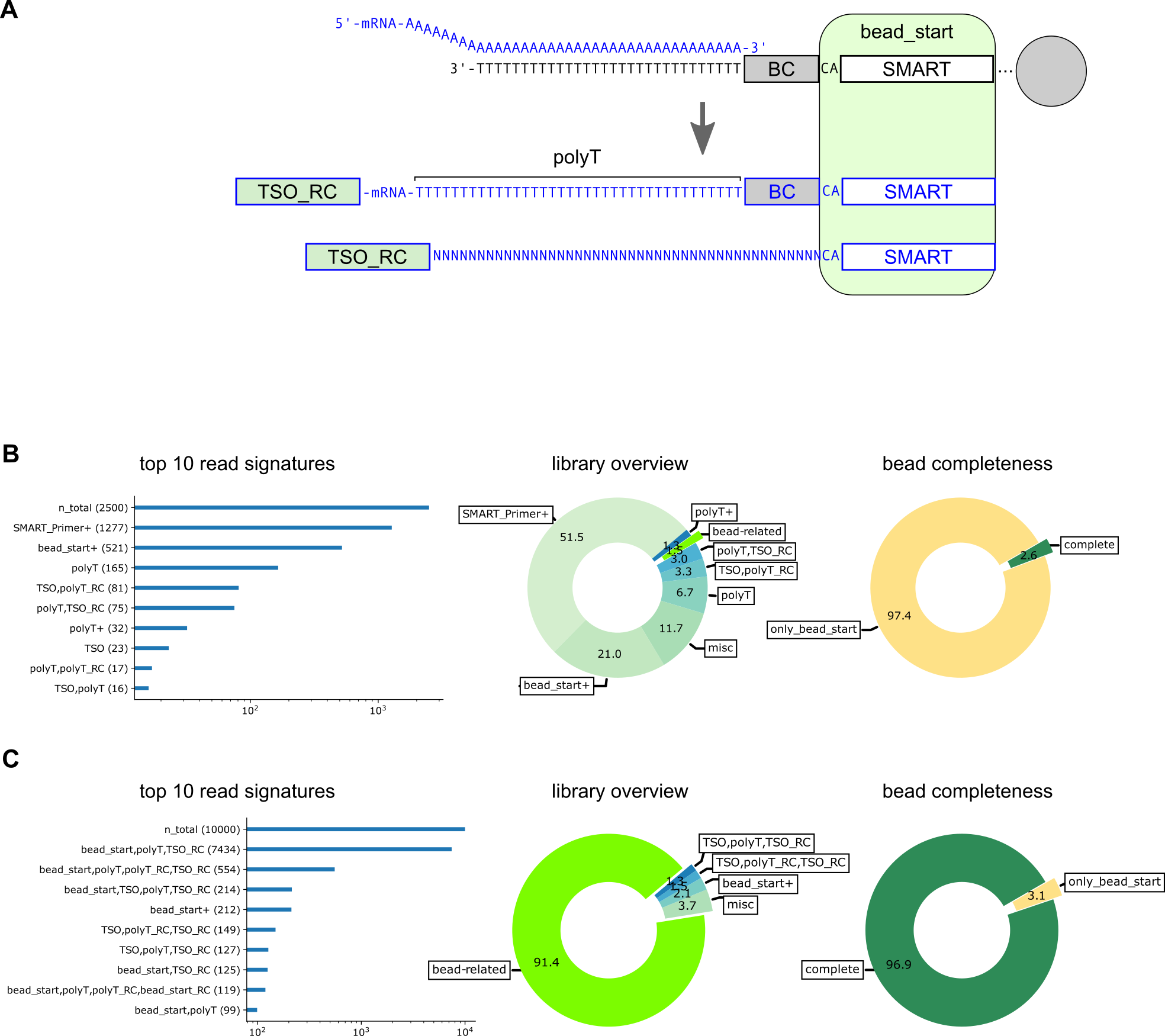

Supplement: giac064_Supplemental_Figures [file giac064_supplemental_figures.zip › supp_figure_2.png]

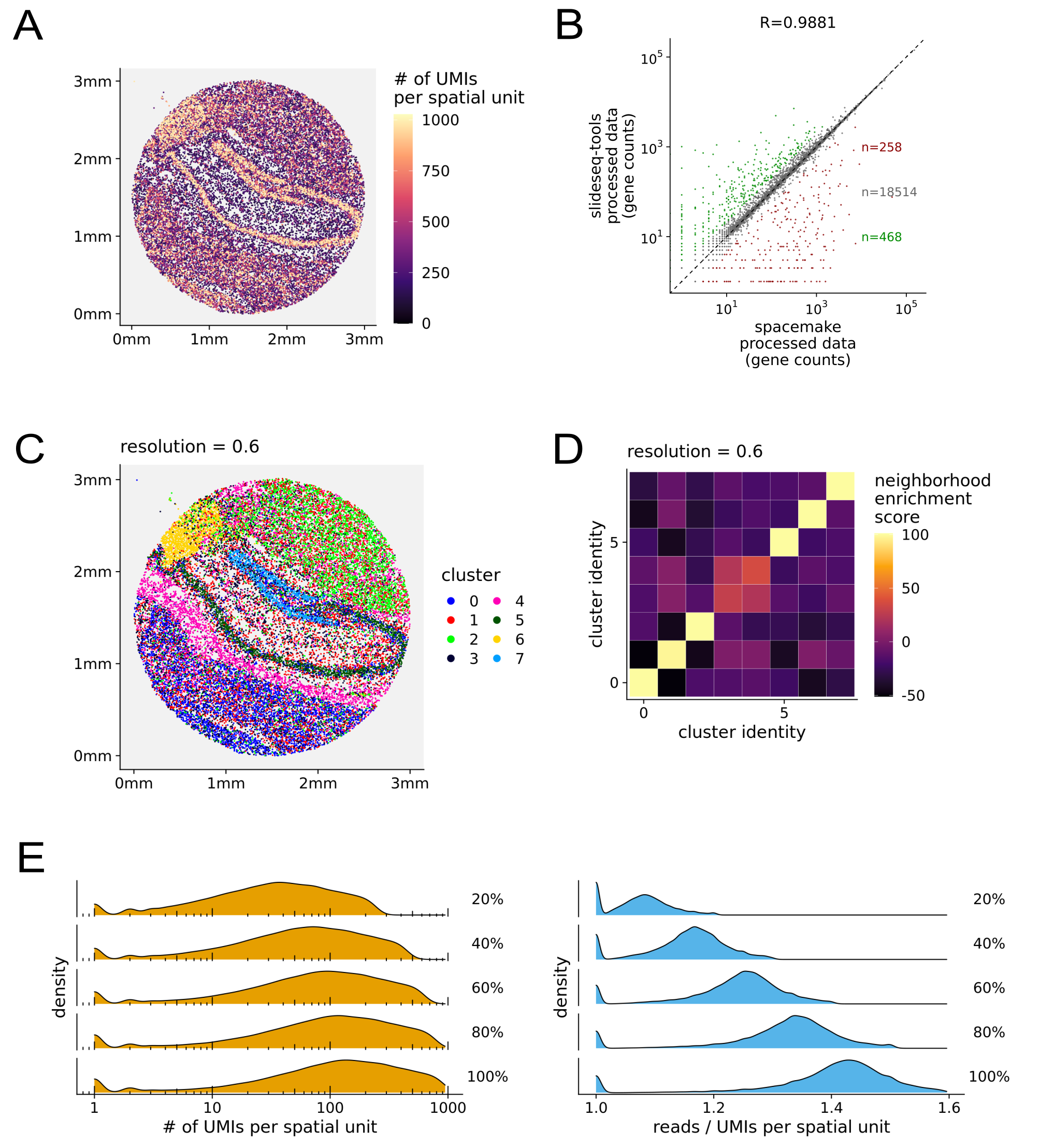

Supplement: giac064_Supplemental_Figures [file giac064_supplemental_figures.zip › supp_figure_3.png]

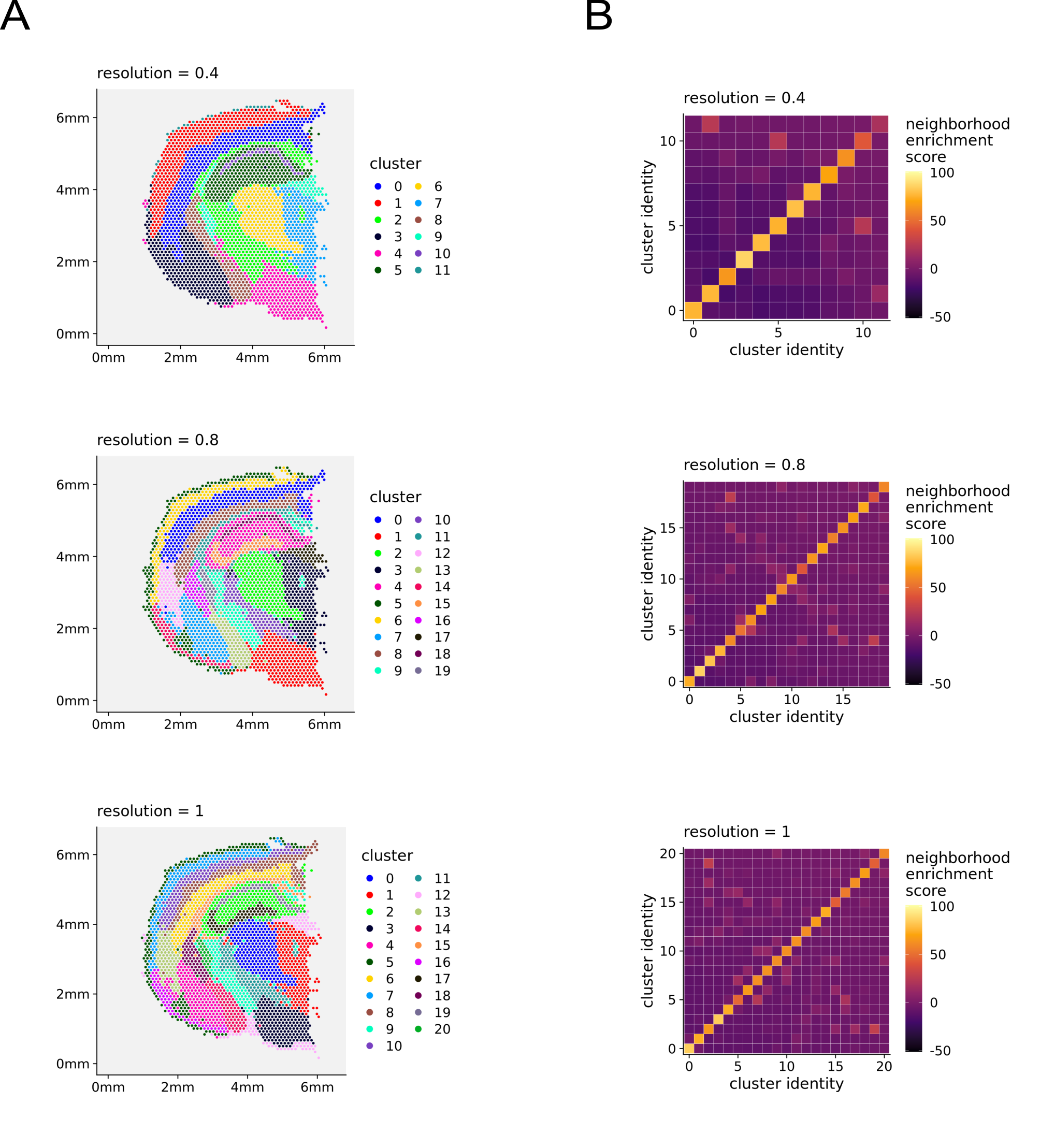

Supplement: giac064_Supplemental_Figures [file giac064_supplemental_figures.zip › supp_figure_4.png]

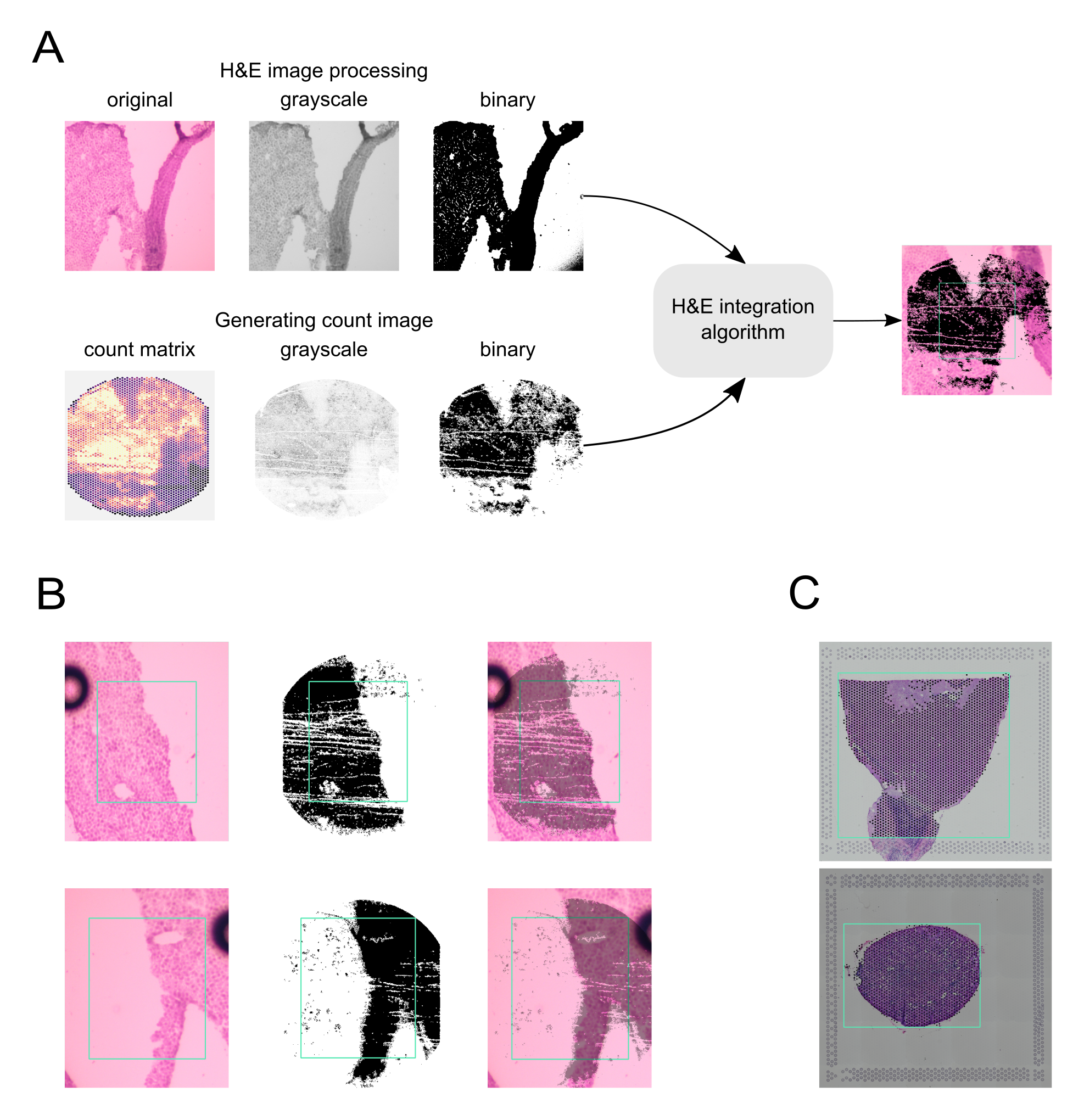

Supplement: giac064_Supplemental_Figures [file giac064_supplemental_figures.zip › supp_figure_5.png]

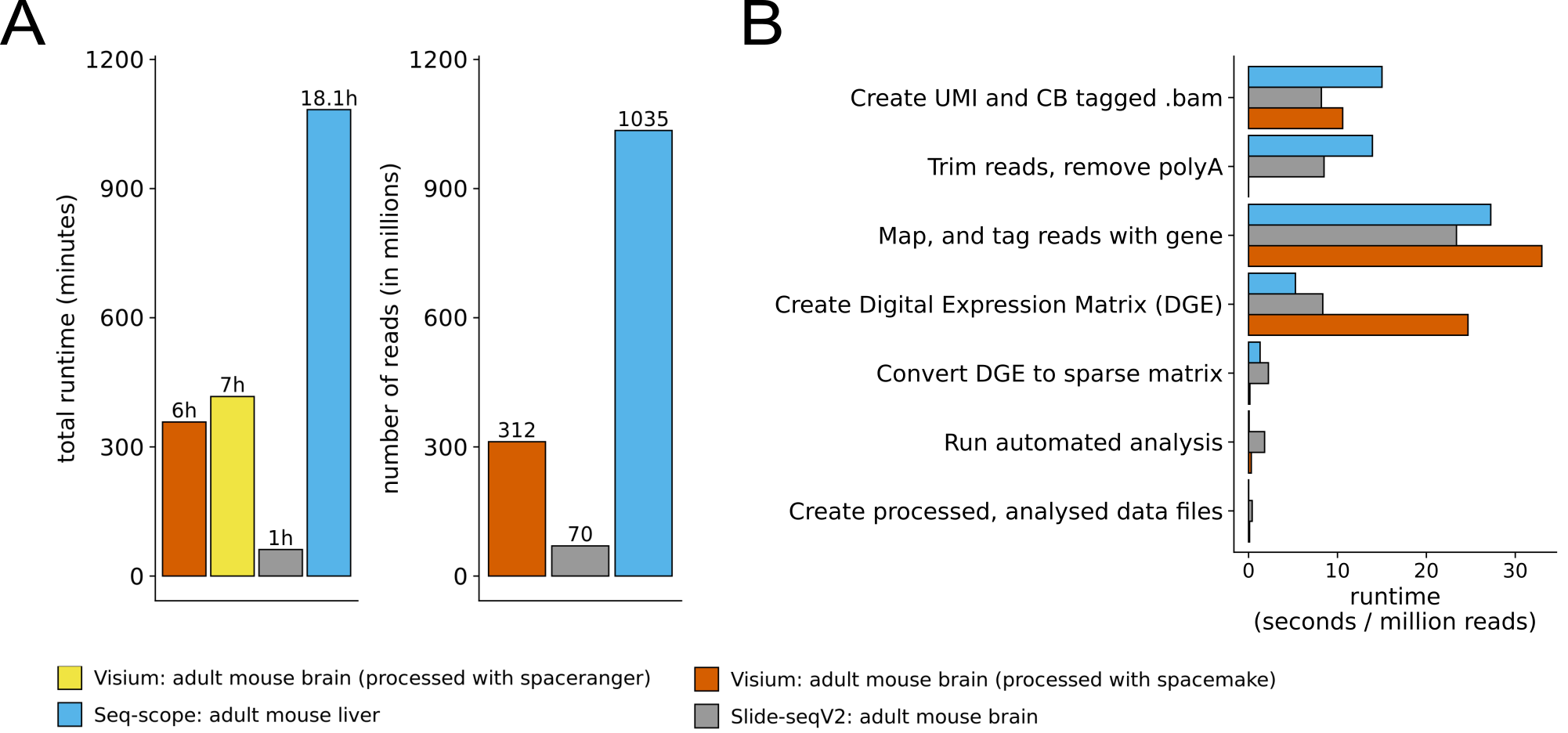

Supplement: giac064_Supplemental_Figures [file giac064_supplemental_figures.zip › supp_figure_6.png]
