# Supplementary material for: Spacemake: processing and analysis of large-scale spatial transcriptomics data
Source: Gigascience. 2022 Jul 19;11:giac064. doi: 10.1093/gigascience/giac064 (PMC9295369; doi:10.1093/gigascience/giac064)
Supplement: giac064_Response_to_Reviewer_Comments_Original_Submission [file giac064_response_to_reviewer_comments_original_submission.pdf]

Author's Response To Reviewer Comments

Close

Dear Editor,

We would like to thank both reviewers for their constructive comments. We have now enhanced spacemake and revised our manuscript accordingly, addressing all raised points.

Importantly, we have introduced a new module in spacemake that integrates imaging and spatial transcriptomics data, for instance images stemming from histological stainings, such as H&E. As H&E stains are common practice in several spatial transcriptomics technologies, we believe that iterating the two data modalities can provide significant insights. We demonstrate spacemake's integration of H&E images in several Visium and Seq-scope samples (Fig 6D and Sup Fig 5, Methods). Further details on the usage of the new module are found in the online documentation of spacemake.

Regarding the installation of spacemake, we have now tested it in several computing environments running UNIX (Ubuntu 18.04, Ubuntu 20.04, CentOS) and could not reproduce the installation problems that Reviewer 2 mentioned. We note that, a critical step in the installation of spacemake, is the existence of a new Conda environment. We have now clarified this further in the online documentation of our tool. The message mentioned by the reviewer is a warning message shown by the pysam library, which arises from backward compatibility issues with htslib (more info below). In fact, this warning message is shown during every spacemake run, even when the pipeline finishes without any errors. As it is difficult to deduce the actual cause of the installation failing from this one warning message alone, we invite the reviewer to send us their snakemake log files, so that we can further investigate the issue.

Our point-by-point responses to the reviewers' comments follows below.

We believe that we have now addressed all comments and have substantially improved both spacemake and our manuscript.

On behalf of the authors,

Dr. Nikos Karaiskos

Reviewer #1:
This manuscript proposed a python-based framework named spacemake, to process and analyze spatial transcriptomics datasets. It offers functionalities including sample merging, saturation analysis and analysis of long-reads as separate modules, etc. Overall, this tool holds promises for spatial analysis, though this manuscript lacks details and explanations of methods and results. Specifically, I have some concerns regarding this manuscript.

1) As shown in table 1, it is noticeable that spacemake doesn't include H&E integration, which is kind of necessary in spatial data. I would recommend the authors at least discuss the potential functionality in including H&E images.

A: We have now included H&E integration in spacemake. An additional module offers automatic integration of imaging and spatial data. For the cases where the H&E image is of low quality, the user can perform a manual integration based on ImageJ. To integrate with ImageJ, the user has first to generate a grayscale image from count data using spacemake, and then align this image with H&E using ImageJ. This is done by first selecting a few (at least 4) corresponding points between the two images, and then transforming the landmark correspondences. Finally, the resulting aligned H&E can be readily attached to spacemake processed datasets.

2) From the legend of Fig 2B, I didn't find the plot with Shannon entropy, please double check.

A: We have corrected the legend of Fig 2B (previously Fig 1B) so that it corresponds to the figure panel shown.

3) I don't understand the meaning of fig 2D. The authors should explain how they calculate the Shannon entropy and string compression length of the sequenced barcodes, as well as how they define the expected theoretical distributions. More details are needed here. Though the authors mentioned related information/details would be in methods (last line in QC section), I didn't find any in methods.

A: The methods for calculating the Shannon entropy and the string compression length were described in the Methods, under the QC reports section. We have now added a sentence in the corresponding passage of the main text describing the interpretation of the plots.

4) In Fig 4 A, the authors show the mapped scRNA-seq of mouse cortical layers. I think a complement spatial plot with annotations is necessary, as there is a gap between Fig 4A and Fig 4B.

A: We have now complemented Fig 5A (previously Fig 4A) with the corresponding sagittal section from a mouse brain to demonstrate the annotations, and recolored the figure so that the identified clusters and anatomy matches.

5) Fig 5C lack the annotations of different colors.

A: We now show the figure legend for Fig 6C (previously Fig 5C), as well as for the newly introduced Sup Fig 4

6) In page 16, the authors cited a manuscript in preparation, which is not good. I suggest remove the citation.

A: We have now adapted the reference and properly cite the manuscript that is publicly available as a bioRxiv pre-print.

7) Supplementary Fig 1 would be better if put as fig 1, thus it would show the overall flow & functionality of spacemake.

A: We agree with the reviewer’s point and have now adapted Sup Fig 1 and have put it as Fig 1.

8) Based on Supplementary Fig 1, the authors should add a section illustrating how they annotate the spatial data and the involved gene markers.

A: We have now modified the header of the respective module in Fig 1 (previously Sup Fig 1) to illustrate that spacemake performs automatic cell clustering analysis and not cell type identification with associated gene markers. The latter would require the existence and the leveraging of a comprehensive cell type dataset, which falls beyond the scope of the current manuscript.

9) The paragraph "Spacemake can readily merge resequenced samples" lacks detailed explanation and results.

A: One of spacemake’s functionalities is the handling of technical replicates, that is, the cases where a library is re-sequenced. In this case, raw data is spread across several files and the user would have to merge them themselves. To facilitate data analysis, we have included a module into spacemake that merges replicates which were already added to the spacemake projects. As this module is purely technical we omit showing figures in the manuscript. Motivated by the reviewer’s comment, we have now updated the corresponding section in the manuscript and further included an explanation in the docs.

10) Though spackemake claims it is fast in processing data, well, Supplementary Fig 5 doesn't fully support that. Meanwhile, the authors should explain what the different colors represent.

A: In Sup Fig 6 (previously Sup Fig 5) it is shown that spacemake processes data at least as fast as spaceranger, if not faster (panel A, red and brown bars). Panel B demonstrates that spacemake scales well with a higher number of reads in the data, as the times in the panel are normalized by the reads number. The different colors correspond to the colors shown in the legend. As spaceranger is not modular, it is not possible to extract from its pipeline the running times for the individual processing

steps. We have now adapted the figure to clarify the different colors, as well as expanded the figure legend with these details.

11) In Supplementary Fig 2, the authors show very high correlation between spacemake and spaceranger, especially the exon intron and exon sub-figures. It looks like the correlations is close to 1. I suggest the authors double check the results and give explanations on their correlation analysis.

A: We thank the reviewer for insisting on this point. We have now double checked and verified the results of our correlation analysis. There is indeed a high concordance between spacemake and spaceranger when multi-mapper reads are included in the quantification, as the vast majority of genes (>21,800) fall along the diagonal. When multi-mapper reads are excluded, the correlations drop to R=0.4966 and R=0.7312, due to a large number of genes (~4,200 and ~4,000) that fall out of the diagonal. Overall, however, the correlations are largely driven by the most highly abundant gene (Bc1). Therefore, we have complemented our analysis by showing boxplots of the difference in normalized gene expression between spacemake and spaceranger (Sup. Fig. 1D).

Reviewer #2
In this article, the authors created a modular and scalable pipeline to process raw sequencing data from spatially resolved transcriptomic technologies. In contrast to other popular genomics technologies, such as (single-cell) RNA sequencing, there are virtually no existing public tools that allow users to quickly and efficiently process the raw spatial transcriptomic sequencing data that are generated through Illumina sequencing. This is largely due to the fact that each spatial transcriptomic workflow creates its own unique spatially barcoded reads and thus typically requires technology-specific tools or scripts to extract both the barcode and gene expression information. Here the authors created Spacemake which consists of multiple modules that are tied together using the popular workflow management system Snakemake. The innovative part of Spacemake comes from the creation of specific 'sample variables', such as the barcode-flavor, run-mode and puck, which
allows them to create a flexible pipeline that in theory can be adapted to any type of spatial array-based sequencing technology. The authors use well-established tools for downstream quality control and data processing and provide useful additional modules to assess or improve spatial data quality. Finally, Spacemake is also directly linked to Squidpy for downstream analysis and creates a web-based report, which could certainly help to lower initial spatial data analysis barriers.

Overall, the presentation of the tool and the methods used in the pipeline as described in their contents are comprehensive and the user manual is easy to understand. We appreciate the efforts to provide this tool to the spatial transcriptomics community and to make it open-source and flexible. However, we do have some suggestions and concerns regarding the manuscript and/or use of this tool.

Major comments:
1. We managed to install the spacemake software on the linux based server but failed to install it on a MacOS machine due to the compatibility issue with bcl2fastq2. Unfortunately, we also ran into an issue on our linux server, which happened during one of the reading steps from "/dev/stdin" in the middle of the spacemake workflow. More specifically we encountered the following error:

Job error: Job 7, TagReadWithGeneFunction
Error message: [E::idx_find_and_load] Could not retrieve index file for '/dev/stdin'

Even with the help of our IT team we were unable to resolve this issue. To help troubleshoot it might be helpful if the authors can provide exact commands for the examples provided in the manuscript and show what should be expected output of each job in the snakemake pipeline. As a result we were unable to re-run any of the provided examples, which severely limited our reviewing options.

A: The error mentioned by the reviewer should not pose an issue, and is expected. It is produced by pysam as a warning when there is no index file available for a BAM file, even though it is not required (spacemake requires a pysam version of at least 0.16.0.1). A thread on github explains this: https://github.com/pysam-developers/pysam/issues/939. We believe that if spacemake fails to install, it is most likely due to inconsistent packages installed. We have successfully run and installed spacemake on three separate platforms (Ubuntu 18.04, Ubuntu 20.04 and CentOS 7.9.2009) without any problems. We highly recommend following the installation instructions provided here: https://spacemake.readthedocs.io/en/latest/install.html. Briefly: (1) install mamba, (2) create a fresh conda environment using mamba and the provided environment.yaml, (3) run `pip install spacemake`.

Should the reviewer encounter additional errors during installation, we would be pleased to assist troubleshooting via log files, or by directly reporting the error through our github page.

2. A major drawback of Spacemake is that it currently does not offer solutions for the integration of imaging information, which is typically an essential step in any spatial sequencing workflow. The authors do note this shortcoming in their discussion and as a potential solution they argue that Spacemake can be used with another tool called Optocoder, which is currently being developed in their lab. However no information can be found anywhere. There is no biorxiv or github page available based on our search results and as such we were unable to test or assess this solution. At minimum the authors should provide general guidelines on how users could potentially integrate images together with the created spatial downstream results.

A: We agree with the reviewer that aligning and integrating imaging data is important when it comes to spatial transcriptomics. Therefore, we have extended spacemake with an additional module that offers an automatic image alignment algorithm. We demonstrate its usage by automatically aligning several Visium and Seq-scope datasets with their corresponding H&E images (Fig. 6D, Sup. Fig. 5, Methods)

Minor comments:
1. The figure labels and legends are not always clear. More specifically it's sometimes hard to figure out which samples are being used for each figure or panel. This could be simply resolved by writing more informative legends that specifically state which sample was used to create each figure panel. According to the text Seq-Scope was used to generate figure 3, however in the legend of figure 3 it says Slide-seq …

A: We have now corrected the figure legend of Fig. 4 (previously Fig. 3). Also, we have expanded and rewritten the legends of all figures that needed further clarification.

2. Overall, the figures are pretty and informative, however I would suggest starting with a general overview figure that highlights the spacemake pipeline and it's innovative framework. Given the goal and content of the manuscript this seems to be appropriate as a main figure.

A: We agree with the reviewer and have now moved the former Sup. Fig. 1 as Fig. 1.

3. In order to initialize a spacemake project, the dropseq tools that are required by Spacemake lack any introduction. Please provide a brief introduction and a link to the associated github page to improve this step.

A: We have now added an explanation about dropseq-tools to our docs.

4. In order to configure the spacemake project by adding a sample species, the pipeline does not allow compressed versions of genome files. This could be simply fixed and allows the user to directly link to their, typically compressed, genome files.

A: Spacemake now allows the addition of compressed genome and annotation files.

5. More information is needed about the R1 R2 arguments in the add sample function. For example, SeqScope has two separate libraries to get sequenced. Where each round of libraries should be loaded is not immediately clear from the tutorial the authors provided.

A: In the manuscript we have used a bash script written for Seq-scope (Cho et al. 2021) to generate the coordinate file from the FASTQ flowcell library. In spacemake, R1 and R2 refer to the tissue library, and that holds true also for Seq-scope. We have now updated the docs to explain this better.

6. The downsampling and NovoSparc modules together might create an opportunity to identify the relative error that is introduced when NovoSparc is used to enhance spatial expression patterns. Although this might be outside the scope of this paper.

A: We thank the reviewer for this suggestion, although it is indeed outside the scope of this paper. It would be interesting to perform this analysis and potentially identify the applicability area of the novoSpaRc integration.

7. As mentioned in the Major comments section we were unable to successfully run an example script,

but it would be of great interest to the large spatial community if this pipeline can easily be used with other downstream analysis tools, such as Giotto, Seurat, Bioconductor (spatialExperiment class), etc.

A: Spacemake creates both a text based and a compressed (.h5ad format) Digital Expression Matrix as a result of data-processing. This can be easily imported into Seurat and other downstream tools. Spacemake stores the intermediate and processed data files in the AnnData format which is immediately compatible with the Python based scanpy, or squidpy. For users that use the data structures in R, we recommend the https://github.com/theislab/zellkonverter package from the Theis lab which converts between AnnData and Bioconductor.

Close
