# Supplementary material for: Spacemake: processing and analysis of large-scale spatial transcriptomics data
Source: Gigascience. 2022 Jul 19;11:giac064. doi: 10.1093/gigascience/giac064 (PMC9295369; doi:10.1093/gigascience/giac064)
Supplement: giac064_Response_to_Reviewer_Comments_Revision_1 [file giac064_response_to_reviewer_comments_revision_1.pdf]

Author's Response To Reviewer Comments

Close

Dear Hans,

We have addressed all the editorial points as outlined.

Also, we have noticed that in the previously submitted version of the manuscript the legend of Figure 5 was out of date. Hence we have updated the legend of Figure 5.

Please do not hesitate to contact us in case you have additional requests.

Best regards,
Nikolaos Karaiskos

Close
