# Supplementary material for: Spacemake: processing and analysis of large-scale spatial transcriptomics data
Source: Gigascience. 2022 Jul 19;11:giac064. doi: 10.1093/gigascience/giac064 (PMC9295369; doi:10.1093/gigascience/giac064)
Supplement: giac064_Reviewer_1_Report_Original_Submission — Qianqian Song -- 11/29/2021 Reviewed [file giac064_reviewer_1_report_original_submission.pdf]

Reviewer Report

Title: Spacemake: processing and analysis of large-scale spatial transcriptomics data

Version: Original Submission **Date:** 11/29/2021

Reviewer name: Qianqian Song

Reviewer Comments to Author:

This manuscript proposed a python-based framework named spacemake, to process and analyze spatial transcriptomics datasets. It offers functionalities including sample merging, saturation
analysis and analysis of long-reads as separate modules, etc. Overall, this tool holds promises for spatial analysis, though this manuscript lacks details and explanations of methods and results. Specifically, I have some concerns regarding this manuscript.
1) As shown in table 1, it is noticeable that spacemake doesn't include H&E integration, which is kind of necessary in spatial data. I would recommend the authors at least discuss the potential functionality in including H&E images.
2) From the legend of Fig 2B, I didn't find the plot with Shannon entropy, please double check.
3) I don't understand the meaning of fig 2D. The authors should explain how they calculate the Shannon entropy and string compression length of the sequenced barcodes, as well as how they define the expected theoretical distributions. More details are needed here. Though the authors mentioned related information/details would be in methods (last line in QC section), I didn't find any in methods.
4) In Fig 4 A, the authors show the mapped scRNA-seq of mouse cortical layers. I think a complement spatial plot with annotations is necessary, as there is a gap between Fig 4A and Fig 4B.
5) Fig 5C lack the annotations of different colors.
6) In page 16, the authors cited a manuscript in preparation, which is not good. I suggest remove the citation.
7) Supplementary Fig 1 would be better if put as fig 1, thus it would show the overall flow & functionality of spacemake.
8) Based on Supplementary Fig 1, the authors should add a section illustrating how they annotate the spatial data and the involved gene markers.
9) The paragraph "Spacemake can readily merge resequenced samples" lacks detailed explanation and results.
10) Though spackemake claims it is fast in processing data, well, Supplementary Fig 5 doesn't fully support that. Meanwhile, the authors should explain what the different colors represent.
11) In Supplementary Fig 2, the authors show very high correlation between spacemake and spaceranger, especially the exon intron and exon sub-figures. It looks like the correlations is close to 1. I suggest the authors double check the results and give explanations on their correlation analysis.

Level of Interest

Please indicate how interesting you found the manuscript: Choose an item.

Quality of Written English

Please indicate the quality of language in the manuscript: Choose an item.

Declaration of Competing Interests

Please complete a declaration of competing interests, considering the following questions:

- Have you in the past five years received reimbursements, fees, funding, or salary from an organisation that may in any way gain or lose financially from the publication of this manuscript, either now or in the future?
- Do you hold any stocks or shares in an organisation that may in any way gain or lose financially from the publication of this manuscript, either now or in the future?
- Do you hold or are you currently applying for any patents relating to the content of the manuscript?
- Have you received reimbursements, fees, funding, or salary from an organization that holds or has applied for patents relating to the content of the manuscript?
- Do you have any other financial competing interests?
- Do you have any non-financial competing interests in relation to this paper?

If you can answer no to all of the above, write 'I declare that I have no competing interests' below. If your reply is yes to any, please give details below.

I declare that I have no competing interests

I agree to the open peer review policy of the journal. I understand that my name will be included on my report to the authors and, if the manuscript is accepted for publication, my named report including any attachments I upload will be posted on the website along with the authors' responses. I agree for my report to be made available under an Open Access Creative Commons CC-BY license (http://creativecommons.org/licenses/by/4.0/). I understand that any comments which I do not wish to be included in my named report can be included as confidential comments to the editors, which will not be published.

Choose an item.

To further support our reviewers, we have joined with Publons, where you can gain additional credit to further highlight your hard work (see: https://publons.com/journal/530/gigascience). On publication of this paper, your review will be automatically added to Publons, you can then choose whether or not to claim your Publons credit. I understand this statement.

Yes Choose an item.
