# Supplementary material for: Spacemake: processing and analysis of large-scale spatial transcriptomics data
Source: Gigascience. 2022 Jul 19;11:giac064. doi: 10.1093/gigascience/giac064 (PMC9295369; doi:10.1093/gigascience/giac064)
Supplement: giac064_Reviewer_2_Report_Original_Submission — Ruben Dries, Ph.D. -- 12/26/2021 Reviewed [file giac064_reviewer_2_report_original_submission.pdf]

Reviewer Report

Title: Spacemake: processing and analysis of large-scale spatial transcriptomics data

Version: Original Submission **Date:** 12/26/2021

Reviewer name: Ruben Dries, Ph.D.

Reviewer Comments to Author:

In this article, the authors created a modular and scalable pipeline to process raw sequencing data from spatially resolved transcriptomic technologies. In contrast to other popular genomics technologies, such as (single-cell) RNA sequencing, there are virtually no existing public tools that allow users to quickly and efficiently process the raw spatial transcriptomic sequencing data that are generated through Illumina sequencing. This is largely due to the fact that each spatial transcriptomic workflow creates its own unique spatially barcoded reads and thus typically requires technology-specific tools or scripts to extract both the barcode and gene expression information. Here the authors created Spacemake which consists of multiple modules that are tied together using the popular workflow management system Snakemake. The innovative part of Spacemake comes from the creation of specific 'sample variables', such as the barcode-flavor, run-mode and puck, which allows them to create a flexible pipeline that in theory can be adapted to any type of spatial array-based sequencing technology. The authors use well-established tools for downstream quality control and data processing and provide useful additional modules to assess or improve spatial data quality. Finally, Spacemake is also directly linked to Squidpy for downstream analysis and creates a web-based report, which could certainly help to lower initial spatial data analysis barriers.

Overall, the presentation of the tool and the methods used in the pipeline as described in their contents are comprehensive and the user manual is easy to understand. We appreciate the efforts to provide this tool to the spatial transcriptomics community and to make it open-source and flexible. However, we do have some suggestions and concerns regarding the manuscript and/or use of this tool.

Major comments:

1. We managed to install the spacemake software on the linux based server but failed to install it on a MacOS machine due to the compatibility issue with bcl2fastq2. Unfortunately, we also ran into an issue on our linux server, which happened during one of the reading steps from "/dev/stdin" in the middle of the spacemake workflow. More specifically we encountered the following error:

Job error: Job 7, TagReadWithGeneFunction

Error message: [E::idx_find_and_load] Could not retrieve index file for '/dev/stdin'

Even with the help of our IT team we were unable to resolve this issue. To help troubleshoot it might be helpful if the authors can provide exact commands for the examples provided in the manuscript and show what should be expected output of each job in the snakemake pipeline. As a result we were unable to re-run any of the provided examples, which severely limited our reviewing options.

2. A major drawback of Spacemake is that it currently does not offer solutions for the integration of imaging information, which is typically an essential step in any spatial sequencing workflow. The authors do note this shortcoming in their discussion and as a potential solution they argue that Spacemake can

be used with another tool called Optocoder, which is currently being developed in their lab. However no information can be found anywhere. There is no biorxiv or github page available based on our search results and as such we were unable to test or assess this solution. At minimum the authors should provide general guidelines on how users could potentially integrate images together with the created spatial downstream results.

Minor comments:

1. The figure labels and legends are not always clear. More specifically it's sometimes hard to figure out which samples are being used for each figure or panel. This could be simply resolved by writing more informative legends that specifically state which sample was used to create each figure panel. According to the text Seq-Scope was used to generate figure 3, however in the legend of figure 3 it says Slide-seq …
2. Overall, the figures are pretty and informative, however I would suggest starting with a general overview figure that highlights the spacemake pipeline and it's innovative framework. Given the goal and content of the manuscript this seems to be appropriate as a main figure.
3. In order to initialize a spacemake project, the dropseq tools that are required by Spacemake lack any introduction. Please provide a brief introduction and a link to the associated github page to improve this step.
4. In order to configure the spacemake project by adding a sample species, the pipeline does not allow compressed versions of genome files. This could be simply fixed and allows the user to directly link to their, typically compressed, genome files.
5. More information is needed about the R1 R2 arguments in the add sample function. For example, SeqScope has two separate libraries to get sequenced. Where each round of libraries should be loaded is not immediately clear from the tutorial the authors provided.
6. The downsampling and NovoSparc modules together might create an opportunity to identify the relative error that is introduced when NovoSparc is used to enhance spatial expression patterns. Although this might be outside the scope of this paper.
7. As mentioned in the Major comments section we were unable to successfully run an example script, but it would be of great interest to the large spatial community if this pipeline can easily be used with other downstream analysis tools, such as Giotto, Seurat, Bioconductor (spatialExperiment class), etc.

Level of Interest

Please indicate how interesting you found the manuscript: Choose an item.

Quality of Written English

Please indicate the quality of language in the manuscript: Choose an item.

Declaration of Competing Interests

Please complete a declaration of competing interests, considering the following questions:

- Have you in the past five years received reimbursements, fees, funding, or salary from an organisation that may in any way gain or lose financially from the publication of this manuscript, either now or in the future?
- Do you hold any stocks or shares in an organisation that may in any way gain or lose financially from the publication of this manuscript, either now or in the future?
- Do you hold or are you currently applying for any patents relating to the content of the manuscript?
- Have you received reimbursements, fees, funding, or salary from an organization that holds or has applied for patents relating to the content of the manuscript?
- Do you have any other financial competing interests?
- Do you have any non-financial competing interests in relation to this paper?

If you can answer no to all of the above, write 'I declare that I have no competing interests' below. If your reply is yes to any, please give details below.

I declare that I have no competing interests

I agree to the open peer review policy of the journal. I understand that my name will be included on my report to the authors and, if the manuscript is accepted for publication, my named report including any attachments I upload will be posted on the website along with the authors' responses. I agree for my report to be made available under an Open Access Creative Commons CC-BY license (http://creativecommons.org/licenses/by/4.0/). I understand that any comments which I do not wish to be included in my named report can be included as confidential comments to the editors, which will not be published.

Choose an item.

To further support our reviewers, we have joined with Publons, where you can gain additional credit to further highlight your hard work (see: https://publons.com/journal/530/gigascience). On publication of this paper, your review will be automatically added to Publons, you can then choose whether or not to claim your Publons credit. I understand this statement.

Yes Choose an item.
